# Supplementary material for: Deregulating the CYP2C19/Epoxy-Eicosatrienoic Acid-Associated FABP4/FABP5 Signaling Network as a Therapeutic Approach for Metastatic Triple-Negative Breast Cancer
Source: Cancers (Basel). 2020 Jan 13;12(1):199. doi: 10.3390/cancers12010199 (PMC7016875; doi:10.3390/cancers12010199)
Supplement: Supplementary file 1 [file cancers-12-00199-s001.pdf]

Supplementary Materials

Deregulating the CYP2C19/Epoxy-Eicosatrienoic Acid-Associated FABP4/FABP5 Signaling Network as A Therapeutic Approach for Metastatic Triple-Negative Breast Cancer

Maria Karmella Apaya, Pei-Wen Hsiao, Yu-Chih Yang, and Lie-Fen Shyur

Table S1. Top 10 pathways significantly upregulated in histologically classified TNBC (ER–/PR–/HER2–) tumor samples with mRNA expression of CYP2J2/FABP4/FABP5 *z*-scores ≥ 2.0. Pathways and gene lists were downloaded from the Panther and Reactome databases.

Pathway	Enrichment ratio	*P* value	FDR*
Chemokine signaling	11.7	2.81e-07	2.24e-04
Signaling by interleukins	5.22	8.49e-08	1.35e-04
PPAR signaling pathway	8.18	1.94e-06	1.03e-03
Phase 1 - Functionalization of compounds	8.30	3.24e-04	7.38e-02
EPH-Ephrin signaling	9.63	1.62e-04	4.89e-02
Extracellular matrix organization	4.96	1.84e-04	4.89e-02
Triglyceride metabolism	21.9	3.22e-04	5.90e-02
Arachidonic acid metabolism	6.79	9.60e-03	3.13e-01
Regulation of adipocyte differentiation	7.92	6.27e-03	2.91e-01
Developmental biology	2.51	5.73e-04	1.02e-01

*: FDR: false discovery rate.

Table S2. Oxylipin metabolites identified in the tumor tissues of mice implanted with metastatic MDA-MB-231 cells (231-iR2L and lung-seeking LM6) and FABP4/FABP5/CYP2C19-depleted LM6 cells.

Metabolite/Group	231-iR2L	LM6-shLacZ	LM6-shFABP4	LM6-shFABP5	LM6-shCYP2C19
CYP450 epoxygenases and hydroxylases					
AA metabolites					
5,6-EET	62.7 ± 5.3^{a}	89.7 ± 10.7^{b}	73.3 ± 14.8^{ab}	85.8 ± 9.5^{b}	62.5 ± 9.6^{a}
8,9-EET	122.6 ± 12.2^{a}	162.6 ± 23.9^{b}	153.3 ± 22.2^{abc}	147.3 ± 9.0^{bc}	106.8 ± 17.6^{ac}
11,12-EET	58.1 ± 11.6^{ab}	92.7 ± 18.5^{b}	73.3 ± 14.8^{ab}	85.8 ± 9.5^{ab}	62.5 ± 9.6^{a}
14,15-EET	145.5 ± 11.4^{ac}	160.3 ± 12.5^{ad}	146.6 ± 24.3^{acd}	139.8 ± 7.8^{b}	127.3 ± 15.5^{cd}
19-HETE	122.4 ± 14.6^{ab}	115.5 ± 13.6^{ab}	131.7 ± 6.5^{ab}	139.2 ± 5.5^{a}	108.1 ± 11.2^{b}
20-HETE	131 ± 22.8^{a}	177.6 ± 18.7^{a}	117.5 ± 23.8^{a}	142.2 ± 28.3^{a}	140.2 ± 24.9^{a}
LA metabolites					
9,10-EpOME	36.4 ± 10.2^{a}	47.3 ± 9.2^{a}	33.7 ± 7.8^{a}	33.6 ± 9.0^{a}	33.4 ± 6.8^{a}
12,13-EpOME	38.1 ± 6.7^{a}	43.8 ± 3.4^{a}	42.5 ± 8.6^{a}	57 ± 11.7^{a}	58.1 ± 17.0^{a}
Soluble epoxide hydrolase					
AA metabolites					
5,6-DHET	13.7 ± 4.9^{a}	11.1 ± 3.3^{a}	18.1 ± 1.5^{a}	11.3 ± 4.7^{a}	19 ± 17.9^{a}
8,9-DHET	45.5 ± 3.1^{ab}	46 ± 4.2^{ab}	53.8 ± 6.1^{a}	35.6 ± 7.6^{bc}	23.7 ± 2.1^{c}
11,12-DHET	27.2 ± 6.2^{b}	19.7 ± 6.0^{b}	61.5 ± 15.6^{a}	35.8 ± 1.4^{b}	22.9 ± 6.9^{b}
14,15-DHET	17.1 ± 1.8^{a}	10.7 ± 8.4^{a}	13 ± 7.1^{a}	19.5 ± 6.0^{a}	10.9 ± 4.0^{a}
LA metabolites					
9,10-DHOME	45.2 ± 6.4^{a}	41.8 ± 2.1^{a}	49.1 ± 9.7^{a}	51.3 ± 8.4^{a}	62.8 ± 14.5^{a}
12,13-DHOME	28.4 ± 4.2^{a}	29 ± 2.2^{a}	26.7 ± 2.7^{a}	36.1 ± 23.9^{b}	37.9 ± 8.8^{b}
Cycloxygenase					
AA metabolites					
PGE_2	116.6 ± 18.3^{b}	216.7 ± 16.5^{a}	201.6 ± 15.0^{a}	116.3 ± 21.6^{b}	116.9 ± 20.5^{b}
Lipoxygenases					
AA metabolites					
5-HETE	133.7 ± 11.4^{b}	216.7 ± 9.5^{a}	126.7 ± 15.1^{b}	122.5 ± 13.2^{b}	116.6 ± 10.2^{b}
8-HETE	147.8 ± 6.7^{b}	162.2 ± 17.8^{b}	232 ± 12.2^{a}	183.6 ± 17.7^{b}	192.1 ± 23.7^{ab}
9-HETE	126.5 ± 14.9^{b}	118.9 ± 11.2^{b}	217.1 ± 16.1^{a}	182.2 ± 15.6`	117.3 ± 12.8^{b}
11-HETE	217.2 ± 11.7^{a}	218.5 ± 13.0^{a}	117.9 ± 19.8^{b}	206.7 ± 19.2`	217.4 ± 15.5^{a}
12-HETE	192.1 ± 12.6^{b}	270.6 ± 18.8^{a}	148.6 ± 10.8^{c}	153.8 ± 11.6^{c}	204.8 ± 12.3^{b}
15-HETE	217.4 ± 14.2^{a}	146.3 ± 15.9^{bc}	212.7 ± 19.4^{a}	117.2 ± 17.1^{c}	171.4 ± 19.8^{ab}
LTA_4	121.2 ± 12.9^{c}	249.2 ± 10.8^{a}	187.2 ± 14.2^{b}	183 ± 41.3^{b}	158 ± 7.8^{bc}
LA metabolites					
9-HODE	43.1 ± 14.2^{c}	62.9 ± 13.9^{ab}	32 ± 13.8^{bc}	73.6 ± 5.2^{ab}	89.2 ± 8.0^{e}
13-HODE	16.2 ± 8.2^{b}	38.52 ± 9.2^{ab}	67.61 ± 19.8^{a}	56.62 ± 2.7^{a}	47.23 ± 7.9^{a}
9-oxoODE	72.5 ± 1.2^{ab}	48.5 ± 12.6^{bc}	98.2 ± 16.0^{a}	26.7 ± 3.2^{c}	82.4 ± 19.0^{a}
13-oxoODE	49.1 ± 11.2^{a}	70 ± 11.6^{b}	48.2 ± 5.1^{a}	53.2 ± 12.6^{a}	62.7 ± 7.4^{a}

Tumor tissues were collected and flash-frozen in -80°C immediately. C immediately. Groups are defined as mice inoculated with 231-iR2L, LM6, LM6-shFABP4, LM6-shFABP5 and LM6-shCYP2C19 cells. Data are means ± SEM (ng/100 mg; $N = 4$, $P < 0.05$, ANOVA, *post hoc* Tukey). Values with different letters are significantly different.

Table S3. Oxylipin metabolites identified in the lung tissues of mice implanted with MDA-MB-231 (231-iR2L and lung-seeking LM6), and FABP4/FABP5/CYP2C19-depleted LM6 cells.

Metabolite/Group	231-iR2L			LM6-shLacZ			LM6-shFABP4			LM6-shFABP5			LM6-shCYP2C19		
CYP450 epoxygenases and hydroxylases															
AA metabolites															
5,6-EET	108.0	±	11.3^{a}	149.1	±	28.4^{ac}	85.8	±	15.3^{ab}	192.8	±	33.3^{d}	122.8	±	23.6^{a}
8,9-EET	183.0	±	17.6^{a}	192.2	±	31.4^{a}	233.8	±	33.3^{ab}	237.9	±	19.4^{b}	120.9	±	35.5^{c}
11,12-EET	134.1	±	16.7^{a}	200.4	±	40.7^{ab}	126.4	±	29.8^{ac}	278.2	±	60.9^{d}	111.5	±	26.0^{ac}
14,15-EET	145.9	±	11.1^{a}	198.5	±	31.3^{b}	113.2	±	16.2^{a}	243.7	±	36.5^{b}	130.5	±	23.5^{ac}
19-HETE	122.7	±	11.1^{ab}	109.4	±	6.1^{ab}	123.6	±	8.1^{ab}	140.5	±	11.0^{a}	122.9	±	13.8^{b}
20-HETE	148.5	±	15.0^{ab}	158.2	±	19.4^{a}	98.9	±	18.6^{b}	141.4	±	21.9^{ab}	167.8	±	15.0^{ab}
LA metabolites															
9,10-EpOME	37.2	±	3.2^{a}	41.1	±	6.2^{a}	39.5	±	5.8^{a}	33.6	±	5.1^{a}	32.1	±	11.3^{a}
12,13-EpOME	53.7	±	9.2^{a}	35.4	±	8.4^{a}	57.6	±	15.1^{a}	57	±	17.1^{a}	46.7	±	5.4^{a}
Soluble epoxide hydrolase															
AA metabolites															
5,6-DHET	17.4	±	15.1^{a}	26.4	±	6.4^{a}	24.1	±	6.9^{a}	18.1	±	1.3^{a}	31.7	±	10.3^{a}
8,9-DHET	57.1	±	7.5^{a}	37.5	±	9.7^{a}	46.7	±	16.2^{a}	53.8	±	6.8^{a}	56.8	±	13.5^{b}
11,12-DHET	45.7	±	10.0^{b}	28.1	±	8.5^{b}	62.3	±	7.8^{a}	61.5	±	7.4^{a}	43.1	±	14.5^{b}
14,15-DHET	34.0	±	8.2^{b}	46.1	±	7.7^{a}	37.1	±	3.4^{b}	16.7	±	9.4^{a}	35.7	±	7.1^{b}
LA metabolites															
9,10-DHOME	49.4	±	4.6^{a}	50.2	±	8.4^{a}	56.9	±	7.8^{a}	41.9	±	9.4^{a}	49.1	±	20.0^{a}
12,13-DHOME	36.9	±	5.5^{a}	36.8	±	7.8^{a}	29.6	±	2.9^{a}	26.1	±	10.0^{a}	26.7	±	10.3^{a}
Cycloxygenase															
AA metabolites															
PGE$_2$	143.3	±	5.9^{b}	152.7	±	9.9^{a}	138.1	±	9.5^{a}	126.7	±	5.2^{b}	118.2	±	5.1^{b}
Lipoxygenases															
AA metabolites															
5-HETE	143.3	±	15.9^{a}	238.1	±	21.4^{b}	109.6	±	17.1^{a}	126.7	±	7.7^{a}	124.9	±	3.5^{a}
8-HETE	148.5	±	14.1^{c}	178.9	±	16.7^{bc}	216.3	±	15.7^{a}	232	±	13.2^{bc}	203.7	±	11.4^{b}
9-HETE	129.6	±	13.5^{b}	134.3	±	15.4^{a}	198	±	19.1^{a}	217.1	±	19.8^{a}	151.2	±	18.8^{b}
11-HETE	229.1	±	12.6^{a}	233	±	14.5^{b}	104.5	±	13.4^{a}	117.9	±	11.0^{a}	232.7	±	14.6^{a}
12-HETE	203.0	±	19.7^{bc}	289.5	±	18.9^{a}	125.6	±	23.0^{c}	148.6	±	18.9^{bc}	213.6	±	22.8^{b}
15-HETE	223.3	±	13.9^{a}	166.3	±	20.0^{bc}	190.7	±	22.0^{a}	212.7	±	18.7^{c}	166.1	±	13.3^{ab}
LTA$_4$	121.5	±	18.2^{c}	226.6	±	21.1^{a}	192.1	±	17.4^{b}	201.6	±	19.3^{b}	124.5	±	22.8^{bc}
LA metabolites															
9-HODE	60.1	±	16.5^{bc}	73.4	±	17.0^{abc}	21.4	±	7.2^{c}	70.7	±	14.7^{ab}	99.8	±	11.5^{a}
13-HODE	23.4	±	4.5^{b}	48.3	±	19.5^{ab}	39.7	±	10.8^{a}	45.5	±	12.4^{a}	84.9	±	19.3^{ab}
9-oxoODE	79.7	±	7.3^{ab}	37.9	±	12.2^{bc}	86.0	±	13.9^{a}	4.5	±	1.7^{c}	71.6	±	13.4^{a}
13-oxoODE	67.9	±	11.6^{a}	59.7	±	14.3^{b}	27.5	±	8.4^{a}	54.3	±	12.4^{a}	83.9	±	15.8^{a}

Whole lung tissues were collected, flash-frozen and stored in -80°C immediately. Groups are defined as mice inoculated with 231-iR2L, LM6, LM6-shFABP4, LM6-shFABP5 and LM6-shCYP2C19 cells. Data are means ± SEM (ng/100 mg tissue; $N = 4$, $P < 0.05$, ANOVA, *post hoc* Tukey). Values with different letters are significantly different.

Table S4. Effects of dLGG, doxorubicin or doxorubicin+dLGG combination treatment on the oxylipin metabolite concentrations identified in the tumor tissues of mice implanted with metastatic MDA-MB-231 cells (LM6).

Metabolite/Group	**Tumor control**			**dLGG**			**DOX5**			**dLGG25+DOX5**		
					CYP450 epoxygenases and hydroxylases							
					AA metabolites							
5,6-EET	113.1	±	10.1[a]	81.2	±	8.3[b]	83.7	±	11.5[b]	56.6	±	3.1[c]
8,9-EET	145.8	±	26.4[a]	100.7	±	8.0[b]	100.3	±	7.3[b]	103.8	±	12.7[b]
11,12-EET	140.4	±	17.3[a]	60.3	±	11.1[b]	86.4	±	18.8[b]	69.7	±	6.2[b]
14,15-EET	133.7	±	5.7[a]	88.0	±	13.0[b]	110.8	±	5.4[b]	85.4	±	9.2[b]
19-HETE	189.2	±	9.7[a]	178	±	10.3[a]	176	±	12.8[a]	168.3	±	11.1[a]
20-HETE	117.9	±	20.4[a]	98.4	±	20.2[a]	109.8	±	16.4[a]	109.1	±	18.4[a]
					LA metabolites							
9,10-EpOME	112.5	±	18.3[a]	101.8	±	12.0[a]	112.7	±	19.5[a]	98.6	±	21.2[a]
12,13-EpOME	39	±	9.9[b]	54.1	±	6.7[a]	67.3	±	14.5[a]	59.3	±	13.3[a]
					Soluble epoxide hydrolase							
					AA metabolites							
5,6-DHET	25.8	±	16.1[b]	31.6	±	15.2[ab]	65.7	±	18.6[a]	42.1	±	8.5[ab]
8,9-DHET	56.9	±	18.5[a]	67	±	12.7[a]	78.1	±	18.1[a]	46.8	±	18.5[a]
11,12-DHET	35.6	±	20.7[a]	47.1	±	16.4[a]	51.9	±	13.6[a]	34.9	±	15.9[a]
14,15-DHET	19	±	8.0[a]	28.6	±	11.8[a]	45.2	±	6.2[b]	45.3	±	7.6[b]
					LA metabolites							
9,10-DHOME	24.5	±	13.6[a]	21	±	15.8[a]	13.3	±	20.8[a]	18.7	±	21.7[a]
12,13-DHOME	34.1	±	6.1[bc]	52.1	±	10.8[ab]	67	±	8.2[a]	29	±	9.5[c]
					Cycloxygenases							
					AA metabolites							
PGD_2	26	±	1.1[a]	43.9	±	10.0[b]	12.4	±	20.2[a]	32.9	±	15.9[ab]
PGE_2	39.1	±	16.9[a]	64	±	18.4[b]	56	±	11.9[b]	45	±	16.5[a]
PGB_2/PGJ_2	49	±	13.4[a]	52	±	17.5[a]	39.8	±	12.6[a]	31.2	±	12.3[c]
15-deoxy-PGJ_2	13.7	±	12.9[a]	21	±	10.6[a]	18	±	9.0[a]	27.2	±	7.4[b]
					Lipoxygenases							
					AA metabolites							
5-HETE	105.8	±	20.6[a]	89.5	±	1.7[ab]	99.4	±	18.9[ab]	65.7	±	2.1[b]
8-HETE	67.8	±	13.4[a]	56.9	±	14.7[a]	53.9	±	16.4[a]	43.9	±	10.7[a]
9-HETE	59.1	±	16.7[a]	42	±	13.4[b]	64.3	±	13.4[a]	62.7	±	17.7[a]
11-HETE	117.2	±	13.7[a]	129.3	±	5.0[b]	110.4	±	5.8[b]	109.5	±	12.2[b]
12-HETE	137.3	±	1.5[a]	120.5	±	15.1[b]	172.8	±	19.0[b]	104.5	±	11.6[a]
15-HETE	161.5	±	17.9[a]	90.8	±	15.8[a]	104.2	±	19.0[b]	156.9	±	17.1[a]

5-HETE	139	±	19.3[a]	120.7	±	21.3[b]	173.3	±	13.2[b]	112.3	±	18.4[c]
9-HODE	92.5	±	13.8[ab]	56.7	±	15.0[b]	119.5	±	11.5[a]	72.3	±	15.7[b]
13-HODE	84	±	20.3[a]	75.8	±	12.1[a]	104.9	±	18.5[a]	65.4	±	17.3[a]
9-oxoODE	82.8	±	13.6[a]	72.9	±	7.0[a]	97	±	10.0[b]	58.9	±	6.7[a]
13-oxoODE	77.5	±	15.1[ab]	52.1	±	14.7[bc]	87.4	±	10.9[b]	41.2	±	9.2[c]

Tumor tissues were collected, flash-frozen and stored in -80°C immediately. Groups are defined as mice inoculated with metastatic TNBC cells (LM6 tumor control) and mice treated with dLGG25, DOX5, and dLGG25+DOX5. Data are means ± SEM (ng/100 mg tissue; $N = 4$, $P < 0.05$, ANOVA, *post hoc* Tukey). Values with different letters are significantly different.

Table S5. Effects of dLGG, doxorubicin or doxorubicin+dLGG treatment on the oxylipin metabolite concentrations identified in the lung tissues of mice implanted with metastatic MDA-MB-231 cells (LM6).

Metabolite/Group	**Sham**			**Tumor control**			**dLGG25**			**DOX5**			**dLGG25+DOX5**		
							CYP450 epoxygenases and hydroxylases								
							**AA metabolites**								
**5,6-EET**	**145.4**	**±**	**14.5[a]**	**168.8**	**±**	**15.4[a]**	**141.6**	±	12.7[ab]	150.2	±	16.4[ab]	141.5	±	15.2[ab]
8,9-EET	150.2	±	24.6[a]	209.6	±	36.0[b]	135.5	±	11.4[a]	193.6	±	15.1[a]	147.6	±	17.1[a]
11,12-EET	135.2	±	14.0[a]	156.3	±	18.0[ab]	119.9	±	6.6[ac]	130.2	±	12.0[ac]	110.7	±	5.4[c]
14,15-EET	121.1	±	15.9[a]	187.6	±	13.8[b]	145.1	±	17.1[a]	125.0	±	19.4[a]	126.9	±	18.9[a]
19-HETE	89.5	±	5.8[a]	148.9	±	6.0[a]	112.9	±	14.3[a]	125.8	±	8.6[a]	137.9	±	13.8[a]
20-HETE	134.5	±	16.1[b]	173.4	±	13.6[b]	142.9	±	18.7[b]	154.9	±	13.1[a]	145.6	±	12.1[a]
							**LA metabolites**								
9,10-EpOME	98	±	16.8[c]	89	±	11.3[bc]	112.4	±	10.2[a]	156.3	±	15.5[b]	154	±	18.7[bc]
12,13-EpOME	78.3	±	9.9[bc]	96.2	±	16.9[a]	190.3	±	14.9[c]	118.4	±	11.2[b]	110.5	±	13.9[b]
							Soluble epoxide hydrolase								
							**AA metabolites**								
5,6-DHET	129	±	3.3[b]	190.3	±	19.1[b]	94.1	±	7.4[c]	145	±	20.2[a]	143.8	±	9.8[bc]
8,9-DHET	125.7	±	18.9[a]	124.5	±	12.5[a]	78	±	12.5[b]	176.2	±	14.7[a]	105.9	±	11.1[a]
11,12-DHET	209.4	±	16.9[a]	165	±	12.9[a]	116.5	±	16.6[a]	165.5	±	19.7[b]	210.3	±	19.4[c]
14,15-DHET	172.1	±	5.6[c]	183.8	±	13.4[c]	189.1	±	12.9[bc]	143.9	±	8.4[ab]	106.8	±	6.9[a]
							**LA metabolites**								
9,10-DHOME	65.9	±	15.2[a]	68.4	±	19.9[a]	79	±	20.7[a]	83.4	±	18.5[a]	54.6	±	14.8[a]
12,13-DHOME	34.7	±	6.3[bc]	37.9	±	9.1[bc]	56	±	8.5[ab]	65.9	±	6.6[a]	34	±	9.0[c]
							Cycloxygenases								
							**AA metabolites**								
PGD_2	53.8	±	8.8[c]	135.8	±	8.0[ab]	67.4	±	17.3[c]	154	±	6.3[a]	109.5	±	16.8[b]
PGE_2	109.3	±	15.1[b]	189.5	±	13.4[a]	89.5	±	17.1[b]	160.2	±	12.9[a]	78.3	±	19.5[b]
PGB_2/PGJ_2	78	±	19.0[b]	108	±	20.9[ab]	65.2	±	18.2[b]	153.2	±	18.0[a]	150.3	±	20.6[a]
15-deoxy-PGJ_2	58.4	±	13.1[b]	127.3	±	6.3[a]	67.9	±	14.4[b]	118.5	±	7.0[a]	118.3	±	13.3[a]
							Lipoxygenases								
							**AA metabolites**								
5-HETE	129.4	±	18.6[bc]	210.6	±	14.6[a]	105.5	±	20.3[bc]	87.4	±	20.5[c]	138.5	±	5.4[b]

8-HETE	78.5	±	17.8^{b}	130.4	±	13.1^{a}	104.2	±	10.3^{ab}	109.3	±	16.3^{ab}	98.3	±	14.4^{ab}
9-HETE	18.6	±	12.7^{a}	23	±	14.5^{a}	28.4	±	16.3^{a}	30.5	±	13.2^{a}	35.2	±	12.9^{a}
11-HETE	56.9	±	12.1^{a}	72.5	±	9.6^{ab}	67	±	11.6^{ab}	73.5	±	8.3^{ab}	87.3	±	9.6^{a}
12-HETE	109.6	±	6.2^{d}	262.5	±	2.5^{a}	164.9	±	9.7^{bc}	158.4	±	19.4^{c}	190.4	±	12.4^{b}
15-HETE	196.8	±	17.4^{a}	205.3	±	13.0^{a}	189.3	±	19.0^{a}	189.3	±	10.3^{a}	116.7	±	19.9^{b}
5-HETE	173.4	±	18.2^{ab}	163.7	±	14.0^{ab}	133.6	±	13.0^{b}	195.9	±	14.0^{a}	185.5	±	18.6^{a}
LTA_4	104.6	±	10.9^{a}	83	±	8.1^{a}	98.3	±	8.7^{a}	89.6	±	8.5^{a}	83.7	±	6.5^{a}
**LA metabolites**															
9-HODE	31.4	±	12.3^{a}	29.3	±	10.5^{a}	34.7	±	4.0^{a}	54.3	±	7.2^{a}	36.7	±	17.2^{a}
13-HODE	29.5	±	19.9^{a}	43	±	14.8^{a}	51.3	±	14.4^{a}	39	±	19.9^{a}	63.1	±	14.3^{a}
9-oxoODE	27	±	15.6^{a}	42.5	±	18.1^{a}	32.6	±	21.2^{a}	43.7	±	13.6^{a}	30.5	±	16.8^{a}
13-oxoODE	104.4	±	8.7^{a}	76.6	±	13.3^{ab}	86.4	±	6.4^{bc}	57.5	±	6.4^{bc}	79.5	±	6.4^{c}

Whole lung tissues were collected, flash-frozen and stored in -80°C immediately. Groups are defined as Sham control mice, mice inoculated with metastatic TNBC cells (LM6 tumor control) and mice treated with dLGG25, DOX5, and dLGG25+DOX5. Data are means ± SEM (ng/100 mg tissue; $N = 4$, $P < 0.05$, ANOVA, *post hoc* Tukey). Values with different letters are significantly different.

Table S6. shRNA information in gene knockdown analysis.

shRNA	Target Gene	Gene ID	shRNA Clone
shCYP2C19-1	CYP2C19	CYP2C19	TRCN0000064058
shCYP2C19-2	CYP2C19	CYP2C19	TRCN0000432428
shFABP4-1	FABP4	FABP4	TRCN0000059618
shFABP4-2	FABP4	FABP4	TRCN0000059622
shFABP5-1	FABP5	FABP5	TRCN0000059699
shFABP5-1	FABP5	FABP5	TRCN0000419924
shLacZ	LacZ	LacZ(control)	TRCN0000001339

Table S7. Primer information for the qRT-PCR.

Gene	Primer
CYP2C19	F:5′-CTTCTGTCCCGCCCTTCTATC-3′<br>R:5′-GATAGTGAAATTTGGACCAGAGGA-3′
FABP4	F:5′-GAAGGGCTTAGAGGAACGCA-3′<br>R:5′-AGCGTTCTCCGAAGGTGATG-3′
FABP5	F:5′-GAAGGGCTTAGAGGAACGCA-3′<br>R:5′-AGCGTTCTCCGAAGGTGATG-3′);
GADPH	F:5′-AGGGCTGCTTTTAACTCTGGT-3′<br>R :5′-CCCCACTTGATTTTGGAGGGA-3′

Table S8. Antibody information for the western blot and immunohistochemistry analysis.

Antibody	Catalog Number	Dilution Ratio	Company
c-myc	ab32	Western blotting (1:1000)	
Src	ab133283	Western blotting (1:1000)	abcam
PPAR-δ	ab23673	Western blotting (1:2000)	
ezrin	3145	Western blotting (1:1000)	
FAK	1688	Western blotting (1:2000)	
p-FAK	3282S	Western blotting (1:1000)	Cell Signaling
Ki67	9027S	IHC (1:200)	Technologies
PPAR-γ	2443	Western blotting (1:1000)	
p-Src	2105S	Western blotting (1:2000)	
		Western blotting (1:2000)	
CD44	217594	Western blotting (1:1000)/IHC	
FABP4	15872-1-AP	(1:200)	
FABP5	12348-1-AP	Western blotting (1:1000)/IHC	Proteintech
Sox-2	11064-1-AP	(1:200)	
vimentin	10366-1-AP	Western blotting (1:2000)	
		Western blotting (1:2000)	
CD36	sc-7309	Western blotting (1:2000)	
C/EBPα CYP2C9/19	sc-9315	Western blotting (1:1000)	
LXRα/β	sc-23436	Western blotting (1:1000)	
MMP-9	sc-13068	Western blotting (1:1000)	
PPAR-α	sc-6840	Western blotting (1:1000)	
PPAR-β	sc-398394	Western blotting (1:1000)	Santa Cruz Biotech
PXR	sc-74517	Western blotting (1:1000)	
RhoA	sc-25381	Western blotting (1:1000)	
RARα	sc-418	Western blotting (1:1000)	
RXRα	sc-515796	Western blotting (1:1000)	
	sc-515929	Western blotting (1:500)	
actin	mab1501	Western blotting (1:1000)	Millipore

Table S9. Densitometry analysis comparing the protein expressions of FABP4, FABP5, CYP2C19 and related metastasis-associated markers in parental MDA-MB-231 (231) and MDA-MB-231 with luciferase and fluorescence reporter genes (231) and lung-seeking metastatic subclones of iR2L (LM1 to LM6) (**Figure 2b**). Densitometry software (ImageJ) was used to determine the band intensities for each protein of interest (POI). Band intensities of the corresponding housekeeping protein (actin) were used as loading control (LC) for each lane. Normalized protein intensity for each POI was calculated by multiplying the band intensity by the loading control normalization ratio (ratio of actin intensity in the parental 231 control lane to the actin intensity in the POI lane). Relative protein expression for each POI was calculated as fold change relative to the normalized protein intensity in the parental 231 cells. Mean relative protein expressions and standard deviations for three independent western blotting experiments were obtained and statistical analysis was performed using one-way ANOVA, *post hoc* Dunnett's test, with protein expression of the parental 231 cells as control. Red color indicates $p < 0.05$ (> control).

**Protein/Experiment**		**Protein/Band Densitometry Intensity From ImageJ**								**Normalized Protein Intensity (POI Intensity*Lane Normalization Ratio)**								**Relative Protein Expression (Fold Change Relative to Expression in 231 Cells)**							
		**231**	**iR2L**	**LM1**	**LM2**	**LM3**	**LM4**	**LM5**	**LM6**	**231**	**iR2L**	**LM1**	**LM2**	**LM3**	**LM4**	**LM5**	**LM6**	**231**	**iR2L**	**LM1**	**LM2**	**LM3**	**LM4**	**LM5**	**LM6**
CYP2C19	1	2256	2405	1630	1840	2338	3524	3948	3891	2256	2443	1666	1979	2473	3836	3953	4223	1.00	1.08	0.74	0.88	1.10	1.70	1.75	1.87
(54kDa)	2	4855	7756	3603	4030	5016	11221	8351	12411	4855	8007	3738	4038	4977	10989	8598	12905	1.00	1.65	0.77	0.83	1.03	2.26	1.77	2.66
	3	12703	15837	9985	10337	15865	17810	23398	23940	12703	16192	10075	11087	16532	17404	24062	24644	1.00	1.27	0.79	0.87	1.30	1.37	1.89	1.94
	Mean									6605	8881	5159	5701	7994	10743	12205	13924	1.00	1.34	0.77	0.86	1.14	1.78	1.81	2.16
	SD									5439	6916	4381	4776	7499	6787	10528	10249		0.29	0.03	0.03	0.14	0.45	0.08	0.44
										Lane normalization ratio: actin intensity parental 231 cells (control lane)/actin intensity POI (POI lane)															
actin	1	7927	7804	7758	7369	7494	7283	7917	7304	1.00	0.98	0.98	0.93	0.95	0.92	1.00	0.92								
	2	20903	20535	20481	20880	20996	21151	20566	20454	1.00	0.97	0.96	1.00	1.01	1.02	0.97	0.96								
	3	16100	15747	15956	15011	15450	16476	15655	15640	1.00	0.98	0.99	0.93	0.96	1.02	0.97	0.97								
FABP4	1	962	645	960	561	855	1071	1203	1121	962	655	981	603	904	1166	1205	1217	1.00	0.68	1.02	0.63	0.94	1.21	1.25	1.26
(15 kDa)	2	3564	2436	3608	2173	3287	3084	1929	4035	3564	2515	3743	2177	3261	3020	1986	4195	1.00	0.71	1.05	0.61	0.92	0.85	0.56	1.18
	3	9545	7640	7563	6566	7143	9680	8006	8877	9545	7811	7631	7042	7443	9459	8233	9138	1.00	0.82	0.80	0.74	0.78	0.99	0.86	0.96
	Mean									4690	3660	4118	3274	3870	4548	3808	4850	1.00	0.74	0.96	0.66	0.88	1.02	0.89	1.13

	SD									4401	3713	3341	3357	3312	4353	3852	4001		0.07	0.14	0.07	0.09	0.18	0.35	0.16
										Lane normalization ratio: actin intensity parental 231 cells (control lane)/actin intensity POI (POI lane)															
actin	1	7927	7804	7758	7369	7494	7283	7917	7304	1.00	0.98	0.98	0.93	0.95	0.92	1.00	0.92								
	2	20903	20535	20481	20880	20996	21151	20566	20454	1.00	0.97	0.96	1.00	1.01	1.02	0.97	0.96								
	3	16100	15747	15956	15011	15450	16476	15655	15640	1.00	0.98	0.99	0.93	0.96	1.02	0.97	0.97								
FABP5	1	5351	5756	5437	6914	7037	7849	10555	10550	5351	5933	5783	7574	8099	8546	10510	10792	1.00	1.11	1.08	1.42	1.51	1.60	1.96	2.02
(15 kDa)	2	11068	7816	11205	12286	10844	20171	21601	32344	11068	7678	10887	12343	10899	20016	21050	31828	1.00	0.69	0.98	1.12	0.98	1.81	1.90	2.88
	3	30974	32519	24858	30017	33497	43058	57293	56477	30974	30278	23883	31172	31892	39090	53609	51208	1.00	0.98	0.77	1.01	1.03	1.26	1.73	1.65
	Mean									15798	14630	13518	17030	16963	22551	28389	31276	1.00	0.93	0.95	1.18	1.18	1.56	1.87	2.18
	SD									13450	13580	9332	12478	13004	15429	22468	20214		0.21	0.16	0.21	0.29	0.28	0.12	0.63
										Lane normalization ratio: actin intensity parental 231 cells (control lane)/actin intensity POI (POI lane)															
actin	1	8721	8461	8199	7961	7578	8010	8759	8526	1.00	0.97	0.94	0.91	0.87	0.92	1.00	0.98								
	2	30401	30934	31270	30264	30252	30630	31179	30883	1.00	1.02	1.03	1.00	0.99	1.01	1.03	1.02								
	3	22075	23709	22976	21257	23186	24316	23592	24347	1.00	1.07	1.04	0.96	1.05	1.10	1.07	1.10								
Vimentin	1	495	497	502	503	505	915	1221	1546	495	512	534	551	581	996	1216	1581	1.00	1.03	1.08	1.11	1.17	2.01	2.46	3.19
(57 kDa)	2	3263	2731	3197	2883	2855	3419	3869	4959	3263	2683	3106	2896	2869	3393	3770	4880	1.00	0.82	0.95	0.89	0.88	1.04	1.16	1.50
	3	4744	4703	3988	4618	5258	5696	5347	6310	4744	4379	3832	4796	5006	5171	5003	5721	1.00	0.92	0.81	1.01	1.06	1.09	1.05	1.21
	Mean								ss	2834	2525	2491	2748	2819	3187	3330	4061	1.00	0.93	0.95	1.00	1.04	1.38	1.56	1.97
	SD									2157	1938	1733	2126	2213	2095	1932	2188		0.11	0.14	0.11	0.15	0.55	0.78	1.07
										Lane normalization ratio: actin intensity parental 231 cells (control lane)/actin intensity POI (POI lane)															

**actin**	1	8721	8461	8199	7961	7578	8010	8759	8526	1.00	0.97	0.94	0.91	0.87	0.92	1.00	0.98								
	2	30401	30934	31270	30264	30252	30630	31179	30883	1.00	1.02	1.03	1.00	0.99	1.01	1.03	1.02								
	3	22075	23709	22976	21257	23186	24316	23592	24347	1.00	1.07	1.04	0.96	1.05	1.10	1.07	1.10								
**RhoA**	1	9098	9121	9991	9428	11782	17143	18033	20715	9098	8938	10654	9957	11322	18458	19496	21579	1.00	0.98	1.17	1.09	1.24	2.03	2.14	2.37
(22 kDa)	2	27994	27864	20396	19276	23908	51947	36554	62745	27994	26661	18330	21297	23229	51289	34499	59368	1.00	0.95	0.65	0.76	0.83	1.83	1.23	2.12
	3	49387	49815	54189	51320	50000	89528	75765	86019	49387	50014	45900	51251	47901	81151	73405	85331	1.00	1.01	0.93	1.04	0.97	1.64	1.49	1.73
	Mean									28826	28538	24961	27502	27484	50299	42467	55426	**1.00**	**0.98**	**0.92**	**0.96**	**1.01**	**1.83**	**1.62**	**2.07**
	SD									20157	20602	18535	21335	18657	31358	27824	32058		0.03	0.26	0.18	0.21	0.19	0.47	0.32
										Lane normalization ratio: actin intensity parental 231 cells (control lane)/actin intensity POI (POI lane)															
**actin**	1	6821	6960	6396	6458	7098	6335	6309	6548	1.00	1.02	0.94	0.95	1.04	0.93	0.92	0.96								
	2	6821	6960	6396	6458	7098	6335	6309	6548	1.00	1.05	1.11	0.91	1.03	1.01	1.06	1.06								
	3	42671	42501	50377	42729	44541	47076	44043	43015	1.00	1.00	1.18	1.00	1.04	1.10	1.03	1.01								
**p-Src (419)**	1	730	672	698	673	673	847	1535	1652	730	657	649	686	669	820	1597	1622	1.00	0.90	0.89	0.94	0.92	1.12	2.19	2.22
(60 kDa)	2	1880	1677	2722	2605	2696	2194	3560	5553	1880	1888	2782	2763	2705	2241	3503	5729	1.00	1.00	1.48	1.47	1.44	1.19	1.86	3.05
	3	7795	5675	6073	6381	6099	6389	12271	9672	7795	5509	6140	6311	5955	6402	12393	10048	1.00	0.71	0.79	0.81	0.76	0.82	1.59	1.29
	Mean									3468	2684	3190	3253	3110	3154	5831	5800	**1.00**	**0.87**	**1.05**	**1.07**	**1.04**	**1.05**	**1.88**	**2.19**
	SD									3791	2522	2768	2845	2667	2901	5762	4213		0.15	0.37	0.35	0.35	0.20	0.30	0.88
										Lane normalization ratio: actin intensity parental 231 cells (control lane)/actin intensity POI (POI lane)															
**actin**	1	6722	6880	7230	6597	6767	6942	6461	6844	1.00	1.02	1.08	0.98	1.01	1.03	0.96	1.02								
	2	13695	12164	13400	12914	13648	13409	13917	13273	1.00	0.89	0.98	0.94	1.00	0.98	1.02	0.97								

	3	28187	29037	27877	28498	28866	28131	27910	27131	1.00	1.03	0.99	1.01	1.02	1.00	0.99	0.96								
**p-Src (527)** (60 kDa)	1	1975	1965	1970	2154	2146	1790	2055	1609	1975	1926	2101	2275	2062	1927	2222	1676	1.00	0.98	1.06	1.15	1.04	0.98	1.12	0.85
	2	6576	4285	4312	4690	4768	5916	6716	3664	6576	4100	3875	5182	4633	5841	6338	3467	1.00	0.62	0.59	0.79	0.70	0.89	0.96	0.53
	3	13485	13952	14355	15169	14980	10760	11931	10257	13485	14008	12159	15149	14351	9753	11559	10175	1.00	1.04	0.90	1.12	1.06	0.72	0.86	0.75
	Mean									7345	6678	6045	7535	7015	5841	6706	5106	**1.00**	**0.88**	**0.85**	**1.02**	**0.94**	**0.86**	**0.98**	**0.71**
	SD									5793	6440	5369	6752	6482	3913	4680	4480		0.22	0.24	0.20	0.20	0.13	0.13	0.17
										Lane normalization ratio: actin intensity parental 231 cells (control lane)/actin intensity POI (POI lane)															
**actin**	1	6821	6960	6396	6458	7098	6335	6309	6548	1.00	1.02	0.94	0.95	1.04	0.93	0.92	0.96								
	2	6821	6960	6396	6458	7098	6335	6309	6548	1.00	1.05	1.11	0.91	1.03	1.01	1.06	1.06								
	3	42671	42501	50377	42729	44541	47076	44043	43015	1.00	1.00	1.18	1.00	1.04	1.10	1.03	1.01								
**Src** (60 kDa)	1	930	934	935	1161	1174	1169	1464	1460	930	963	995	1272	1351	1273	1458	1493	1.00	1.04	1.07	1.37	1.45	1.37	1.57	1.61
	2	2314	3392	3547	4068	2702	4196	3725	4983	2314	3332	3446	4087	2716	4164	3630	4903	1.00	1.44	1.49	1.77	1.17	1.80	1.57	2.12
	3	6628	9203	7013	7581	9874	8398	9334	11110	6628	8569	6738	7873	9401	7624	8734	10073	1.00	1.29	1.02	1.19	1.42	1.15	1.32	1.52
	Mean									3291	4288	3726	4410	4489	4354	4607	5490	**1.00**	**1.26**	**1.19**	**1.44**	**1.35**	**1.44**	**1.48**	**1.75**
	SD									2972	3892	2882	3312	4308	3180	3735	4320		0.20	0.26	0.30	0.15	0.33	0.14	0.32
										Lane normalization ratio: actin intensity parental 231 cells (control lane)/actin intensity POI (POI lane)															
**actin**	1	8721	8461	8199	7961	7578	8010	8759	8526	1.00	0.97	0.94	0.91	0.87	0.92	1.00	0.98								
	2	30401	30934	31270	30264	30252	30630	31179	30883	1.00	1.02	1.03	1.00	0.99	1.01	1.03	1.02								
	3	22075	23709	22976	21257	23186	24316	23592	24347	1.00	1.07	1.04	0.96	1.05	1.10	1.07	1.10								
**p-FAK**	1	622	570	599	525	489	989	1077	1174	622	559	639	554	470	1065	1164	1223	1.00	0.90	1.03	0.89	0.76	1.71	1.87	1.97

(125 kDa)	2	2497	2447	2435	1528	2111	3544	2631	2713	2497	2341	2188	1688	2051	3499	2483	2567	1.00	0.94	0.88	0.68	0.82	1.40	0.99	1.03
	3	5642	6219	5498	5391	7064	9552	9769	9608	5642	6244	4657	5384	6767	8658	9465	9531	1.00	1.11	0.83	0.95	1.20	1.53	1.68	1.69
	Mean									2920	3048	2495	2542	3096	4407	4371	4440	**1.00**	**0.98**	**0.91**	**0.84**	**0.93**	**1.55**	**1.51**	**1.56**
	SD									2537	2908	2027	2525	3276	3877	4461	4460		0.11	0.10	0.15	0.24	0.16	0.46	0.48
										Lane normalization ratio: actin intensity parental 231 cells (control lane)/actin intensity POI (POI lane)															
**actin**	1	6821	6960	6396	6458	7098	6335	6309	6548	1.00	1.02	0.94	0.95	1.04	0.93	0.92	0.96								
	2	6821	6960	6396	6458	7098	6335	6309	6548	1.00	1.05	1.11	0.91	1.03	1.01	1.06	1.06								
	3	42671	42501	50377	42729	44541	47076	44043	43015	1.00	1.00	1.18	1.00	1.04	1.10	1.03	1.01								
**FAK**	1	8151	8945	9741	10924	10549	7465	8948	10348	8151	9182	10392	10880	10889	8104	9685	10700	1.00	1.13	1.27	1.33	1.34	0.99	1.19	1.31
(125 kDa)	2	25108	27504	29775	20269	29382	22896	27455	21118	25108	29676	27880	18940	32158	20557	28575	22277	1.00	1.18	1.11	0.75	1.28	0.82	1.14	0.89
	3	44962	39163	41852	34858	50541	41411	48268	50861	44962	38402	42832	34741	56204	43115	50233	50993	1.00	0.85	0.95	0.77	1.25	0.96	1.12	1.13
	Mean									26074	25753	27035	21521	33084	23925	29498	27990	**1.00**	**1.05**	**1.11**	**0.95**	**1.29**	**0.92**	**1.15**	**1.11**
	SD									18424	15000	16236	12138	22672	17747	20290	20745		0.18	0.16	0.33	0.04	0.09	0.04	0.21
										Lane normalization ratio: actin intensity parental 231 cells (control lane)/actin intensity POI (POI lane)															
**actin**	1	5704	5557	5346	5726	5525	5254	5270	5516	1.00	0.97	0.94	1.00	0.97	0.92	0.92	0.97								
	2	10615	9838	11336	11360	9699	11823	10199	10063	1.00	0.93	1.07	1.07	0.91	1.11	0.96	0.95								
	3	26701	27230	26090	26791	24011	25646	25656	26632	1.00	1.02	0.98	1.00	0.90	0.96	0.96	1.00								
**MMP-9**	1	1240	1566	1249	1573	1400	1682	1465	2112	1240	1607	1333	1567	1445	1826	1586	2184	1.00	1.30	1.07	1.26	1.17	1.47	1.28	1.76
(95 kDa)	2	2854	4431	4234	3572	4932	5764	4892	4561	2854	4781	3965	3338	5398	5175	5092	4811	1.00	1.68	1.39	1.17	1.89	1.81	1.78	1.69
	3	8344	7021	8390	12404	9328	6017	8890	11238	8344	6885	8586	12362	10373	6265	9252	11267	1.00	0.83	1.03	1.48	1.24	0.75	1.11	1.35

	Mean									4146	4424	4628	5756	5739	4422	5310	6087	**1.00**	**1.27**	**1.16**	**1.30**	**1.43**	**1.35**	**1.39**	**1.60**
	SD									3724	2657	3672	5790	4474	2313	3838	4674		0.43	0.20	0.16	0.40	0.54	0.35	0.22
										Lane normalization ratio: actin intensity parental 231 cells (control lane)/actin intensity POI (POI lane)															
**actin**	1	5704	5557	5346	5726	5525	5254	5270	5516	1.00	0.97	0.94	1.00	0.97	0.92	0.92	0.97								
	2	10615	9838	11336	11360	9699	11823	10199	10063	1.00	0.93	1.07	1.07	0.91	1.11	0.96	0.95								
	3	26701	27230	26090	26791	24011	25646	25656	26632	1.00	1.02	0.98	1.00	0.90	0.96	0.96	1.00								
**Ezrin**	1	1326	1437	1670	3465	3405	3266	3773	3881	1326	1460	1706	3727	3602	3555	3778	4212	0.59	0.65	0.76	1.65	1.60	1.58	1.67	1.87
(69 kDa)	2	4689	4811	3662	7273	7278	10324	7920	8211	4689	4967	3799	7287	7221	10110	8154	8538	0.97	1.02	0.78	1.50	1.49	2.08	1.68	1.76
	3	8411	10863	11989	16617	17396	16621	17948	24096	8411	11106	12097	17822	18128	16242	18458	24804	0.66	0.87	0.95	1.40	1.43	1.28	1.45	1.95
	Mean									4809	5844	5867	9612	9650	9969	10130	12518	**0.74**	**0.85**	**0.83**	**1.52**	**1.50**	**1.65**	**1.60**	**1.86**
	SD									3544	4883	5495	7330	7561	6345	7537	10858		0.19	0.11	0.13	0.09	0.41	0.13	0.10
										Lane normalization ratio: actin intensity parental 231 cells (control lane)/actin intensity POI (POI lane)															
**actin**	1	7927	7804	7758	7369	7494	7283	7917	7304	1.00	0.98	0.98	0.93	0.95	0.92	1.00	0.92								
	2	20903	20535	20481	20880	20996	21151	20566	20454	1.00	0.97	0.96	1.00	1.01	1.02	0.97	0.96								
	3	16100	15747	15956	15011	15450	16476	15655	15640	1.00	0.98	0.99	0.93	0.96	1.02	0.97	0.97								
**CD44**	1	10812	10427	10935	16132	15944	10251	19327	20395	10812	10747	11631	17673	18350	11161	19244	20862	1.00	0.99	1.08	1.63	1.70	1.03	1.78	1.93
(80 kDa)	2	22081	21260	33493	21079	30456	22917	28509	41284	22081	20885	32541	21176	30609	22741	27781	40625	1.00	0.95	1.47	0.96	1.39	1.03	1.26	1.84
	3	58673	44859	47394	68130	80560	80592	90851	105776	58673	41767	45535	70752	76700	73164	85009	95907	1.00	0.71	0.78	1.21	1.31	1.25	1.45	1.63
	Mean									30522	24467	29902	36534	41886	35689	44011	52465	**1.00**	**0.88**	**1.11**	**1.27**	**1.46**	**1.10**	**1.50**	**1.80**
	SD									25022	15817	17105	29686	30766	32967	35761	38898		0.15	0.35	0.34	0.21	0.12	0.26	0.15
										Lane normalization ratio:															

										actin intensity parental 231 cells (control lane)/actin intensity POI (POI lane)							
**actin**	1	8721	8461	8199	7961	7578	8010	8759	8526	1.00	0.97	0.94	0.91	0.87	0.92	1.00	0.98
	2	30401	30934	31270	30264	30252	30630	31179	30883	1.00	1.02	1.03	1.00	0.99	1.01	1.03	1.02
	3	22075	23709	22976	21257	23186	24316	23592	24347	1.00	1.07	1.04	0.96	1.05	1.10	1.07	1.10

Table S10. Densitometry analysis comparing the changes in protein expression of metastasis-associated protein markers after shRNA-mediated knockdown of FABP4, FABP5, CYP2C19 in highly lung-seeking TNBC subclone (LM6) (**Figure 2c**). Densitometry software (ImageJ) was used to determine the band intensities for each protein of interest (POI). Band intensities of the corresponding housekeeping protein (actin) were used as loading control (LC) for each lane. Normalized protein intensity for each POI was calculated by multiplying the band intensity by the loading control normalization ratio (ratio of actin intensity in the parental 231 control lane to the actin intensity in the POI lane). Relative protein expression for each POI was calculated as fold change relative to the normalized protein intensity in the parental 231 cells. Mean relative protein expressions and standard deviations for three independent western blotting experiments were obtained and statistical analysis was performed using one-way ANOVA, *post hoc* Dunnett's test, with protein expression of the parental 231 cells as control. Red color indicates $p < 0.05$ (< control).

**Protein/Experiment**		**Protein/Band Densitometry Intensity From ImageJ**								**Normalized Protein Intensity (POI Intensity*Lane Normalization Ratio)**								**Relative Protein Expression (Fold Change Relative to Expression in 231 Cells)**							
		**231**	**iR2L**	**LM1**	**LM2**	**LM3**	**LM4**	**LM5**	**LM6**	**231**	**iR2L**	**LM1**	**LM2**	**LM3**	**LM4**	**LM5**	**LM6**	**231**	**iR2L**	**LM1**	**LM2**	**LM3**	**LM4**	**LM5**	**LM6**
**CYP2C19**	1	6555	6508	7188	2089	2723	5915	5953	2087	6555	6007	6306	2259	2662	6532	5512	1898	1.00	0.92	0.96	0.34	0.41	1.00	0.84	0.29
(54 kDa)	2	29573	24036	33736	11423	13897	24466	24370	7885	29573	24732	31666	11797	14632	25361	24565	7502	1.00	0.84	1.07	0.40	0.49	0.86	0.83	0.25
	3	4829	4177	6454	2217	2181	3694	3874	1345	4829	4908	5995	2015	2028	3453	3204	1347	1.00	1.02	1.24	0.42	0.42	0.72	0.66	0.28
	Mean									13652	11882	14656	5357	6440	11782	11093	3582	**1.00**	**0.92**	**1.09**	**0.39**	**0.44**	**0.86**	**0.78**	**0.27**
	SD									13815	11142	14732	5578	7101	11860	11723	3406		0.09	0.14	0.04	0.05	0.14	0.10	0.02
										Lane normalization ratio: actin intensity parental 231 cells (control lane)/actin intensity POI (POI lane)															
**actin**	1	3369	3650	3840	3115	3447	3051	3639	3706	1.00	1.08	1.14	0.92	1.02	0.91	1.08	1.10								
	2	18977	18443	20217	18375	18023	18307	18826	19946	1.00	0.97	1.07	0.97	0.95	0.96	0.99	1.05								
	3	2424	2063	2610	2667	2608	2593	2931	2421	1.00	0.85	1.08	1.10	1.08	1.07	1.21	1.00								
**FABP4**	1	4683	4689	3785	1979	1991	3839	3740	3770	4683	4485	3308	1660	1696	3730	4047	3545	1.00	0.96	0.71	0.35	0.36	0.80	0.86	0.76
(15 kDa)	2	23012	24205	20036	10536	7316	14474	16562	15439	23012	21397	20089	9716	6766	12569	14579	12966	1.00	0.93	0.87	0.42	0.29	0.55	0.63	0.56
	3	3866	4579	7284	1995	1135	8653	2704	2720	3866	4676	6043	1811	1010	7480	2521	2233	1.00	1.21	1.56	0.47	0.26	1.93	0.65	0.58
	Mean									10520	10186	9813	4395	3157	7926	7049	6248	**1.00**	**1.03**	**1.05**	**0.42**	**0.31**	**1.09**	**0.72**	**0.63**

	SD									10826	9709	9004	4608	3144	4436	6566	5855		0.15	0.45	0.06	0.05	0.74	0.13	0.11
										Lane normalization ratio: actin intensity parental 231 cells (control lane)/actin intensity POI (POI lane)															
**actin**	1	3409	3564	3901	4065	4002	3509	3151	3626	1.00	1.05	1.14	1.19	1.17	1.03	0.92	1.06								
	2	13962	15794	13925	15141	15097	16078	15861	16625	1.00	1.13	1.00	1.08	1.08	1.15	1.14	1.19								
	3	2278	2231	2746	2510	2560	2635	2444	2775	1.00	0.98	1.21	1.10	1.12	1.16	1.07	1.22								
**FABP5**	1	16250	17350	12478	10500	6600	16600	16900	9650	16250	19719	14530	12938	7175	18837	20158	11328	1.00	1.21	0.89	0.80	0.44	1.16	1.24	0.70
(15 kDa)	2	1322	1205	1549	814	882	1405	1381	1254	1322	1135	1700	843	984	1474	1380	1290	1.00	0.86	1.29	0.64	0.74	1.11	1.04	0.98
	3	2160	3330	2390	1650	1770	2170	2680	1770	2160	2838	2052	1551	1568	2149	2085	1640	1.00	1.31	0.95	0.72	0.73	0.99	0.97	0.76
	Mean									6577	7897	6094	5111	3242	7487	7874	4753	**1.00**	**1.13**	**1.04**	**0.72**	**0.64**	**1.09**	**1.08**	**0.81**
	SD									8387	10273	7308	6788	3418	9836	10644	5697		0.24	0.21	0.08	0.17	0.08	0.14	0.15
										Lane normalization ratio: actin intensity parental 231 cells (control lane)/actin intensity POI (POI lane)															
**actin**	1	4855	4272	4169	3940	4466	4278	4070	4136	1.00	0.88	0.86	0.81	0.92	0.88	0.84	0.85								
	2	13871	14728	12640	13387	12432	13226	13885	13489	1.00	1.06	0.91	0.97	0.90	0.95	1.00	0.97								
	3	6136	7199	7148	6529	6927	6196	7887	6622	1.00	1.17	1.16	1.06	1.13	1.01	1.29	1.08								
**Vimentin**	1	4556	6680	7240	2491	2520	1931	2368	4165	4556	6390	6327	2089	2147	1876	2562	3916	1.00	1.40	1.39	0.46	0.47	0.41	0.56	0.86
(57 kDa)	2	25180	13415	16546	6137	7194	7013	11398	8915	25180	11859	16590	5659	6653	6090	10033	7487	1.00	0.47	0.66	0.22	0.26	0.24	0.40	0.30
	3	4791	2382	4939	1244	5211	1283	4243	1750	4791	2433	4097	1129	4638	1109	3955	1437	1.00	0.51	0.86	0.24	0.97	0.23	0.83	0.30
	Mean									11509	6894	9005	2959	4479	3025	5517	4280	**1.00**	**0.79**	**0.97**	**0.31**	**0.57**	**0.30**	**0.60**	**0.49**
	SD									11840	4733	6663	2387	2257	2682	3973	3042		0.53	0.38	0.13	0.36	0.10	0.22	0.32
										Lane normalization ratio: actin intensity parental 231 cells (control lane)/actin intensity POI (POI lane)															

**actin**	1	3409	3564	3901	4065	4002	3509	3151	3626	1.00	1.05	1.14	1.19	1.17	1.03	0.92	1.06								
	2	13962	15794	13925	15141	15097	16078	15861	16625	1.00	1.13	1.00	1.08	1.08	1.15	1.14	1.19								
	3	2278	2231	2746	2510	2560	2635	2444	2775	1.00	0.98	1.21	1.10	1.12	1.16	1.07	1.22								
**RhoA**	1	9361	9362	9369	7606	9613	9324	4767	6599	9361	9240	9752	7709	9711	9258	4647	6820	1.00	0.99	1.04	0.82	1.04	0.99	0.50	0.73
(22 kDa)	2	34029	34032	37911	24057	46607	38995	24018	12651	34029	39024	42611	22852	43146	37799	23663	12867	1.00	1.15	1.25	0.67	1.27	1.11	0.70	0.38
	3	5244	6497	4574	6349	4910	6028	3999	4175	5244	6569	4808	6588	6297	5225	4125	4498	1.00	1.25	0.92	1.26	1.20	1.00	0.79	0.86
	Mean									16211	18278	19057	12383	19718	17427	10812	8062	**1.00**	**1.13**	**1.07**	**0.92**	**1.17**	**1.03**	**0.66**	**0.65**
	SD									15567	18016	20548	9084	20361	17757	11132	4320		0.13	0.17	0.30	0.12	0.07	0.15	0.25
										Lane normalization ratio: actin intensity parental 231 cells (control lane)/actin intensity POI (POI lane)															
**actin**	1	3951	4003	3796	3898	3911	3979	4053	3823	1.00	1.01	0.96	0.99	0.99	1.01	1.03	0.97								
	2	17253	15046	15350	18163	18637	17799	17512	16963	1.00	0.87	0.89	1.05	1.08	1.03	1.02	0.98								
	3	3395	3358	3230	3272	2647	3917	3291	3151	1.00	0.99	0.95	0.96	0.78	1.15	0.97	0.93								
**p-Src(419)**	1	3620	3555	4455	3365	4695	2500	4720	2800	3620	3509	4637	3411	4743	2482	4601	2894	1.00	0.97	1.28	0.94	1.31	0.69	1.27	0.80
(60 kDa)	2	11769	7743	13164	8613	14394	6675	13266	1836	11769	8879	14796	8181	13325	6470	13070	1867	1.00	0.75	1.26	0.70	1.13	0.55	1.11	0.16
	3	757	963	751	565	843	422	734	568	757	974	789	586	1081	366	757	612	1.00	1.29	1.04	0.77	1.43	0.48	1.00	0.81
	Mean								ss	5382	4454	6741	4059	6383	3106	6143	1791	**1.00**	**1.00**	**1.19**	**0.80**	**1.29**	**0.57**	**1.13**	**0.59**
	SD									5714	4036	7236	3839	6285	3100	6299	1143		0.27	0.13	0.13	0.15	0.10	0.14	0.37
										Lane normalization ratio: actin intensity parental 231 cells (control lane)/actin intensity POI (POI lane)															
**actin**	1	3951	4003	3796	3898	3911	3979	4053	3823	1.00	1.01	0.96	0.99	0.99	1.01	1.03	0.97								
	2	17253	15046	15350	18163	18637	17799	17512	16963	1.00	0.87	0.89	1.05	1.08	1.03	1.02	0.98								

	3	3395	3358	3230	3272	2647	3917	3291	3151	1.00	0.99	0.95	0.96	0.78	1.15	0.97	0.93								
**p-Src(527)**	1	2613	3355	2126	2039	2372	3586	2123	3838	2613	3097	1865	2205	2318	3960	1966	3490	1.00	1.19	0.71	0.84	0.89	1.52	0.75	1.34
(60 kDa)	2	13959	13546	8351	11067	11636	13018	9455	15647	13959	13938	7839	11429	12251	13494	9530	14887	1.00	1.00	0.56	0.82	0.88	0.97	0.68	1.07
	3	2584	2463	1533	2073	2081	2572	1788	2538	2584	2894	1424	1884	1935	2404	1479	2541	1.00	1.12	0.55	0.73	0.75	0.93	0.57	0.98
	Mean									6385	6643	3709	5173	5501	6620	4325	6973	**1.00**	**1.10**	**0.61**	**0.80**	**0.84**	**1.14**	**0.67**	**1.13**
	SD									6559	6319	3583	5421	5849	6004	4515	6870		0.09	0.09	0.06	0.08	0.33	0.09	0.18
										Lane normalization ratio: actin intensity parental 231 cells (control lane)/actin intensity POI (POI lane)															
**actin**	1	3369	3650	3840	3115	3447	3051	3639	3706	1.00	1.08	1.14	0.92	1.02	0.91	1.08	1.10								
	2	18977	18443	20217	18375	18023	18307	18826	19946	1.00	0.97	1.07	0.97	0.95	0.96	0.99	1.05								
	3	2424	2063	2610	2667	2608	2593	2931	2421	1.00	0.85	1.08	1.10	1.08	1.07	1.21	1.00								
**Src**	1	2885	3424	3396	2239	3426	2962	1531	2547	2885	3891	3954	2759	3724	3361	1826	2990	1.00	1.35	1.37	0.96	1.29	1.17	0.63	1.04
(60 kDa)	2	15128	17747	14185	21513	16756	16182	7244	8051	15128	16714	15567	22290	18695	16971	7237	8279	1.00	1.10	1.03	1.47	1.24	1.12	0.48	0.55
	3	2293	3278	2696	3833	3262	3903	1269	1485	2293	2794	2314	3602	2890	3865	987	1376	1.00	1.22	1.01	1.57	1.26	1.69	0.43	0.60
	Mean									6769	7800	7279	9550	8436	8066	3350	4215	**1.00**	**1.22**	**1.14**	**1.33**	**1.26**	**1.32**	**0.51**	**0.73**
	SD									7245	7740	7225	11041	8894	7716	3392	3611		0.12	0.20	0.33	0.03	0.31	0.11	0.27
										Lane normalization ratio: actin intensity parental 231 cells (control lane)/actin intensity POI (POI lane)															
**actin**	1	4855	4272	4169	3940	4466	4278	4070	4136	1.00	0.88	0.86	0.81	0.92	0.88	0.84	0.85								
	2	13871	14728	12640	13387	12432	13226	13885	13489	1.00	1.06	0.91	0.97	0.90	0.95	1.00	0.97								
	3	6136	7199	7148	6529	6927	6196	7887	6622	1.00	1.17	1.16	1.06	1.13	1.01	1.29	1.08								

**p-FAK**	1	1078	1036	1038	896	913	895	714	618	1078	1023	1080	908	922	889	696	639	1.00	0.95	1.00	0.84	0.86	0.82	0.65	0.59
(125 kDa)	2	5014	5693	4125	3721	4201	3785	3299	3312	5014	6528	4636	3535	3889	3669	3250	3369	1.00	1.30	0.92	0.70	0.78	0.73	0.65	0.67
	3	881	957	662	566	802	654	655	519	881	968	696	587	1029	567	676	559	1.00	1.10	0.79	0.67	1.17	0.64	0.77	0.63
	Mean									2324	2839	2138	1677	1947	1708	1541	1522	**1.00**	**1.12**	**0.91**	**0.74**	**0.93**	**0.73**	**0.69**	**0.63**
	SD									2331	3195	2173	1617	1683	1706	1481	1600		0.18	0.11	0.09	0.21	0.09	0.07	0.04
										Lane normalization ratio: actin intensity parental 231 cells (control lane)/actin intensity POI (POI lane)															
**actin**	1	3951	4003	3796	3898	3911	3979	4053	3823	1.00	1.01	0.96	0.99	0.99	1.01	1.03	0.97								
	2	17253	15046	15350	18163	18637	17799	17512	16963	1.00	0.87	0.89	1.05	1.08	1.03	1.02	0.98								
	3	3395	3358	3230	3272	2647	3917	3291	3151	1.00	0.99	0.95	0.96	0.78	1.15	0.97	0.93								
**FAK**	1	4556	4114	4510	3240	4080	4571	5873	5430	4556	3797	3957	3504	3988	5048	5438	4937	1.00	0.83	0.87	0.77	0.88	1.11	1.19	1.08
(6125kDa)	2	17717	20784	20783	13323	16184	23996	25051	20997	17717	21385	19508	13759	17040	24874	25251	19977	1.00	1.21	1.10	0.78	0.96	1.40	1.43	1.13
	3	2784	3629	3671	2441	1777	4388	3759	4020	2784	4264	3410	2219	1652	4102	3109	4025	1.00	1.53	1.22	0.80	0.59	1.47	1.12	1.45
	Mean									8352	9815	8958	6494	7560	11341	11266	9646	**1.00**	**1.19**	**1.06**	**0.78**	**0.81**	**1.33**	**1.25**	**1.22**
	SD									8158	10023	9140	6325	8293	11729	12167	8958		0.35	0.18	0.01	0.19	0.19	0.16	0.20
										Lane normalization ratio: actin intensity parental 231 cells (control lane)/actin intensity POI (POI lane)															
**actin**	1	3369	3650	3840	3115	3447	3051	3639	3706	1.00	1.08	1.14	0.92	1.02	0.91	1.08	1.10								
	2	18977	18443	20217	18375	18023	18307	18826	19946	1.00	0.97	1.07	0.97	0.95	0.96	0.99	1.05								
	3	2424	2063	2610	2667	2608	2593	2931	2421	1.00	0.85	1.08	1.10	1.08	1.07	1.21	1.00								
**Ezrin**	1	6866	6843	5772	3526	4196	4235	6880	8876	6866	6316	5064	3814	4101	4677	6370	8070	1.00	0.92	0.74	0.56	0.60	0.68	0.93	1.18

	2	31535	27681	25022	15061	21055	21340	25660	32059	31535	28482	23487	15554	22168	22121	25865	30501	1.00	0.90	0.74	0.49	0.70	0.70	0.82	0.97
	3	5335	4451	4194	2850	3445	3824	4011	5665	5335	5229	3896	2590	3203	3575	3318	5672	1.00	0.98	0.73	0.49	0.60	0.67	0.62	1.06
	Mean									14579	13343	10815	7319	9824	10124	11851	14748	**1.00**	**0.93**	**0.74**	**0.51**	**0.63**	**0.68**	**0.79**	**1.07**
	SD									14705	13122	10989	7158	10700	10404	12232	13696		0.04	0.01	0.04	0.06	0.02	0.16	0.10
										Lane normalization ratio: actin intensity parental 231 cells (control lane)/actin intensity POI (POI lane)															
**actin**	1	3369	3650	3840	3115	3447	3051	3639	3706	1.00	1.08	1.14	0.92	1.02	0.91	1.08	1.10								
	2	18977	18443	20217	18375	18023	18307	18826	19946	1.00	0.97	1.07	0.97	0.95	0.96	0.99	1.05								
	3	2424	2063	2610	2667	2608	2593	2931	2421	1.00	0.85	1.08	1.10	1.08	1.07	1.21	1.00								
**CD44**	1	17361	15640	17358	17933	8326	15633	22512	22939	17361	14960	15168	15039	7093	15189	24358	21570	1.00	0.86	0.87	0.87	0.41	0.87	1.40	1.24
	2	75094	84354	79237	79893	22080	86576	120846	108459	75094	74568	79446	73672	20420	75181	106376	91087	1.00	0.99	1.06	0.98	0.27	1.00	1.42	1.21
	3	13825	16376	14346	14091	6325	9329	19717	17981	13825	16724	11901	12792	5630	8065	18379	14760	1.00	1.21	0.86	0.93	0.41	0.58	1.33	1.07
	Mean									35427	35418	35505	33834	11048	32812	49704	42472	**1.00**	**1.02**	**0.93**	**0.92**	**0.36**	**0.82**	**1.38**	**1.17**
	SD									34398	33917	38089	34519	8150	36866	49170	42239		0.18	0.11	0.06	0.08	0.21	0.05	0.09
										Lane normalization ratio: actin intensity parental 231 cells (control lane)/actin intensity POI (POI lane)															
**Actin**	1	3409	3564	3901	4065	4002	3509	3151	3626	1.00	1.05	1.14	1.19	1.17	1.03	0.92	1.06								
	2	13962	15794	13925	15141	15097	16078	15861	16625	1.00	1.13	1.00	1.08	1.08	1.15	1.14	1.19								
	3	2278	2231	2746	2510	2560	2635	2444	2775	1.00	0.98	1.21	1.10	1.12	1.16	1.07	1.22								

Table S11. Densitometry analysis comparing the cellular localization of FABP4 and FABP5, and of lipogenic transcription factors PPAR-γ, SREBP-2, RAR and RXR-α in vehicle-treated or EET-supplemented LM6 cells (**Figure 3e)**. Densitometry software (ImageJ) was used to determine the band intensities for each protein of interest (POI). Band intensities of the corresponding housekeeping protein, GADPH (loading control for whole cell lysate and cytosolic fractions) and lamin (loading control for nuclear fraction), were used as loading control (LC) for each lane. Normalized protein intensity for each POI was calculated by multiplying the band intensity by the loading control normalization ratio (ratio of GADPH/lamin intensity in the vehicle-treated control lane to the GADPH/lamin intensity in the POI lane. Relative protein expression for each POI was calculated as fold change relative to the normalized protein intensity of vehicle-treated cells. Mean relative protein expressions and standard deviations for three independent western blotting experiments were obtained and statistical analysis was performed using student's t-test, with protein expression of corresponding vehicle-treated cells as control. Red color indicates $p < 0.05$.

Protein/Experiment		Protein/Band Densitometry Intensity from ImageJ						Normalized Protein Intensity (POI Intensity*Lane Normalization Ratio)						Relative Protein Expression (Fold Change Relative to Expression in Vehicle Control)					
		**WCL (vehicle)**	**WCL (+EET)**	**Nuclear (vehicle)**	**Nuclear (+EET)**	**Cytosolic (vehicle)**	**Cytosolic (+EET)**	**WCL (-)**	**WCL (+)**	**N (-)**	**N (+)**	**C (-)**	**C (+)**	**WCL (-)**	**WCL (+)**	**N (-)**	**N (+)**	**C (-)**	**C (+)**
CYP2C19	1	18978	17096	17045	20726	17808	17129	18978	16673	17370	21015	19237	17130	1.00	0.88	0.92	1.11	1.01	0.90
(54 kDa)	2	4615	4730	3279	3706	4631	4302	4615	3354	2299	2601	4148	3657	1.00	0.73	1.00	1.13	1.00	0.88
	3	51363	44466	35345	56369	39378	37276	51363	47521	34529	59505	39078	39385	1.00	0.93	1.00	1.72	1.00	1.01
	Mean							24986	22516	18066	27707	0	0	1.00	0.84	0.97	1.32	1.00	0.93
	SD							23946	22656	16126	29036	17519	0		0.10	0.05	0.35	0.01	0.07
								Lane normalization ratio: GADPH/lamin intensity vehicle-treated cells (control lane)/ GADPH/lamin intensity POI (POI lane)											
GADPH	1	9165	8782	0	0	8484	9164	1.00	0.96	0.00	0.00	0.93	1.00						
(37 kDa)	2	3141	3006	0	0	3507	3696	1.00	0.96	0.00	0.00	1.12	1.18						
	3	26649	27759	0	0	26853	25222	1.00	1.04	0.00	0.00	1.01	0.95						
								Lane normalization ratio: GADPH/lamin intensity vehicle-treated cells (control lane)/ GADPH/lamin intensity POI (POI lane)											

Lamin a/c	1	12538	12856	12303	12365	0	0	1.00	1.03	0.98	0.99	0.00	0.00						
(69/62 kDa)	2	2027	2859	2892	2888	0	0	1.00	1.41	1.43	1.42	0.00	0.00						
	3	5489	5136	5618	5199	0	0	1.00	0.94	1.02	0.95	0.00	0.00						
FABP4	1	1227	1560	1329	2605	2253	2049	1227	1628	1354	2641	2434	2049	1.00	1.33	1.00	1.95	1.00	0.84
(15 kDa)	2	276	520	279	497	604	767	276	543	195	349	541	652	1.00	1.96	1.00	1.78	1.00	1.20
	3	2966	5033	3698	4402	4689	4365	2966	4832	3612	4647	4653	4612	1.00	1.63	1.00	1.29	1.00	0.99
	Mean							1490	2334	1720	2546	2543	2438	1.00	1.64	1.00	1.67	1.00	1.01
	SD							1364	2230	1738	2151	2058	2009		0.32	0.00	0.35	0.00	0.18
								Lane normalization ratio: GADPH/lamin intensity vehicle-treated cells (control lane)/ GADPH/lamin intensity POI (POI lane)											
GADPH	1	9165	8782	0	0	8484	9164	1.00	0.96	0.00	0.00	0.93	1.00						
(37 kDa)	2	3141	3006	0	0	3507	3696	1.00	0.96	0.00	0.00	1.12	1.18						
	3	26649	27759	0	0	26853	25222	1.00	1.04	0.00	0.00	1.01	0.95						
Lamin a/c	1	12538	12856	12303	12365	0	0	1.00	1.03	0.98	0.99	0.00	0.00						
(69/62 kDa)	2	2027	2859	2892	2888	0	0	1.00	1.41	1.43	1.42	0.00	0.00						
	3	5489	5136	5618	5199	0	0	1.00	0.94	1.02	0.95	0.00	0.00						
FABP5	1	2461	4349	1874	2732	2119	1862	2461	4467	1850	2689	2158	1850	1.00	1.82	0.75	1.09	0.88	0.75
(15 kDa)	2	568	982	369	454	673	648	568	939	359	464	646	644	1.00	1.65	0.63	0.82	1.14	1.13
	3	6431	8913	4274	8576	8571	5857	6431	10124	4514	9142	9015	5850	1.00	1.57	0.70	1.42	1.40	0.91
	Me							3153	5177	2241	4098	3940	2781	1.00	1.68	0.70	1.11	1.14	0.93

	an SD							2992	4633	2105	4507	4460	2725		0.12	0.06	0.30	0.26	0.19
								Lane normalization ratio: GADPH/lamin intensity vehicle-treated cells (control lane)/ GADPH/lamin intensity POI (POI lane)											
GADPH	1	7624	7422	0	0	7485	7676	1.00	0.97	0.00	0.00	0.98	1.01						
(37 kDa)	2	1684	1762	0	0	1753	1846	1.00	1.05	0.00	0.00	1.04	1.10						
	3	21849	19235	0	0	20773	21872	1.00	0.88	0.00	0.00	0.95	1.00						
Lamin a/c	1	8237	8131	8343	8372	0	0	1.00	0.99	1.01	1.02	0.00	0.00						
	2	1815	2154	1866	1775	0	0	1.00	1.19	1.03	0.98	0.00	0.00						
	3	2106	2047	1994	1975	0	0	1.00	0.97	0.95	0.94	0.00	0.00						
PPAR-γ	1	11854	15344	11824	31432	0	0	11854	14964	12050	31871	0	0	1.00	1.26	1.02	2.69	0.00	0.00
(57 kDa)	2	2951	4219	2457	5863	0	0	2951	2991	1722	4115	0	0	1.00	1.01	0.58	1.39	0.00	0.00
	3	31136	41958	28432	66655	0	0	31136	44840	27775	70364	0	0	1.00	1.44	0.89	2.26	0.00	0.00
	Mean							15314	20932	13849	35450	0	0	1.00	1.24	0.83	2.11	0.00	0.00
	SD							14407	21553	13119	33269	0	0		0.21	0.22	0.66	0.00	0.00
								Lane normalization ratio: GADPH/lamin intensity vehicle-treated cells (control lane)/ GADPH/lamin intensity POI (POI lane)											
GADPH	1	9165	8782	0	0	8484	9164	1.00	0.96	0.00	0.00	0.93	1.00						
	2	3141	3006	0	0	3507	3696	1.00	0.96	0.00	0.00	1.12	1.18						
	3	26649	27759	0	0	26853	25222	1.00	1.04	0.00	0.00	1.01	0.95						

Lamin a/c	1	12538	12856	12303	12365	0	0	1.00	1.03	0.98	0.99	0.00	0.00						
(69/62 kDa)	2	2427	2859	2892	2888	0	0	1.20	1.41	1.43	1.42	0.00	0.00						
	3	5489	5136	5618	5199	0	0	1.00	0.94	1.02	0.95	0.00	0.00						
SREBP-2	1	1057	1776	1296	1618	0	0	1057	1732	1321	1641	0	0	1.00	1.64	1.00	1.24	0.00	0.00
(60 kDa)	2	722	685	242	412	0	0	722	581	203	346	0	0	1.00	0.80	1.00	1.70	0.00	0.00
	3	7591	9233	9582	15450	0	0	7591	9867	9360	16310	0	0	1.00	1.30	1.00	1.74	0.00	0.00
	Mean							3124	4060	3628	6099	0	0	1.00	1.25	**1.00**	**1.56**	**0.00**	**0.00**
	SD							3873	5062	4995	8867	0	0		0.42	0.00	0.28	0.00	0.00
								Lane normalization ratio: GADPH/lamin intensity vehicle-treated cells (control lane)/ GADPH/lamin intensity POI (POI lane)											
GADPH	1	9165	8782	0	0	8484	9164	1.00	0.96	0.00	0.00	0.93	1.00						
(37 kDa)	2	3141	3006	0	0	3507	3696	1.00	0.96	0.00	0.00	1.12	1.18						
	3	26649	27759	0	0	26853	25222	1.00	1.04	0.00	0.00	1.01	0.95						
Lamin a/c	1	12538	12856	12303	12365	0	0	1.00	1.03	0.98	0.99	0.00	0.00						
(69/62 kDa)	2	2427	2859	2892	2888	0	0	1.00	1.18	1.19	1.19	0.00	0.00						
	3	5489	5136	5618	5199	0	0	1.00	0.94	1.02	0.95	0.00	0.00						
RXR	1	24508	0	1096	1083	0	0	24508	0	1082	1065	0	0	1.00	0.00	1.00	0.98	0.00	0.00
(51 kDa)	2	4692	0	5616	2291	0	0	4692	0	5464	2343	0	0	1.00	0.00	1.00	0.43	0.00	0.00
	3	68190	0	59180	33557	0	0	68190	0	62509	35772	0	0	1.00	0.00	1.00	0.57	0.00	0.00
	Me							32463	0	23018	13060	0	0	1.00	0.00	**1.00**	**0.66**	**0.00**	**0.00**

	anSD							32488	0	34270	19680	0	0		0.00	0.00	0.29	0.00	0.00
								Lane normalization ratio: GADPH/lamin intensity vehicle-treated cells (control lane)/ GADPH/lamin intensity POI (POI lane)											
GADPH	1	7624	7422	0	0	7485	7676	1.00	0.97	0.00	0.00	0.98	1.01						
(37 kDa)	2	1684	1762	0	0	1753	1846	1.00	1.05	0.00	0.00	1.04	1.10						
	3	21849	19235	0	0	20773	21872	1.00	0.88	0.00	0.00	0.95	1.00						
Lamin a/c	1	8237	8131	8343	8372	0	0	1.00	0.99	1.01	1.02	0.00	0.00						
(69/62 kDa)	2	1815	2154	1866	1775	0	0	1.00	1.19	1.03	0.98	0.00	0.00						
	3	2106	2047	1994	1975	0	0	1.00	0.97	0.95	0.94	0.00	0.00						
RAR	1	2940	2395	1096	1083	2928	1118	2940	2426	1082	1065	2982	1110	1.00	0.83	0.37	0.36	1.01	0.38
(51 kDa)	2	806	528	221	268	785	202	806	445	215	274	754	201	1.00	0.55	0.27	0.34	0.93	0.25
	3	7164	6690	2775	2572	8073	2644	7164	6882	2931	2741	8491	2642	1.00	0.96	0.41	0.38	1.19	0.37
	Mean							3637	3251	1409	1360	4076	1318	1.00	0.78	**0.35**	**0.36**	**1.04**	**0.33**
	SD							3236	3297	1387	1260	3983	1233		0.21	0.07	0.02	0.13	0.07
								Lane normalization ratio: GADPH/lamin intensity vehicle-treated cells (control lane)/ GADPH/lamin intensity POI (POI lane)											
GADPH	1	7624	7422	0	0	7485	7676	1.00	0.97	0.00	0.00	0.98	1.01						
(37 kDa)	2	1684	1762	0	0	1753	1846	1.00	1.05	0.00	0.00	1.04	1.10						
	3	21849	19235	0	0	20773	21872	1.00	0.88	0.00	0.00	0.95	1.00						

Lamin a/c	1	823<br>7	8131	8343	8372	0	0	1.00	0.99	1.01	1.02	0.00	0.00
(69/62<br>kDa)	2	181<br>5	2154	1866	1775	0	0	1.00	1.19	1.03	0.98	0.00	0.00
	3	210<br>6	2047	1994	1975	0	0	1.00	0.97	0.95	0.94	0.00	0.00

Table S12. Densitometry analysis comparing the expression of downstream direct targets of PPAR-γ and SREBP-2 in LM6-LacZ, LM6-shFABP4 and LM6-shFABP5 cells grown with or without EET supplementation (Figure 3f). Densitometry software (ImageJ) was used to determine the band intensities for each protein of interest (POI). Band intensities of the corresponding housekeeping protein (actin) were used as loading control (LC) for each lane. Normalized protein intensity for each POI was calculated by multiplying the band intensity by the loading control normalization ratio (ratio of actin intensity in the parental LM6-shLacZ cells control lane to the actin intensity in the POI lane). Relative protein expression for each POI was calculated as fold change relative to the normalized protein intensity in the LM6-shLacZ cells. Mean relative protein expressions and standard deviations for three independent western blotting experiments were obtained and statistical analysis was performed using one-way ANOVA, *post hoc* Dunnett's test, with protein expression of the LM6-shLacZ cells as control. Red color indicates $p < 0.05$.

Protein/Experiment		**Protein/Band Densitometry Intensity from ImageJ**						**Normalized Protein Intensity (POI Intensity*Lane Normalization Ratio)**						**Relative Protein Expression (Fold Change Relative to Expression in 231 Cells)**					
		shLacZ (-)	shLacZ (+EETs)	shFABP4 (-)	shFABP4 (+EETs)	shFABP5 (-)	shFABP5 (+EETs)	shLacZ (-)	shLacZ (+EETs)	shFABP4 (-)	shFABP4 (+EETs)	shFABP5 (-)	shFABP5 (+EETs)	shLacZ (-)	shLacZ (+EETs)	shFABP4 (-)	shFABP4 (+EETs)	shFABP5 (-)	shFABP5 (+EETs)
CD36 (85 kDa)	1	1775	3503	1044	1285	1423	1439	1775	3511	1049	1289	1437	1437	1.00	1.98	0.59	0.73	0.81	0.81
	2	4796	9530	4399	3824	4106	3209	4796	10274	4651	3782	4143	3421	1.00	2.14	0.97	0.79	0.86	0.71
	3	27207	36043	24996	17049	22300	14257	27207	36327	25597	17170	22432	14020	1.00	1.34	0.94	0.63	0.82	0.52
	Mean							11259	16704	10432	7414	9337	6293	1.00	1.82	0.83	0.72	0.83	0.68
	SD							13893	17327	13256	8541	11420	6765		0.43	0.21	0.08	0.03	0.15
								Lane normalization ratio: actin intensity LM6-shLacZ cells (control)/actin intensity of POI lane)											
actin	1	9504	9481	9463	9473	9411	9519	1.00	1.00	1.00	1.00	0.99	1.00						
	2	15575	14447	14731	15750	15437	14609	1.00	0.93	0.95	1.01	0.99	0.94						
	3	33621	33359	32832	33385	33424	34190	1.00	0.99	0.98	0.99	0.99	1.02						
c-myc (57 kDa)	1	2074	3107	1841	1912	1707	1623	2074	3131	2134	2410	1815	1450	1.00	1.51	1.03	1.16	0.87	0.70
	2	5998	7980	4442	4928	4989	4718	5998	6431	3657	4750	4871	4317	1.00	1.07	0.61	0.79	0.81	0.72
	3	23005	43820	22422	21335	28493	18167	23005	45691	23019	20219	28244	17650	1.00	1.99	1.00	0.88	1.23	0.77
	Mean							10359	18418	9603	9126	11643	7806	1.00	1.52	0.88	0.94	0.97	0.73
	SD							11126	23677	11643	9678	14457	8645		0.46	0.23	0.19	0.22	0.03
								Lane normalization ratio: actin intensity LM6-shLacZ cells (control)/actin intensity of POI lane)											
actin	1	13256	13156	11434	10518	12471	14840	1.00	0.99	0.86	0.79	0.94	1.12						
	2	23181	28765	28158	24048	23743	25332	1.00	1.24	1.21	1.04	1.02	1.09						
	3	6389	6127	6223	6741	6445	6576	1.00	0.96	0.97	1.06	1.01	1.03						
Ezrin (69 kDa)	1	3440	3506	1044	1592	1045	1052	3440	3533	1210	2006	1111	940	1.00	1.03	0.35	0.58	0.32	0.27
	2	9255	9572	2666	5056	2669	3064	9255	7714	2195	4874	2606	2804	1.00	0.83	0.24	0.53	0.28	0.30
	3	44713	60366	11986	27586	11832	13843	44713	62944	12305	26143	11728	13449	1.00	1.41	0.28	0.58	0.26	0.30
	Mean							19136	24730	5237	11008	5148	5731	1.00	1.09	0.29	0.56	0.29	0.29
	SD							22340	33160	6141	13186	5747	6749		0.29	0.06	0.03	0.03	0.02

								Lane normalization ratio: actin intensity LM6-shLacZ cells (control)/actin intensity of POI lane)											
actin	1	13256	13156	11434	10518	12471	14840	1.00	0.99	0.86	0.79	0.94	1.12						
	2	23181	28765	28158	24048	23743	25332	1.00	1.24	1.21	1.04	1.02	1.09						
	3	6389	6127	6223	6741	6445	6576	1.00	0.96	0.97	1.06	1.01	1.03						
CD44 (80 kDa)	1	2804	5096	1120	874	806	0	2804	5108	1125	877	814	0	1.00	1.82	0.40	0.31	0.29	0.00
	2	7841	13233	3242	2422	2170	0	7841	13475	3111	2335	2119	0	1.00	1.72	0.40	0.30	0.27	0.00
	3	37194	75803	19364	15094	12882	0	37194	72487	17551	14696	12784	0	1.00	1.95	0.47	0.40	0.34	0.00
	Mean							15946	30357	7262	5969	5239	0	1.00	1.83	0.42	0.34	0.30	0.00
	SD							18573	36725	8965	7593	6567	0		0.12	0.04	0.05	0.04	0.00
								Lane normalization ratio: actin intensity LM6-shLacZ cells (control)/actin intensity of POI lane)											
actin	1	14440	14405	14378	14393	14299	14463	1.00	1.00	1.00	1.00	0.99	1.00						
	2	23181	22765	24158	24048	23743	24332	1.00	0.98	1.04	1.04	1.02	1.05						
	3	7082	7406	7813	7274	7136	7354	1.00	1.05	1.10	1.03	1.01	1.04						
Src (60 kDa)	1	3962	3901	1834	2000	2545	1611	3962	3910	1842	2007	2570	1608	1.00	0.99	0.46	0.51	0.65	0.41
	2	9830	10579	7044	5929	6083	7251	9830	10772	6759	5715	5939	6908	1.00	1.10	0.69	0.58	0.60	0.70
	3	46802	61978	21655	14435	22355	33277	46802	59267	19627	14054	22186	32045	1.00	1.27	0.42	0.30	0.47	0.68
	Mean							20198	24650	9409	7259	10232	13521	1.00	1.12	0.52	0.46	0.58	0.60
	SD							23226	30175	9184	6170	10489	16260		0.14	0.14	0.15	0.09	0.17
								Lane normalization ratio: actin intensity LM6-shLacZ cells (control)/actin intensity of POI lane)											
actin	1	14440	14405	14378	14393	14299	14463	1.00	1.00	1.00	1.00	0.99	1.00						
	2	23181	22765	24158	24048	23743	24332	1.00	0.98	1.04	1.04	1.02	1.05						
	3	7082	7406	7813	7274	7136	7354	1.00	1.05	1.10	1.03	1.01	1.04						
FAK (125 kDa)	1	2804	5096	1853	441	473	0	2804	5135	2148	556	503	0	1.00	1.83	0.77	0.20	0.18	0.00
	2	7066	13053	8591	1323	1280	0	7066	10519	7073	1275	1250	0	1.00	1.49	1.00	0.18	0.18	0.00
	3	30843	51984	26494	7227	5254	0	30843	54204	27200	6849	5208	0	1.00	1.76	0.88	0.22	0.17	0.00
	Mean							13571	23286	12140	2893	2320	0	1.00	1.69	0.88	0.20	0.18	0.00
	SD							15109	26911	13272	3445	2529	0		0.18	0.12	0.02	0.01	0.00
								Lane normalization ratio: actin intensity LM6-shLacZ cells (control)/actin intensity of POI lane)											
actin	1	13256	13156	11434	10518	12471	14840	1.00	0.99	0.86	0.79	0.94	1.12						
	2	23181	28765	28158	24048	23743	25332	1.00	1.24	1.21	1.04	1.02	1.09						
	3	6389	6127	6223	6741	6445	6576	1.00	0.96	0.97	1.06	1.01	1.03						
PPAR-γ (57 kDa)	1	1635	2654	582	716	538	589	1635	2660	585	718	543	588	1.00	1.63	0.36	0.44	0.33	0.36
	2	4722	5251	2547	2216	3239	1783	4722	5661	2693	2191	3268	1901	1.00	1.20	0.57	0.46	0.69	0.40
	3	26362	41224	4752	19351	10518	15736	26362	41549	4866	19488	10580	15474	1.00	1.58	0.18	0.74	0.40	0.59
	Mean							10906	16623	2715	7466	4797	5988	1.00	1.47	0.37	0.55	0.48	0.45

	SD							13474	21638	2141	10438	5190	8242		0.23	0.19	0.17	0.19	0.12
								Lane normalization ratio: actin intensity LM6-shLacZ cells (control)/actin intensity of POI lane)											
actin	1	9504	9481	9463	9473	9411	9519	1.00	1.00	1.00	1.00	0.99	1.00						
	2	15575	14447	14731	15750	15437	14609	1.00	0.93	0.95	1.01	0.99	0.94						
	3	33621	33359	32832	33385	33424	34190	1.00	0.99	0.98	0.99	0.99	1.02						
SREBP-2	1	2350	2764	1718	1924	932	1220	2350	2771	1725	1930	941	1218	1.00	1.18	0.73	0.82	0.40	0.52
(69 kDa)	2	6113	7626	2230	2481	1778	3704	6113	8222	2358	2453	1794	3949	1.00	1.34	0.39	0.40	0.29	0.65
	3	27194	28433	9993	13625	14542	19741	27194	28657	10233	13722	14628	19413	1.00	1.05	0.38	0.50	0.54	0.71
	Mean							11886	13216	4772	6035	5788	8193	1.00	1.19	0.50	0.58	0.41	0.63
	SD							13390	13647	4740	6662	7668	9812		0.15	0.20	0.22	0.12	0.10
								Lane normalization ratio: actin intensity LM6-shLacZ cells (control)/actin intensity of POI lane)											
actin	1	9504	9481	9463	9473	9411	9519	1.00	1.00	1.00	1.00	0.99	1.00						
	2	15575	14447	14731	15750	15437	14609	1.00	0.93	0.95	1.01	0.99	0.94						
	3	33621	33359	32832	33385	33424	34190	1.00	0.99	0.98	0.99	0.99	1.02						

Table S13. Densitometry analysis comparing the protein expression levels of adipocyte markers and intrinsic expression levels of FABP4, FABP5 and CYP2C19 in fibroblasts, adipocytes and monocytes grown in monoculture, with or without addition of 2 µM rosiglitazone (**Figure 4a).** Densitometry software (ImageJ) was used to determine the band intensities for each protein of interest (POI). Band intensities of the corresponding housekeeping protein (actin) were used as loading control (LC) for each lane. Normalized protein intensity for each POI was calculated by multiplying the band intensity by the loading control normalization ratio (ratio of actin intensity in the vehicle-treated control lane to the actin intensity in the POI lane). Relative protein expression for each POI was calculated as fold change relative to the normalized protein intensity in the vehicle-treated control cells. Mean relative protein expressions and standard deviations for three independent western blotting experiments were obtained and statistical analysis was performed using one-way ANOVA, *post hoc* Dunnett's test, with protein expression of the vehicle-treated cells as control. Red color indicates $p < 0.05$.

**Protein/Experiment**		**Protein/Band Densitometry Intensity from ImageJ**				**Normalized Protein Intensity (POI Intensity*Lane Normalization Ratio)**				**Relative Protein Expression (Fold change relative to expression in 231 cells)**			
		THP-1 (-)	THP-1 (+)	3T3 (-)	3T3 (+)	THP-1 (-)	THP-1 (+)	3T3 (-)	3T3 (+)	THP-1 (-)	THP-1 (+)	3T3 (-)	3T3 (+)
C/EBPα	1	2742	2694	4254	3593	2742	2732	4557	3837	1.00	1.00	1.00	0.84
	2	25949	27054	41510	44030	25949	24865	36426	40527	1.00	0.96	1.00	1.11
	3	5430	6768	17763	12930	5430	6627	16539	12926	1.00	1.22	1.00	0.78
	Mean					11374	11408	19174	19097	1.00	1.06	1.00	0.91
	SD					12694	11816	16097	19107		0.14	0.00	0.18
						Lane normalization ratio: actin intensity parental 231 cells (control)/actin intensity of POI lane)							
Actin	1	11272	11115	10522	10555	1.00	0.99	0.93	0.94				
	2	19233	20926	21917	20896	1.00	1.09	1.14	1.09				
	3	7178	7331	7710	7181	1.00	1.02	1.07	1.00				
PPAR-γ	1	3502	7782	4486	6206	3502	7824	4506	6142	1.00	2.23	1.00	1.36
	2	12762	11352	13974	33957	12762	11727	13817	33608	1.00	0.92	1.00	2.43
	3	4360	8071	9046	17467	4360	7787	8866	17753	1.00	1.79	1.00	2.00

	Mean					6875	9113	9063	19168	1.00	1.65	1.00	1.93
	SD					5116	2264	4659	13787		0.67	0.00	0.54
						Lane normalization ratio: actin intensity parental 231 cells (control)/actin intensity of POI lane)							
Actin	1	6468	6434	6441	6536	1.00	0.99	1.00	1.01				
	2	45706	44243	46224	46180	1.00	0.97	1.01	1.01				
	3	16803	17416	17144	16532	1.00	1.04	1.02	0.98				
FABP4	1	28037	63666	55532	69273	28037	64012	55772	68562	1.00	2.28	1.00	1.23
	2	3466	7031	1843	2725	3466	7221	1826	2702	1.00	2.08	1.00	1.48
	3	1336	1903	1501	2470	1336	1836	1471	2511	1.00	1.37	1.00	1.71
	Mean					10946	24356	19690	24591	1.00	1.91	1.00	1.47
	SD					14839	34448	31249	38079		0.48	0.00	0.24
						Lane normalization ratio: actin intensity parental 231 cells (control)/actin intensity of POI lane)							
Actin	1	6468	6434	6441	6536	1.00	0.99	1.00	1.01				
	2	55706	54243	56224	56180	1.00	0.97	1.01	1.01				
	3	16803	17416	17144	16532	1.00	1.04	1.02	0.98				
FABP5	1	3108	9528	7491	9213	3108	11173	9074	10233	1.00	3.60	1.00	1.13
	2	8964	21938	27612	28974	8964	20028	27572	28067	1.00	2.23	1.00	1.02
	3	8915	29102	23003	33151	8915	30187	23279	32487	1.00	3.39	1.00	1.40
	Mean					6996	20463	19975	23596	1.00	3.07	1.00	1.18
	SD					3367	9514	9682	11782		0.73	0.00	0.19
						Lane normalization ratio: actin intensity parental 231 cells (control)/actin intensity of POI lane)							
Actin	1	8833.39	7532.9	7292.13	7952.788	1.00	0.85	0.83	0.90				
	2	18127.66	19856.63	18153.89	18713.86	1.00	1.10	1.00	1.03				
	3	25116.49	24213.74	24819.52	25629.86	1.00	0.96	0.99	1.02				
CYP2C19	1	4247	5874	4201	4902	4247	6889	5089	5445	1.00	1.62	1.00	1.07
	2	17667	35066	17764	27906	17667	32013	17739	27031	1.00	1.81	1.00	1.52
	3	20080	13538	13175	10906	20080	14043	13333	10688	1.00	0.70	1.00	0.80
	Mean					13998	17648	12053	14388	1.00	1.38	1.00	1.13
	SD					8530	12944	6421	11259		0.60	0.00	0.37
						Lane normalization ratio: actin intensity parental 231 cells (control)/actin intensity of POI lane)							
Actin	1	8833.39	7532.9	7292.13	7952.788	1.00	0.85	0.83	0.90				
	2	18127.66	19856.63	18153.89	18713.86	1.00	1.10	1.00	1.03				
	3	25116.49	24213.74	24819.52	25629.86	1.00	0.96	0.99	1.02				

Table S14. Densitometry analysis comparing the expression of FABP4, FABP4 and CYP2C19 in parental 231, LM6-shLacZ, LM6-shFABP4, LM6-shFABP5 and LM6-CYP2C19-depleted cells (Figure 4b; cells grown in monoculture). Densitometry software (ImageJ) was used to determine the band intensities for each protein of interest (POI). Band intensities of the corresponding housekeeping protein (actin) were used as loading control (LC) for each lane. Normalized protein intensity for each POI was calculated by multiplying the band intensity by the loading control normalization ratio (ratio of actin intensity in the parental 231 control lane to the actin intensity in the POI lane). Relative protein expression for each POI was calculated as fold change relative to the normalized protein intensity in the parental 231 cells. Mean relative protein expressions and standard deviations for three independent western blotting experiments were obtained and statistical analysis was performed using one-way ANOVA, *post hoc* Dunnett's test, with protein expression of the parental 231 cells as control. Red color indicates $p < 0.05$.

**Protein/Experiment**		**Protein/Band Densitometry Intensity from ImageJ**					**Normalized Protein Intensity (POI Intensity*Lane Normalization Ratio)**					**Relative protein expression (Fold Change Relative to Expression in 231 Cells)**				
		231	LM6-shLacZ	LM6-shFABP4	LM6-shFABP5	LM6-shCYP2C19	231	LM6-shLacZ	LM6-shFABP4	LM6-shFABP5	LM6-shCYP2C19	231	LM6-shLacZ	LM6-shFABP4	LM6-shFABP5	LM6-shCYP2C19
CYP2C19	1	3570	5356	3790	3282	1174	3570	5421	3818	3413	1221	1.00	1.52	1.07	0.96	0.34
(54 kDa)	2	1434	3002	1552	1013	1218	1434	2916	1553	1007	1211	1.00	2.03	1.08	0.70	0.84
	3	2896	4231	2383	475	1331	2896	3745	2168	440	1234	1.00	1.29	0.75	0.15	0.43
	Mean						2633	4027	2513	1620	1222	1.00	1.62	0.97	0.60	0.54
	SD						1092	1276	1171	1578	11		0.38	0.19	0.41	0.27
							Lane normalization ratio: actin intensity parental 231 cells (control)/actin intensity of POI lane)									
actin	1	9005	8896	8939	8659	8659	1.00	0.99	0.99	0.96	0.96					
	2	15423	15880	15409	15516	15516	1.00	1.03	1.00	1.01	1.01					
	3	6404	7235	7039	6909	6909	1.00	1.13	1.10	1.08	1.08					
FABP5	1	6713	7788	4534	2173	4855	6713	7588	4514	2095	4894	1.00	1.13	0.67	0.31	0.73
(15 kDa)	2	10475	13463	9139	5809	3156	10475	12387	8966	5983	3190	1.00	1.18	0.86	0.57	0.30
	3	4660	7397	1601	1709	1780	4660	7111	1625	1693	1878	1.00	1.53	0.35	0.36	0.40
	Mean						7283	9029	5035	3257	3321	1.00	1.28	0.63	0.42	0.48
	SD						2949	2918	3698	2369	1512		0.22	0.26	0.14	0.22
							Lane normalization ratio: actin intensity parental 231 cells (control)/actin intensity of POI lane)									
actin	1	16419	16852	16489	17030	16291	1.00	1.03	1.00	1.04	0.99					
	2	8150	8858	8308	7912	8064	1.00	1.09	1.02	0.97	0.99					

	3	21020	21865	20704	21216	19917	1.00	1.04	0.98	1.01	0.95					
FABP4	1	8620	9857	5250	3325	3321	8620	9976	5289	3458	3453	1.00	1.16	0.61	0.40	0.40
(15 kDa)	2	4468	8039	2420	4225	3171	4468	7808	2422	4199	3152	1.00	1.75	0.54	0.94	0.71
	3	6376	7089	3662	5738	2711	6376	6275	3332	5318	2513	1.00	0.98	0.52	0.83	0.39
	Mean						6488	8020	3681	4325	3039	1.00	1.30	0.56	0.72	0.50
	SD						2078	1860	1465	936	480		0.40	0.05	0.29	0.18
							Lane normalization ratio: actin intensity parental 231 cells (control)/actin intensity of POI lane)									
actin	1	9005	8896	8939	8659	8659	1.00	0.99	0.99	0.96	0.96					
	2	15423	15880	15409	15516	15516	1.00	1.03	1.00	1.01	1.01					
	3	6404	7235	7039	6909	6909	1.00	1.13	1.10	1.08	1.08					

Table S15. Densitometry analysis comparing the expression of FABP4, FABP5 and CYP2C19 in parental 231, LM6-shLacZ, LM6-shFABP4, LM6-shFABP5 and LM6-CYP2C19-depleted cells (**Figure 4b; cells co-cultured with 3T3 adipocytes)**. Densitometry software (ImageJ) was used to determine the band intensities for each protein of interest (POI). Band intensities of the corresponding housekeeping protein (actin) were used as loading control (LC) for each lane. Normalized protein intensity for each POI was calculated by multiplying the band intensity by the loading control normalization ratio (ratio of actin intensity in the parental 231 control lane to the actin intensity in the POI lane). Relative protein expression for each POI was calculated as fold change relative to the normalized protein intensity in the parental 231 cells. Statistical analysis was performed using one-way ANOVA, *post hoc* Dunnett's test, with protein expression of the parental 231 cells as control. Red color indicates $p < 0.05$.

**Protein/Experiment**		**Protein/Band Densitometry Intensity from ImageJ**					**Normalized Protein Intensity (POI Intensity*Lane Normalization Ratio)**					**Relative Protein Expression (Fold Change Relative to Expression in 231 Cells)**				
		231	LM6-shLacZ	LM6-shFABP4	LM6-shFABP5	LM6-shCYP2C19	231	LM6-shLacZ	LM6-shFABP4	LM6-shFABP5	LM6-shCYP2C19	231	LM6-shLacZ	LM6-shFABP4	LM6-shFABP5	LM6-shCYP2C19
CYP2C19	1	3570	5356	3790	3850	3331	3570	5421	3818	4003	3464	1.00	1.52	1.07	1.12	0.97
(54 kDa)	2	7735	8927	8994	6250	6016	7735	7905	9129	6549	6304	1.00	1.02	1.18	0.85	0.81
	3	1049	1527	1090	1910	546	1049	1602	1092	1941	555	1.00	1.53	1.04	1.85	0.53
	Mean						4118	4976	4680	4164	3441	1.00	1.36	1.10	1.27	0.77
	SD						3377	3175	4087	2308	2874		0.29	0.07	0.52	0.22
							Lane normalization ratio: actin intensity parental 231 cells (control)/actin intensity of POI lane)									
actin	1	9005	8896	8939	8659	8659	1.00	0.99	0.99	0.96	0.96					
	2	5282	5965	5204	5041	5041	1.00	1.13	0.99	0.95	0.95					
	3	16452	15689	16416	16186	16186	1.00	0.95	1.00	0.98	0.98					
FABP5	1	6376	10489	6662	3738	7171	6376	10617	6711	3887	7457	1.00	1.67	1.05	0.61	1.17
(15 kDa)	2	9725	12866	8865	8063	7885	9725	11394	8999	8449	8262	1.00	1.17	0.93	0.87	0.85
	3	1167	1076	969	976	929	1167	1128	971	992	944	1.00	0.97	0.83	0.85	0.81
	Mean						5756	7713	5560	4443	5554	1.00	1.27	0.94	0.78	0.94
	SD						4312	5716	4136	3759	4013		0.36	0.11	0.14	0.20

							Lane normalization ratio: actin intensity parental 231 cells (control)/actin intensity of POI lane)									
actin	1	9005	8896	8939	8659	8659	1.00	0.99	0.99	0.96	0.96					
	2	5282	5965	5204	5041	5041	1.00	1.13	0.99	0.95	0.95					
	3	16452	15689	16416	16186	16186	1.00	0.95	1.00	0.98	0.98					
FABP5	1	7713	7788	4534	2173	2173	7713	8125	4567	3168	3664	1.00	1.05	0.59	0.41	0.48
(15 kDa)	2	4159	7889	6181	3871	3871	4159	7512	6050	7811	7221	1.00	1.81	1.45	1.88	1.74
	3	1673	2823	3247	925	1925	1673	2927	3312	933	1930	1.00	1.75	1.98	0.56	1.15
	Mean						4515	6188	4643	3971	4272	1.00	1.54	1.34	0.95	1.12
	SD						3036	2841	1371	3508	2698		0.42	0.70	0.81	0.63
							Lane normalization ratio: actin intensity parental 231 cells (control)/actin intensity of POI lane)									
actin	1	10652	10211	10575	7307	6318	1.00	0.96	0.99	0.69	0.59					
	2	20392	21414	20832	10106	10931	1.00	1.05	1.02	0.50	0.54					
	3	9120	8797	8942	9040	9098	1.00	0.96	0.98	0.99	1.00					

Table S16. Densitometry analysis comparing the expression of FABP4, FABP5 and CYP2C19 in parental 231, LM6-shLacZ, LM6-shFABP4, LM6-shFABP5 and LM6-CYP2C19-depleted cells (Figure 4b; cells co-cultured with 3T3 fibroblasts). Densitometry software (ImageJ) was used to determine the band intensities for each protein of interest (POI). Band intensities of the corresponding housekeeping protein (actin) were used as loading control (LC) for each lane. Normalized protein intensity for each POI was calculated by multiplying the band intensity by the loading control normalization ratio (ratio of actin intensity in the parental 231 control lane to the actin intensity in the POI lane. Relative protein expression for each POI was calculated as fold change relative to the normalized protein intensity in the parental 231 cells. Mean relative protein expressions and standard deviations for three independent western blotting experiments were obtained and statistical analysis was performed using one-way ANOVA, *post hoc* Dunnett's test, with protein expression of the parental 231 cells as control. Red color indicates $p < 0.05$.

**Protein/Experiment**		**Protein/Band Densitometry Intensity from ImageJ**					**Normalized Protein Intensity (POI Intensity*Lane Normalization Ratio)**					**Relative Protein Expression (Fold Change Relative to Expression in 231 Cells)**				
		231	LM6-shLacZ	LM6-shFABP4	LM6-shFABP5	LM6-shCYP2C19	231	LM6-shLacZ	LM6-shFABP4	LM6-shFABP5	LM6-shCYP2C19	231	LM6-shLacZ	LM6-shFABP4	LM6-shFABP5	LM6-shCYP2C19

CYP2C19	1	3570	4356	3790	3450	3450	3570	4354	4040	3632	3823	1.00	1.22	1.13	1.02	1.07
(54 kDa)	2	6500	7385	8779	8193	8193	6500	7663	9211	8642	8460	1.00	1.18	1.42	1.33	1.30
	3	1345	1390	596	682	682	1345	1266	564	684	723	1.00	0.94	0.42	0.51	0.54
	Mean						3805	4428	4605	4319	4335	1.00	1.11	0.99	0.95	0.97
	SD						2586	3199	4351	4023	3894		0.15	0.51	0.41	0.39
							Lane normalization ratio: actin intensity parental 231 cells (control)/actin intensity of POI lane)									
actin	1	11180	11184	10489	10618	10087	1.00	1.00	0.94	0.95	0.90					
	2	25847	24907	24637	24505	25033	1.00	0.96	0.95	0.95	0.97					
	3	6281	6895	6640	6266	5928	1.00	1.10	1.06	1.00	0.94					
FABP5	1	7713	7788	7534	2173	7173	7713	7785	8029	2288	7951	1.00	1.01	1.04	0.30	1.03
(15 kDa)	2	39882	39031	17686	3288	35288	39882	40504	18555	3468	36435	1.00	1.02	0.47	0.09	0.91
	3	753	1438	1576	671	671	753	1310	1491	673	711	1.00	1.74	1.98	0.89	0.95
	Mean						16116	16533	9358	2143	15032	1.00	1.26	1.16	0.43	0.96
	SD						20874	21011	8609	1403	18886		0.42	0.77	0.42	0.06
							Lane normalization ratio: actin intensity parental 231 cells (control)/actin intensity of POI lane)									
actin	1	11180	11184	10489	10618	10087	1.00	1.00	0.94	0.95	0.90					
	2	25847	24907	24637	24505	25033	1.00	0.96	0.95	0.95	0.97					
	3	6281	6895	6640	6266	5928	1.00	1.10	1.06	1.00	0.94					
FABP4	1	6376	10489	3662	5738	6738	6376	10369	3688	5816	6649	1.00	1.63	0.58	0.91	1.04
(15 kDa)	2	39791	17894	8891	23257	33257	39791	19783	9472	24146	32740	1.00	0.50	0.24	0.61	0.82
	3	561	414	419	606	506	561	414	409	596	504	1.00	0.74	0.73	1.06	0.90
	Mean						15576	10189	4523	10186	13298	1.00	0.95	0.52	0.86	0.92
	SD						21172	9686	4589	12368	17116		0.59	0.25	0.23	0.11

							Lane normalization ratio: actin intensity parental 231 cells (control)/actin intensity of POI lane)				
actin	1	18168	18379	18043	17925	18411	1.00	1.01	0.99	0.99	1.01
	2	8737	7902	8200	8415	8875	1.00	0.90	0.94	0.96	1.02
	3	29314	29324	29969	29787	29415	1.00	1.00	1.02	1.02	1.00

Table S17. Densitometry analysis comparing the expression of FABP4, FABP5 and CYP2C19 in parental 231, LM6-shLacZ, LM6-shFABP4, LM6-shFABP5 and LM6-CYP2C19-depleted cells (Figure 4b; cells co-cultured with THP-1 monocytes). Densitometry software (ImageJ) was used to determine the band intensities for each protein of interest (POI). Band intensities of the corresponding housekeeping protein (actin) were used as loading control (LC) for each lane. Normalized protein intensity for each POI was calculated by multiplying the band intensity by the loading control normalization ratio (ratio of actin intensity in the parental 231 control lane to the actin intensity in the POI lane). Relative protein expression for each POI was calculated as fold change relative to the normalized protein intensity of parental 231 cells. Mean relative protein expressions and standard deviations for three independent western blotting experiments were obtained and statistical analysis was performed using one-way ANOVA, *post hoc* Dunnett's test, with protein expression of the parental 231 cells as control. Red color indicates $p < 0.05$.

**Protein/Experiment**		**Protein/Band Densitometry Intensity from ImageJ**					**Normalized Protein Intensity (POI Intensity*Lane Normalization Ratio)**					**Relative Protein Expression (Fold Change Relative to Expression in 231 Cells)**				
		231	LM6-shLacZ	LM6-shFABP4	LM6-shFABP5	LM6-shCYP2C19	231	LM6-shLacZ	LM6-shFABP4	LM6-shFABP5	LM6-shCYP2C19	231	LM6-shLacZ	LM6-shFABP4	LM6-shFABP5	LM6-shCYP2C19
CYP2C19	1	3570	4356	3790	3450	3450	3570	4275	3702	3478	5251	1.00	1.20	1.04	0.97	1.47
(54 kDa)	2	6500	7385	8779	8193	8193	6500	7179	9566	8390	1497	1.00	1.10	1.47	1.29	0.23
	3	1345	1390	596	682	682	1345	1227	636	573	810	1.00	0.91	0.47	0.43	0.60
	Mean						3805	4227	4635	4147	2519	1.00	1.07	0.99	0.90	0.77
	SD						2586	2976	4538	3952	2390		0.15	0.50	0.44	0.64
							Lane normalization ratio: actin intensity parental 231 cells (control)/actin intensity of POI lane)									
actin	1	8731	8896	8939	8659	5736	1.00	1.02	1.02	0.99	0.66					
	2	5874	6043	5391	5736	32157	1.00	1.03	0.92	0.98	5.47					
	3	27001	30583	25308	32157	22735	1.00	1.13	0.94	1.19	0.84					
FABP5	1	7713	7788	7534	2173	7173	7713	7644	7359	2192	10919	1.00	0.99	0.95	0.28	1.42
(15 kDa)	2	39882	39031	17686	3288	35288	39882	37943	19271	3367	6446	1.00	0.95	0.48	0.08	0.16

	3	753	1438	1576	671	671	753	1269	1682	564	797	1.00	1.69	2.23	0.75	1.06
	Mean						16116	15619	9437	2041	6054	1.00	1.21	1.22	0.37	0.88
	SD						20874	19595	8977	1408	5072		0.41	0.91	0.34	0.65
							Lane normalization ratio: actin intensity parental 231 cells (control)/actin intensity of POI lane)									
actin	1	8731	8896	8939	8659	5736	1.00	1.02	1.02	0.99	0.66					
	2	5874	6043	5391	5736	32157	1.00	1.03	0.92	0.98	5.47					
	3	27001	30583	25308	32157	22735	1.00	1.13	0.94	1.19	0.84					
FABP4	1	6376	10489	3662	5738	6738	6376	10979	3763	5973	6851	1.00	1.72	0.59	0.94	1.07
(15 kDa)	2	39791	17894	8891	23257	33257	39791	19147	9199	22898	32743	1.00	0.48	0.23	0.58	0.82
	3	561	414	419	606	506	561	416	426	591	514	1.00	0.74	0.76	1.05	0.92
	Mean						15576	10181	4463	9821	13369	1.00	0.98	0.53	0.85	0.94
	SD						21172	9391	4428	11641	17075		0.65	0.27	0.25	0.13
							Lane normalization ratio: actin intensity parental 231 cells (control)/actin intensity of POI lane)									
actin	1	18692	17858	18190	17954	18383	1.00	0.96	0.97	0.96	0.98					
	2	7950	7430	7683	8074	8074	1.00	0.93	0.97	1.02	1.02					
	3	10067	10003	9893	10320	9896	1.00	0.99	0.98	1.03	0.98					

Table S18. Densitometry analysis comparing the expression of FABP-EET network markers in MDA-MB-231, LM6 and FABP4/FABP5CYP2C19-depleted cells (Figure 4f; cells grown in monoculture). Densitometry software (ImageJ) was used to determine the band intensities for each protein of interest (POI). Band intensities of the corresponding housekeeping protein (actin) were used as loading control (LC) for each lane. Normalized protein intensity for each POI was calculated by multiplying the band intensity by the loading control normalization ratio (ratio of actin intensity in the parental 231 control lane to the actin intensity in the POI lane). Relative protein expression for each POI was calculated as fold change relative to the normalized protein intensity of parental 231 cells. Mean relative protein expressions and standard deviations for three independent western blotting experiments were obtained and statistical analysis was performed using one-way ANOVA, *post hoc* Dunnett's test, with protein expression of the the parental 231 cells as control. Red color indicates $p < 0.05$.

**Protein/Experiment**	**Protein/Band Densitometry Intensity from ImageJ**	**Normalized Protein Intensity (POI Intensity*Lane Normalization Ratio)**	**Relative Protein Expression (Fold Change Relative to Expression in 231 Cells)**

		231	LM6-shLacZ	LM6-shFABP4	LM6-shFABP5	LM6-shCYP2C19	231	LM6-shLacZ	LM6-shFABP4	LM6-shFABP5	LM6-shCYP2C19	231	LM6-shLacZ	LM6-shFABP4	LM6-shFABP5	LM6-shCYP2C19
CD36	1	8732	12624	3177	4091	5249	8732	12545	3276	4230	5125	1.00	1.44	0.38	0.48	0.59
(85 kDa)	2	17173	15052	9694	12094	12860	17173	15166	10015	11941	12423	1.00	0.88	0.58	0.70	0.72
	3	5204	8040	172	648	4218	5204	7541	163	602	4076	1.00	1.45	0.03	0.12	0.78
	Mean						10369	11751	4485	5591	7208	1.00	1.26	0.33	0.43	0.70
	SD						6150	3874	5036	5791	4547		0.32	0.28	0.29	0.10
							Lane normalization ratio: actin intensity parental 231 cells (control)/actin intensity of POI lane)									
actin	1	8521	8575	8264	8242	8728	1.00	1.01	0.97	0.97	1.02					
	2	17773	17641	17203	18002	18399	1.00	0.99	0.97	1.01	1.04					
	3	26169	27901	27494	28163	27083	1.00	1.07	1.05	1.08	1.03					
c-myc	1	3950	2592	2612	5671	4102	3950	2717	2570	5696	4120	1.00	0.69	0.65	1.44	1.04
(57 kDa)	2	9811	7269	9287	9276	9187	9811	7318	9380	9294	9129	1.00	0.75	0.96	0.95	0.93
	3	4343	6571	5189	3857	5180	4343	6627	5210	3878	5304	1.00	1.53	1.20	0.89	1.22
	Mean						6035	5554	5720	6289	6184	1.00	0.99	0.94	1.09	1.06
	SD						3277	2481	3434	2756	2618		0.47	0.28	0.30	0.15
							Lane normalization ratio: actin intensity parental 231 cells (control)/actin intensity of POI lane)									
actin	1	11019	10515	11200	10971	10971	1.00	0.95	1.02	1.00	1.00					
	2	36516	36269	36152	36445	36746	1.00	0.99	0.99	1.00	1.01					
	3	19308	19144	19229	19202	18857	1.00	0.99	1.00	0.99	0.98					
Sox-2	1	6588	7824	5913	3892	7564	6588	8456	6116	4053	7494	1.00	1.28	0.93	0.62	1.14
(34 kDa)	2	12242	11806	13397	10067	13084	12242	13455	11367	8333	14310	1.00	1.10	0.93	0.68	1.17
	3	2576	2201	2486	688	1937	2576	2471	2705	810	2237	1.00	0.96	1.05	0.31	0.87

	Mean						7135	8127	6729	4398	8014	1.00	1.11	0.97	0.54	1.06
	SD						4856	5499	4363	3773	6053		0.16	0.07	0.20	0.17
							Lane normalization ratio: actin intensity parental 231 cells (control)/actin intensity of POI lane)									
actin	1	9896	9157	9567	9504	9989	1.00	0.93	0.97	0.96	1.01					
	2	22401	19657	26402	27064	20482	1.00	0.88	1.18	1.21	0.91					
	3	5856	5215	5381	4974	5071	1.00	0.89	0.92	0.85	0.87					
Vimentin	1	8424	10864	6020	4803	7424	8424	10268	6215	4716	6986	1.00	1.22	0.74	0.56	0.83
(57 kDa)	2	20130	16314	14600	12127	15130	20130	15806	14538	12196	14780	1.00	0.79	0.72	0.61	0.73
	3	2299	3223	2172	1647	1298	2299	3203	2186	1655	1317	1.00	1.39	0.95	0.72	0.57
	Mean						10284	9759	7646	6189	7694	1.00	1.13	0.80	0.63	0.71
	SD						9060	6317	6299	5423	6760		0.31	0.13	0.08	0.13
							Lane normalization ratio: actin intensity parental 231 cells (control)/actin intensity of POI lane)									
actin	1	9270	9807	8978	9442	9851	1.00	1.06	0.97	1.02	1.06					
	2	24130	24906	24234	23993	24701	1.00	1.03	1.00	0.99	1.02					
	3	35580	35799	35347	35404	35064	1.00	1.01	0.99	1.00	0.99					
p-Src (419)	1	6465	4160	3300	3326	4291	6465	4360	3246	3340	4310	1.00	0.67	0.50	0.52	0.67
(60 kDa)	2	15346	18249	10420	10522	12943	15346	18373	10524	10542	12862	1.00	1.20	0.69	0.69	0.84
	3	3463	6249	1423	1472	2463	3463	6303	1429	1480	2522	1.00	1.82	0.41	0.43	0.73
	Mean						8425	9678	5067	5121	6565	1.00	1.23	0.53	0.54	0.74
	SD						6179	7592	4813	4786	5526		0.57	0.14	0.13	0.09
							Lane normalization ratio: actin intensity parental 231 cells (control)/actin intensity of POI lane)									
actin	1	11019	10515	11200	10971	10971	1.00	0.95	1.02	1.00	1.00					
	2	36516	36269	36152	36445	36746	1.00	0.99	0.99	1.00	1.01					
	3	19308	19144	19229	19202	18857	1.00	0.99	1.00	0.99	0.98					

p-Src(527)	1	6159	4071	5920	5055	5097	6159	4400	6123	5264	5050	1.00	0.71	0.99	0.85	0.82
(60 kDa)	2	15938	11355	15579	15324	14097	15938	12940	13218	12684	15418	1.00	0.81	0.83	0.80	0.97
	3	3310	1028	3610	2892	3010	3310	1154	3928	3404	3476	1.00	0.35	1.19	1.03	1.05
	Mean						8469	6165	7757	7117	7981	1.00	0.62	1.00	0.89	0.95
	SD						6623	6088	4856	4910	6488		0.24	0.18	0.12	0.12
							Lane normalization ratio: actin intensity parental 231 cells (control)/actin intensity of POI lane)									
actin	1	9896	9157	9567	9504	9989	1.00	0.93	0.97	0.96	1.01					
	2	22401	19657	26402	27064	20482	1.00	0.88	1.18	1.21	0.91					
	3	5856	5215	5381	4974	5071	1.00	0.89	0.92	0.85	0.87					
Src	1	3710	3786	3894	3989	3497	3710	3578	4020	3916	3291	1.00	0.96	1.08	1.06	0.89
(60 kDa)	2	9416	9785	9422	9553	10416	9416	9480	9382	9608	10175	1.00	1.01	1.00	1.02	1.08
	3	2985	2022	2747	2676	3097	2985	2010	2765	2689	3143	1.00	0.67	0.93	0.90	1.05
	Mean						5370	5023	5389	5405	5536	1.00	0.88	1.00	0.99	1.01
	SD						3522	3939	3514	3692	4018		0.18	0.08	0.08	0.10
							Lane normalization ratio: actin intensity parental 231 cells (control)/actin intensity of POI lane)									
actin	1	9270	9807	8978	9442	9851	1.00	1.06	0.97	1.02	1.06					
	2	24130	24906	24234	23993	24701	1.00	1.03	1.00	0.99	1.02					
	3	35580	35799	35347	35404	35064	1.00	1.01	0.99	1.00	0.99					
p-FAK	1	5589	5141	5067	4926	5589	5589	5109	5225	5093	5456	1.00	0.91	0.93	0.91	0.98
(125 kDa)	2	14387	13089	10205	10272	16873	14387	13188	10544	10142	16299	1.00	0.92	0.73	0.70	1.13
	3	1755	3679	2064	2106	1937	1755	3451	1965	1957	1872	1.00	1.97	1.12	1.12	1.07
	Mean						7243	7249	5911	5730	7876	1.00	1.27	0.93	0.91	1.06
	SD						6477	5209	4331	4130	7512		0.61	0.19	0.21	0.08
							Lane normalization ratio: actin intensity parental 231 cells (control)/actin intensity of POI lane)									

actin	1	8521	8575	8264	8242	8728	1.00	1.01	0.97	0.97	1.02					
	2	17773	17641	17203	18002	18399	1.00	0.99	0.97	1.01	1.04					
	3	26169	27901	27494	28163	27083	1.00	1.07	1.05	1.08	1.03					
FAK	1	8124	10070	8090	7170	8124	8124	9518	8352	7039	7645	1.00	1.17	1.03	0.87	0.94
(125 kDa)	2	18503	17152	16549	18208	18503	18503	16618	16478	18312	18075	1.00	0.90	0.89	0.99	0.98
	3	4654	4945	6204	4068	4654	4654	4915	6245	4089	4723	1.00	1.06	1.34	0.88	1.01
	Mean						10427	10350	10358	9813	10148	1.00	1.04	1.09	0.91	0.98
	SD						7206	5896	5403	7507	7019		0.14	0.23	0.07	0.04
							Lane normalization ratio: actin intensity parental 231 cells (control)/actin intensity of POI lane)									
actin	1	9270	9807	8978	9442	9851	1.00	1.06	0.97	1.02	1.06					
	2	24130	24906	24234	23993	24701	1.00	1.03	1.00	0.99	1.02					
	3	35580	35799	35347	35404	35064	1.00	1.01	0.99	1.00	0.99					
CD44	1	6475	4970	8174	4132	5138	6475	4939	8428	4272	5016	1.00	0.76	1.30	0.66	0.77
(80 kDa)	2	17888	21687	16179	10903	15888	17888	21850	16716	10764	15348	1.00	1.22	0.93	0.60	0.86
	3	4715	4058	3328	392	3187	4715	3806	3167	364	3079	1.00	0.81	0.67	0.08	0.65
	Mean						9693	10198	9437	5134	7815	1.00	0.93	0.97	0.45	0.76
	SD						7152	10107	6831	5253	6595		0.25	0.32	0.32	0.10
							Lane normalization ratio: actin intensity parental 231 cells (control)/actin intensity of POI lane)									
actin	1	8521	8575	8264	8242	8728	1.00	1.01	0.97	0.97	1.02					
	2	17773	17641	17203	18002	18399	1.00	0.99	0.97	1.01	1.04					
	3	26169	27901	27494	28163	27083	1.00	1.07	1.05	1.08	1.03					
Ezrin	1	5319	6978	4601	3598	5319	5319	7541	4759	3747	5270	1.00	1.42	0.89	0.70	0.99
(69 kDa)	2	13744	14570	14682	12503	13744	13744	16604	12457	10349	15032	1.00	1.21	0.91	0.75	1.09
	3	1284	1108	1046	504	1284	1284	1244	1138	593	1482	1.00	0.97	0.89	0.46	1.15

	Mean						6782	8463	6118	4896	7261	1.00	1.20	0.90	0.64	1.08
	SD						6358	7721	5781	4979	6991		0.22	0.01	0.16	0.08
							Lane normalization ratio: actin intensity parental 231 cells (control)/actin intensity of POI lane)									
actin	1	9896	9157	9567	9504	9989	1.00	0.93	0.97	0.96	1.01					
	2	22401	19657	26402	27064	20482	1.00	0.88	1.18	1.21	0.91					
	3	5856	5215	5381	4974	5071	1.00	0.89	0.92	0.85	0.87					

Table S19. Densitometry analysis comparing the expression of FABP-EET network markers in MDA-MB-231, LM6 and FABP4/FABP5CYP2C19-depleted cells (Figure 4f; cells co-cultured with 3T3 fibroblasts). Densitometry software (ImageJ) was used to determine the band intensities for each protein of interest (POI). Band intensities of the corresponding housekeeping protein (actin) were used as loading control (LC) for each lane. Normalized protein intensity for each POI was calculated by multiplying the band intensity by the loading control normalization ratio (ratio of actin intensity in the parental 231 control lane to the actin intensity in the POI lane). Relative protein expression for each POI was calculated as fold change relative to the normalized protein intensity in the parental 231 cells. Mean relative protein expressions and standard deviations for three independent western blotting experiments were obtained and statistical analysis was performed using one-way ANOVA, *post hoc* Dunnett's test, with protein expression of the the parental 231 cells as control. Red color indicates $p < 0.05$.

Protein/Experiment		Protein/Band Densitometry Intensity from ImageJ					Normalized Protein Intensity (POI Intensity*Lane Normalization Ratio)					Relative Protein Expression (Fold Change Relative to Expression in 231 Cells)				
		231	LM6-shLacZ	LM6-shFABP4	LM6-shFABP5	LM6-shCYP2C19	231	LM6-shLacZ	LM6-shFABP4	LM6-shFABP5	LM6-shCYP2C19	231	LM6-shLacZ	LM6-shFABP4	LM6-shFABP5	LM6-shCYP2C19
CD36	1	5624	6732	3177	4091	5594	5624	6577	3277	4062	5723	1.00	1.17	0.58	0.72	1.02
(85 kDa)	2	15052	17173	9694	10042	9498	15052	16944	9793	9974	9456	1.00	1.13	0.65	0.66	0.63
	3	1040	1024	917	648	594	1040	1018	925	647	598	1.00	0.98	0.89	0.62	0.57
	Mean						7239	8180	4665	4894	5259	1.00	1.09	0.71	0.67	0.74
	SD						7144	8083	4594	4719	4448		0.10	0.16	0.05	0.24
							Lane normalization ratio: actin intensity parental 231 cells (control)/actin intensity of POI lane									
actin	1	8792	8998	8523	8855	8594	1.00	1.02	0.97	1.01	0.98					
	2	18417	18666	18230	18543	18498	1.00	1.01	0.99	1.01	1.00					
	3	32792	32998	32523	32855	32594	1.00	1.01	0.99	1.00	0.99					
c-myc	1	2592	2950	2612	2558	1950	2592	2849	2480	2437	1842	1.00	1.10	0.96	0.94	0.71
(57 kDa)	2	7269	7811	7832	8337	6811	7269	7876	7715	8290	6814	1.00	1.08	1.06	1.14	0.94
	3	735	987	603	815	833	735	1027	623	861	845	1.00	1.40	0.85	1.17	1.15
	Mean						3532	3917	3606	3863	3167	1.00	1.19	0.96	1.08	0.93
	SD						3367	3547	3678	3914	3198		0.18	0.11	0.13	0.22
							Lane normalization ratio: actin intensity parental 231 cells (control)/actin intensity of POI lane									
actin	1	8049	8333	8477	8446	8522	1.00	1.04	1.05	1.05	1.06					

	2	32481	32214	32972	32669	32465	1.00	0.99	1.02	1.01	1.00				
	3	15875	15254	15351	15023	15645	1.00	0.96	0.97	0.95	0.99				
Sox-2	1	4090	4588	3923	3892	5588	4090	4726	3710	4574	5803	1.00	1.16	0.91	1.12	1.42
(37 kDa)	2	11806	11242	10133	10067	12242	11806	12038	10037	10506	12001	1.00	1.02	0.85	0.89	1.02
	3	2201	3576	2838	1688	2576	2201	3710	2903	1697	2594	1.00	1.69	1.32	0.77	1.18
	Mean						6032	6824	5550	5592	6800	1.00	1.29	1.03	0.93	1.20
	SD						5089	4543	3907	4492	4782		0.35	0.26	0.18	0.20
							Lane normalization ratio: actin intensity parental 231 cells (control)/actin intensity of POI lane									
actin	1	3672	3565	3883	3125	3536	1.00	0.97	1.06	0.85	0.96					
	2	8771	8191	8854	8404	8947	1.00	0.93	1.01	0.96	1.02					
	3	29181	28127	28531	29032	28969	1.00	0.96	0.98	0.99	0.99					
Vimentin	1	5416	5424	6020	4803	4468	5416	5439	6605	4963	4717	1.00	1.00	1.22	0.92	0.87
(57 kDa)	2	14317	20130	14600	12127	10130	14317	19914	14522	12093	9948	1.00	1.39	1.01	0.84	0.69
	3	3323	4299	2172	1647	2299	3323	3950	2350	1524	2379	1.00	1.19	0.71	0.46	0.72
	Mean						7685	9768	7826	6194	5681	1.00	1.19	0.98	0.74	0.76
	SD						5838	8818	6177	5391	3875		0.19	0.26	0.25	0.10
							Lane normalization ratio: actin intensity parental 231 cells (control)/actin intensity of POI lane									
actin	1	12875	12839	11735	12459	12196	1.00	1.00	0.91	0.97	0.95					
	2	21864	22102	21982	21925	22265	1.00	1.01	1.01	1.00	1.02					
	3	5429	5908	5019	5865	5246	1.00	1.09	0.92	1.08	0.97					
p-Src (419)	1	4160	4465	3300	3326	4937	4160	4313	3133	3169	4663	1.00	1.04	0.75	0.76	1.12
(60 kDa)	2	12202	12436	10420	10522	12973	12202	12539	10264	10461	12979	1.00	1.03	0.84	0.86	1.06
	3	3432	3463	1423	4472	3463	3432	3604	1472	4725	3514	1.00	1.05	0.43	1.38	1.02

	Mean						6598	6819	4956	6119	7052	1.00	1.04	0.67	1.00	1.07
	SD						4867	4967	4671	3840	5165		0.01	0.22	0.33	0.05
							Lane normalization ratio: actin intensity parental 231 cells (control)/actin intensity of POI lane									
actin	1	8049	8333	8477	8446	8522	1.00	1.04	1.05	1.05	1.06					
	2	32481	32214	32972	32669	32465	1.00	0.99	1.02	1.01	1.00					
	3	15875	15254	15351	15023	15645	1.00	0.96	0.97	0.95	0.99					
p-Src(527)	1	4971	6159	5920	5055	6159	4971	6344	5598	5941	6397	1.00	1.28	1.13	1.20	1.29
(60 kDa)	2	13555	15938	15579	11324	15938	13555	17067	15432	11819	15625	1.00	1.26	1.14	0.87	1.15
	3	5578	3310	609	5876	3310	5578	3434	623	5906	3334	1.00	0.62	0.11	1.06	0.60
	Mean						8035	8948	7218	7889	8452	1.00	1.05	0.79	1.04	1.01
	SD						4791	7180	7536	3404	6398		0.38	0.59	0.16	0.37
							Lane normalization ratio: actin intensity parental 231 cells (control)/actin intensity of POI lane									
actin	1	3672	3565	3883	3125	3536	1.00	0.97	1.06	0.85	0.96					
	2	8771	8191	8854	8404	8947	1.00	0.93	1.01	0.96	1.02					
	3	29181	28127	28531	29032	28969	1.00	0.96	0.98	0.99	0.99					
Src	1	2785	3710	2894	1987	2780	2785	3720	3175	2053	2935	1.00	1.34	1.14	0.74	1.05
(60 kDa)	2	7785	8476	6422	6553	9416	7785	8385	6388	6535	9246	1.00	1.08	0.82	0.84	1.19
	3	1022	1998	937	1676	1035	1022	1836	1014	1552	1071	1.00	1.80	0.99	1.52	1.05
	Mean						3864	4647	3525	3380	4417	1.00	1.40	0.98	1.03	1.10
	SD						3508	3371	2704	2744	4284		0.36	0.16	0.42	0.08
							Lane normalization ratio: actin intensity parental 231 cells (control)/actin intensity of POI lane									
actin	1	12875	12839	11735	12459	12196	1.00	1.00	0.91	0.97	0.95					
	2	21864	22102	21982	21925	22265	1.00	1.01	1.01	1.00	1.02					

	3	5429	5908	5019	5865	5246	1.00	1.09	0.92	1.08	0.97					
p-FAK	1	3141	5589	2073	3503	3589	3141	5460	2139	3478	3672	1.00	1.74	0.68	1.11	1.17
(125 kDa)	2	9105	6824	9205	10272	10385	9105	6733	9300	10203	10340	1.00	0.74	1.02	1.12	1.14
	3	1679	1055	1238	1181	1057	1679	1048	1248	1179	1063	1.00	0.62	0.74	0.70	0.63
	Mean						4642	4414	4229	4953	5025	1.00	1.03	0.82	0.98	0.98
	SD						3934	2983	4414	4689	4784		0.61	0.18	0.24	0.30
							Lane normalization ratio: actin intensity parental 231 cells (control)/actin intensity of POI lane)									
actin	1	8792	8998	8523	8855	8594	1.00	1.02	0.97	1.01	0.98					
	2	18417	18666	18230	18543	18498	1.00	1.01	0.99	1.01	1.00					
	3	32792	32998	32523	32855	32594	1.00	1.01	0.99	1.00	0.99					
FAK	1	7070	8124	7090	7170	8124	7070	8147	7779	7409	8577	1.00	1.15	1.10	1.05	1.21
(125 kDa)	2	17152	18503	16549	18208	18503	17152	18304	16460	18157	18170	1.00	1.07	0.96	1.06	1.06
	3	1945	3497	1204	2068	2037	1945	3213	1302	1914	2108	1.00	1.65	0.67	0.98	1.08
	Mean						8722	9888	8514	9160	9618	1.00	1.29	0.91	1.03	1.12
	SD						7737	7695	7606	8262	8081		0.32	0.22	0.04	0.08
							Lane normalization ratio: actin intensity parental 231 cells (control)/actin intensity of POI lane)									
actin	1	12875	12839	11735	12459	12196	1.00	1.00	0.91	0.97	0.95					
	2	21864	22102	21982	21925	22265	1.00	1.01	1.01	1.00	1.02					
	3	5429	5908	5019	5865	5246	1.00	1.09	0.92	1.08	0.97					
CD44	1	4970	6475	4170	5239	4483	4970	6327	4301	5202	4586	1.00	1.27	0.87	1.05	0.92
(80 kDa)	2	13662	17888	12179	10903	17888	13662	17650	12305	10829	17810	1.00	1.29	0.90	0.79	1.30
	3	4458	4715	5314	3392	4715	4458	4686	5358	3385	4744	1.00	1.05	1.20	0.76	1.06
	Mean						7697	9554	7321	6472	9047	1.00	1.21	0.99	0.87	1.10
	SD						5173	7059	4348	3881	7590		0.13	0.18	0.16	0.19

							Lane normalization ratio: actin intensity parental 231 cells (control)/actin intensity of POI lane									
actin	1	8792	8998	8523	8855	8594	1.00	1.02	0.97	1.01	0.98					
	2	18417	18666	18230	18543	18498	1.00	1.01	0.99	1.01	1.00					
	3	32792	32998	32523	32855	32594	1.00	1.01	0.99	1.00	0.99					
Ezrin	1	4748	5319	5601	4070	5319	4748	5479	5296	4783	5524	1.00	1.15	1.12	1.01	1.16
(69 kDa)	2	11570	13744	10385	10503	10352	11570	14717	10287	10962	10148	1.00	1.27	0.89	0.95	0.88
	3	4398	5589	3597	3655	5384	4398	5798	3679	3674	5423	1.00	1.32	0.84	0.84	1.23
	Mean						6905	8665	6421	6473	7032	1.00	1.25	0.95	0.93	1.09
	SD						4043	5244	3445	3927	2699		0.08	0.15	0.09	0.19
							Lane normalization ratio: actin intensity parental 231 cells (control)/actin intensity of POI lane									
actin	1	3672	3565	3883	3125	3536	1.00	0.97	1.06	0.85	0.96					
	2	8771	8191	8854	8404	8947	1.00	0.93	1.01	0.96	1.02					
	3	29181	28127	28531	29032	28969	1.00	0.96	0.98	0.99	0.99					

Table S20. Densitometry analysis comparing the expression of FABP-EET network markers in MDA-MB-231, LM6 and FABP4/FABP5CYP2C19-depleted cells (Figure 4f; cells co-cultured with 3T3 differentiated adipocytes). Densitometry software (ImageJ) was used to determine the band intensities for each protein of interest (POI). Band intensities of the corresponding housekeeping protein (actin) were used as loading control (LC) for each lane. Normalized protein intensity for each POI was calculated by multiplying the band intensity by the loading control normalization ratio (ratio of actin intensity in the parental 231 control lane to the actin intensity in the POI lane). Relative protein expression for each POI was calculated as fold change relative to the normalized protein intensity of parental 231 cells. Mean relative protein expressions and standard deviations for three independent western blotting experiments were obtained and statistical analysis was performed using one-way ANOVA, *post hoc* Dunnett's test, with protein expression of the parental 231 cells as control. Red color indicates $p < 0.05$.

Protein/Experiment		Protein/Band Densitometry Intensity from ImageJ					Normalized Protein Intensity (POI Intensity*Lane Normalization Ratio)					Relative Protein Expression (Fold Change Relative to Expression in 231 Cells)				
		231	LM6-shLacZ	LM6-shFABP4	LM6-shFABP5	LM6-shCYP2C19	231	LM6-shLacZ	LM6-shFABP4	LM6-shFABP5	LM6-shCYP2C19	231	LM6-shLacZ	LM6-shFABP4	LM6-shFABP5	LM6-shCYP2C19
CD36	1	8031	8895	8876	5778	6031	8031	9387	8834	6694	6549	1.00	1.17	1.10	0.83	0.82
(85 kDa)	2	19187	20587	23498	17167	13187	19187	19709	23577	16590	13007	1.00	1.03	1.23	0.86	0.68
	3	24592	24790	29542	20705	20623	24592	24112	29226	20695	20453	1.00	0.98	1.19	0.84	0.83
	Mean						17270	17736	20546	14660	13337	1.00	1.06	1.17	0.85	0.78
	SD						8445	7558	10529	7197	6958		0.10	0.07	0.02	0.08
							Lane normalization ratio: actin intensity parental 231 cells (control)/actin intensity of POI lane									
actin	1	5848	5541	5876	5048	5386	1.00	0.95	1.00	0.86	0.92					
	2	12177	12719	12136	12600	12345	1.00	1.04	1.00	1.03	1.01					
	3	21109	21702	21337	21119	21284	1.00	1.03	1.01	1.00	1.01					
c-myc	1	5542	6278	5976	3807	4442	5542	6282	5766	3677	4074	1.00	1.13	1.04	0.66	0.74
(57 kDa)	2	14337	16106	15831	9880	14337	14337	15328	16006	9380	12856	1.00	1.07	1.12	0.65	0.90
	3	27519	34368	32104	15785	22519	27519	33636	32189	16137	22802	1.00	1.22	1.17	0.59	0.83
	Mean						15799	18415	17987	9731	13244	1.00	1.14	1.11	0.63	0.82
	SD						11061	13936	13322	6238	9370		0.08	0.06	0.04	0.08

							Lane normalization ratio: actin intensity parental 231 cells (control)/actin intensity of POI lane)									
actin	1	4388	4385	4548	4544	4784	1.00	1.00	1.04	1.04	1.09					
	2	11568	12155	11441	12184	12901	1.00	1.05	0.99	1.05	1.12					
	3	38989	39837	38886	38137	38505	1.00	1.02	1.00	0.98	0.99					
Sox-2	1	13857	18336	7837	8994	13857	13857	18787	8289	9683	14496	1.00	1.36	0.60	0.70	1.05
(37 kDa)	2	30499	28798	17987	30197	23499	30499	28658	18059	3029	23469	1.00	0.94	0.59	0.10	0.77
	3	1074	2057	1040	977	954	1074	2072	1155	1093	1036	1.00	1.93	1.08	1.02	0.96
	Mean						15143	16506	9168	4602	13000	1.00	1.41	0.76	0.61	0.93
	SD						14755	13439	8486	4506	11291		0.50	0.28	0.47	0.14
							Lane normalization ratio: actin intensity parental 231 cells (control)/actin intensity of POI lane)									
actin	1	10838	10578	10247	10066	10360	1.00	0.98	0.95	0.93	0.96					
	2	23011	23123	22919	229419	23041	1.00	1.00	1.00	9.97	1.00					
	3	6654	6607	5991	5947	6127	1.00	0.99	0.90	0.89	0.92					
Vimentin	1	4324	5215	5162	6440	4324	4324	5117	5123	6214	4295	1.00	1.18	1.18	1.44	0.99
(57 kDa)	2	10650	12667	8332	12681	10650	10650	12880	8798	12007	10020	1.00	1.21	0.83	1.13	0.94
	3	18215	23415	15741	18266	18215	18215	19088	15031	17170	14936	1.00	1.05	0.83	0.94	0.82
	Mean						11063	12361	9651	11797	9750	1.00	1.15	0.95	1.17	0.92
	SD						6955	7000	5008	5481	5326		0.09	0.21	0.25	0.09
							Lane normalization ratio: actin intensity parental 231 cells (control)/actin intensity of POI lane)									
actin	1	12315	12552	12407	12763	12400	1.00	1.02	1.01	1.04	1.01					

	2	21633	21276	20487	22849	22994	1.00	0.98	0.95	1.06	1.06					
	3	40740	49975	42665	43339	49684	1.00	1.23	1.05	1.06	1.22					
p-Src (419)	1	10498	11059	8414	11152	10498	10498	11067	8118	10770	9629	1.00	1.05	0.77	1.03	0.92
(60 kDa)	2	23894	21877	20339	21476	23894	23894	20820	20564	20389	21425	1.00	0.87	0.86	0.85	0.90
	3	41299	47389	38828	43988	41299	41299	46380	38930	44970	41818	1.00	1.12	0.94	1.09	1.01
	Mean						25230	26089	22538	25376	24291	1.00	1.02	0.86	0.99	0.94
	SD						15444	18236	15500	17637	16284		0.13	0.08	0.12	0.06
							Lane normalization ratio: actin intensity parental 231 cells (control)/actin intensity of POI lane)									
actin	1	4388	4385	4548	4544	4784	1.00	1.00	1.04	1.04	1.09					
	2	11568	12155	11441	12184	12901	1.00	1.05	0.99	1.05	1.12					
	3	38989	39837	38886	38137	38505	1.00	1.02	1.00	0.98	0.99					
p-Src(527)	1	4027	3907	3969	4029	4027	4027	4003	4198	4338	4212	1.00	0.99	1.04	1.08	1.05
(60 kDa)	2	11885	14826	10483	11258	11885	11885	14754	10525	11556	11870	1.00	1.24	0.89	0.97	1.00
	3	23770	20865	18817	19973	23770	23770	21016	20902	22350	25401	1.00	0.88	0.88	0.94	1.07
	Mean						13227	13258	11875	12748	13828	1.00	1.04	0.94	1.00	1.04
	SD						9940	8605	8433	9065	10729		0.18	0.09	0.07	0.04
							Lane normalization ratio: actin intensity parental 231 cells (control)/actin intensity of POI lane)									
actin	1	10838	10578	10247	10066	10360	1.00	0.98	0.95	0.93	0.96					
	2	23011	23123	22919	22419	23041	1.00	1.00	1.00	0.97	1.00					
	3	6654	6607	5991	5947	6227	1.00	0.99	0.90	0.89	0.94					

Src	1	7669	7169	6563	8326	7669	7669	7034	6514	8033	7616	1.00	0.92	0.85	1.05	0.99
(60 kDa)	2	18192	19405	19069	17059	18192	18192	19730	20137	16152	17115	1.00	1.08	1.11	0.89	0.94
	3	41118	47188	42345	41148	41118	41118	38468	40434	38680	33716	1.00	0.94	0.98	0.94	0.82
	Mean						22326	21744	22362	20955	19482	1.00	0.98	0.98	0.96	0.92
	SD						17104	15813	17069	15878	13210		0.09	0.13	0.08	0.09
							Lane normalization ratio: actin intensity parental 231 cells (control)/actin intensity of POI lane									
actin	1	12315	12552	12407	12763	12400	1.00	1.02	1.01	1.04	1.01					
	2	21633	21276	20487	22849	22994	1.00	0.98	0.95	1.06	1.06					
	3	40740	49975	42665	43339	49684	1.00	1.23	1.05	1.06	1.22					
p-FAK	1	15586	13549	13210	13372	0	15586	14299	13147	15491	0	1.00	0.92	0.84	0.99	0.00
(125 kDa)	2	34226	30840	29038	28972	20137	34226	29524	29135	27998	19863	1.00	0.86	0.85	0.82	0.58
	3	38576	81242	32346	3834	32841	38576	79021	32000	3832	32571	1.00	2.05	0.83	0.10	0.84
	Mean						29463	40948	24761	15774	17478	1.00	1.28	0.84	0.64	0.47
	SD						12213	33840	10159	12086	16416		0.67	0.01	0.47	0.43
							Lane normalization ratio: actin intensity parental 231 cells (control)/actin intensity of POI lane									
actin	1	5848	5541	5876	5048	5386	1.00	0.95	1.00	0.86	0.92					
	2	12177	12719	12136	12600	12345	1.00	1.04	1.00	1.03	1.01					
	3	21109	21702	21337	21119	21284	1.00	1.03	1.01	1.00	1.01					
FAK	1	7144	6782	3905	8003	7144	7144	6654	3876	7722	7095	1.00	0.93	0.54	1.08	0.99
(125 kDa)	2	16681	17218	19837	16820	16681	16681	17507	20947	15926	15694	1.00	1.05	1.26	0.95	0.94

	3	12413	17061	14964	10767	12413	12413	13908	14288	10121	10178	1.00	1.12	1.15	0.82	0.82
	Mean						12079	12690	13037	11256	10989	1.00	1.03	0.98	0.95	0.92
	SD						4778	5528	8604	4218	4357		0.10	0.39	0.13	0.09
							Lane normalization ratio: actin intensity parental 231 cells (control)/actin intensity of POI lane)									
actin	1	12315	12552	12407	12763	12400	1.00	1.02	1.01	1.04	1.01					
	2	21633	21276	20487	22849	22994	1.00	0.98	0.95	1.06	1.06					
	3	40740	49975	42665	43339	49684	1.00	1.23	1.05	1.06	1.22					
CD44	1	5849	5842	5533	5670	5849	5849	6165	5507	6568	6352	1.00	1.05	0.94	1.12	1.09
(80 kDa)	2	15264	19488	13716	15203	15264	15264	18656	13761	14693	15056	1.00	1.22	0.90	0.96	0.99
	3	20142	29079	20078	14747	20142	20142	28284	19863	14740	19977	1.00	1.40	0.99	0.73	0.99
	Mean						13752	17702	13044	12000	13795	1.00	1.23	0.94	0.94	1.02
	SD				ss		7266	11090	7205	4704	6900		0.18	0.04	0.20	0.06
							Lane normalization ratio: actin intensity parental 231 cells (control)/actin intensity of POI lane)									
actin	1	5848	5541	5876	5048	5386	1.00	0.95	1.00	0.86	0.92					
	2	12177	12719	12136	12600	12345	1.00	1.04	1.00	1.03	1.01					
	3	21109	21702	21337	21119	21284	1.00	1.03	1.01	1.00	1.01					
Ezrin	1	5660	10008	9815	4962	5660	5660	10254	10381	5343	5921	1.00	1.81	1.83	0.94	1.05
(69 kDa)	2	14718	15992	12853	13827	14718	14718	15914	12904	14192	14699	1.00	1.08	0.88	0.96	1.00
	3	14427	15645	16664	10504	14427	14427	15758	18510	11754	15417	1.00	1.09	1.28	0.81	1.07
	Me						11602	13976	13932	10430	12012	1.00	1.33	1.33	0.91	1.04

	anSD						5148	3224	4161	4571	5287	0.42	0.48	0.08	0.04
							Lane normalization ratio: actin intensity parental 231 cells (control)/actin intensity of POI lane)								
actin	1	10838	10578	10247	10066	10360	1.00	0.98	0.95	0.93	0.96				
	2	23011	23123	22919	22419	23041	1.00	1.00	1.00	0.97	1.00				
	3	6654	6607	5991	5947	6227	1.00	0.99	0.90	0.89	0.94				

Table S21. Densitometry analysis comparing the expression of FABP-EET network markers in MDA-MB-231, LM6 and FABP4/FABP5CYP2C19-depleted cells (Figure 4f; cells co-cultured with THP-1 monocytes). Densitometry software (ImageJ) was used to determine the band intensities for each protein of interest (POI). Band intensities of the corresponding housekeeping protein (actin) were used as loading control (LC) for each lane. Normalized protein intensity for each POI was calculated by multiplying the band intensity by the loading control normalization ratio (ratio of actin intensity in the parental 231 control lane to the actin intensity in the POI lane). Relative protein expression for each POI was calculated as fold change relative to the normalized protein intensity of parental 231 cells. Mean relative protein expressions and standard deviations for three independent western blotting experiments were obtained and statistical analysis was performed using one-way ANOVA, *post hoc* Dunnett's test, with protein expression of the parental 231 cells as control. Red color indicates $p < 0.05$.

Protein/Experiment		Protein/Band Densitometry Intensity from ImageJ					Normalized Protein Intensity (POI Intensity*Lane Normalization Ratio)					Relative Protein Expression (Fold Change Relative to Expression in 231 Cells)				
		231	LM6-shLacZ	LM6-shFABP4	LM6-shFABP5	LM6-shCYP2C19	231	LM6-shLacZ	LM6-shFABP4	LM6-shFABP5	LM6-shCYP2C19	231	LM6-shLacZ	LM6-shFABP4	LM6-shFABP5	LM6-shCYP2C19
CD36	1	8047	12424	8077	5012	4928	8047	12471	8669	5017	4906	1.00	1.55	1.08	0.62	0.61
(85 kDa)	2	18892	17138	19207	12842	13515	18892	17133	19336	12650	13418	1.00	0.91	1.02	0.67	0.71
	3	5951	9283	4203	3571	4115	5951	9541	4147	3688	3958	1.00	1.60	0.70	0.62	0.67
	Mean						10963	13049	10718	7118	7427	1.00	1.35	0.93	0.64	0.66
	SD						6946	3829	7799	4836	5210		0.39	0.21	0.03	0.05
							Lane normalization ratio: actin intensity parental 231 cells (control)/actin intensity of POI lane)									
actin	1	8673	8640	8080	8664	8711	1.00	1.00	0.93	1.00	1.00					
	2	21122	21127	20981	21442	21275	1.00	1.00	0.99	1.02	1.01					
	3	11497	11186	11651	11130	11952	1.00	0.97	1.01	0.97	1.04					
c-myc	1	3826	4490	2541	5486	3853	3826	4213	2366	5700	4088	1.00	1.10	0.62	1.49	1.07
(57 kDa)	2	11034	12304	7218	15751	9846	11034	12411	7407	15484	10053	1.00	1.12	0.67	1.40	0.91
	3	13691	13852	11056	18897	8692	13691	13842	11133	18841	8622	1.00	1.01	0.81	1.38	0.63
	Mean						9517	10155	6969	13342	7588	1.00	1.08	0.70	1.42	0.87
	SD						5105	5196	4400	6827	3115		0.06	0.10	0.06	0.22
							Lane normalization ratio: actin intensity parental 231 cells (control)/actin intensity of POI lane)									
actin	1	4320	4604	4639	4158	4073	1.00	1.07	1.07	0.96	0.94					

	2	10041	9954	9785	10214	9834	1.00	0.99	0.97	1.02	0.98					
	3	21160	21176	21013	21222	21331	1.00	1.00	0.99	1.00	1.01					
Sox-2	1	5906	6240	5942	7987	5831	5906	6046	5923	7797	5362	1.00	1.02	1.00	1.32	0.91
(34 kDa)	2	15603	14697	15574	16093	15372	15603	14466	15587	15891	15331	1.00	0.93	1.00	1.02	0.98
	3	1782	1718	1921	2693	1091	1782	1683	1895	2775	1095	1.00	0.94	1.06	1.56	0.61
	Mean						7764	7398	7801	8821	7262	1.00	0.97	1.02	1.30	0.83
	SD						7096	6498	7037	6618	7306		0.05	0.04	0.27	0.19
							Lane normalization ratio: actin intensity parental 231 cells (control)/actin intensity of POI lane)									
actin	1	10081	10406	10115	10328	10962	1.00	1.03	1.00	1.02	1.09					
	2	21431	21772	21413	21703	21489	1.00	1.02	1.00	1.01	1.00					
	3	5791	5911	5873	5621	5772	1.00	1.02	1.01	0.97	1.00					
vimentin	1	5250	5256	6351	7146	5270	5250	5136	6804	7039	5265	1.00	0.98	1.30	1.34	1.00
(57 kDa)	2	13974	14328	11537	15499	13310	13974	13682	10559	15657	12286	1.00	0.98	0.76	1.12	0.88
	3	3292	3705	3029	7166	3105	3292	3754	2940	7143	3095	1.00	1.14	0.89	2.17	0.94
	Mean						7505	7524	6768	9946	6882	1.00	1.03	0.98	1.54	0.94
	SD						5687	5378	3810	4946	4804		0.09	0.28	0.55	0.06
							Lane normalization ratio: actin intensity parental 231 cells (control)/actin intensity of POI lane)									
actin	1	10743	10995	10028	10907	10753	1.00	1.02	0.93	1.02	1.00					
	2	4267	4469	4663	4225	4623	1.00	1.05	1.09	0.99	1.08					
	3	23055	22754	23752	23129	23130	1.00	0.99	1.03	1.00	1.00					
p-Src (419)	1	5250	5256	3351	7146	5270	5250	4932	3121	7424	5591	1.00	0.94	0.59	1.41	1.06
(60 kDa)	2	13974	14328	11537	15499	13310	13974	14452	11839	15236	13590	1.00	1.03	0.85	1.09	0.97

	3	3292	3705	3029	7166	3105	3292	3702	3050	7145	3080	1.00	1.12	0.93	2.17	0.94
	Mean						7505	7695	6003	9935	7420	1.00	1.03	0.79	1.56	0.99
	SD						5687	5884	5054	4593	5489		0.09	0.17	0.55	0.07
							Lane normalization ratio: actin intensity parental 231 cells (control)/actin intensity of POI lane)									
actin	1	4320	4604	4639	4158	4073	1.00	1.07	1.07	0.96	0.94					
	2	10041	9954	9785	10214	9834	1.00	0.99	0.97	1.02	0.98					
	3	21160	21176	21013	21222	21331	1.00	1.00	0.99	1.00	1.01					
p-Src(527) (60 kDa)	1	4628	5440	4687	4537	4575	4628	5270	4672	4429	4207	1.00	1.14	1.01	0.96	0.91
	2	11664	14736	13115	11830	12803	11664	14505	13126	11682	12769	1.00	1.24	1.13	1.00	1.09
	3	8625	7429	12461	6665	15176	8625	7278	12287	6867	15226	1.00	0.84	1.42	0.80	1.77
	Mean						8306	9018	10028	7659	10734	1.00	1.08	1.19	0.92	1.26
	SD						3529	4857	4658	3691	5784		0.21	0.21	0.11	0.45
							Lane normalization ratio: actin intensity parental 231 cells (control)/actin intensity of POI lane)									
actin	1	10081	10406	10115	10328	10962	1.00	1.03	1.00	1.02	1.09					
	2	21431	21772	21413	21703	21489	1.00	1.02	1.00	1.01	1.00					
	3	5791	5911	5873	5621	5772	1.00	1.02	1.01	0.97	1.00					
Src (60 kDa)	1	3094	4292	2984	3942	3118	3094	4194	3196	3883	3115	1.00	1.36	1.03	1.26	1.01
	2	8593	9552	9039	11361	10213	8593	9122	8273	11476	9427	1.00	1.06	0.96	1.34	1.10
	3	12546	13444	9726	6192	13011	12546	13622	9441	6172	12969	1.00	1.09	0.75	0.49	1.03
	Mean						8077	8979	6970	7177	8504	1.00	1.17	0.92	1.03	1.05
	SD						4747	4716	3320	3895	4991		0.16	0.15	0.47	0.05
							Lane normalization ratio: actin intensity parental 231 cells (control)/actin intensity of POI lane)									

actin	1	10743	10995	10028	10907	10753	1.00	1.02	0.93	1.02	1.00					
	2	4267	4469	4663	4225	4623	1.00	1.05	1.09	0.99	1.08					
	3	23055	22754	23752	23129	23130	1.00	0.99	1.03	1.00	1.00					
p-FAK	1	3842	3612	4033	3991	3668	3842	3739	3842	3941	3643	1.0	1.0	1.0	1.0	0.9
(125 kDa)	2	10490	8099	9287	15698	12178	10490	8670	8631	15393	11428	1.0	0.8	0.8	1.5	1.1
	3	4628	4177	4370	4904	3989	4628	4741	4628	3979	5612	1.0	1.0	1.0	0.9	1.2
	Mean						6320	5717	5700	7771	6895	1.00	0.94	0.94	1.12	1.08
	SD						3633	2606	2569	6601	4048		0.10	0.10	0.31	0.13
							Lane normalization ratio: actin intensity parental 231 cells (control)/actin intensity of POI lane)									
actin	1	3842	3712	4033	3891	3868	1.00	0.97	1.05	1.01	1.01					
	2	10490	9799	11287	10698	11178	1.00	0.93	1.08	1.02	1.07					
	3	4628	4077	4370	5704	3289	1.00	0.88	0.94	1.23	0.71					
FAK	1	4251	4991	4348	3840	4321	4251	4877	4658	3782	4317	1.00	1.15	1.10	0.89	1.02
(125 kDa)	2	11424	13725	11755	11562	11865	11424	13107	10759	11679	10952	1.00	1.15	0.94	1.02	0.96
	3	14049	12493	10695	20369	11138	14049	12658	10381	20304	11102	1.00	0.90	0.74	1.45	0.79
	Mean						9908	10214	8599	11922	8790	1.00	1.07	0.93	1.12	0.92
	SD						5072	4627	3419	8263	3875		0.14	0.18	0.29	0.12
							Lane normalization ratio: actin intensity parental 231 cells (control)/actin intensity of POI lane)									
actin	1	10743	10995	10028	10907	10753	1.00	1.02	0.93	1.02	1.00					
	2	4267	4469	4663	4225	4623	1.00	1.05	1.09	0.99	1.08					
	3	23055	22754	23752	23129	23130	1.00	0.99	1.03	1.00	1.00					
CD44	1	3125	3587	3383	2658	2543	3125	3601	3631	2661	2532	1.00	1.15	1.16	0.85	0.81

(80 kDa)	2	9936	13308	10433	7468	8430	9936	13305	10503	7356	8369	1.00	1.34	1.06	0.74	0.84
	3	9042	9057	6513	4021	4313	9042	9309	6427	4154	4149	1.00	1.03	0.71	0.46	0.46
	Mean						7368	8738	6854	4724	5017	1.00	1.17	0.98	0.68	0.70
	SD						3701	4877	3456	2399	3014		0.16	0.24	0.20	0.21
							Lane normalization ratio: actin intensity parental 231 cells (control)/actin intensity of POI lane)									
actin	1	8673	8640	8080	8664	8711	1.00	1.00	0.93	1.00	1.00					
	2	21122	21127	20981	21442	21275	1.00	1.00	0.99	1.02	1.01					
	3	11497	11186	11651	11130	11952	1.00	0.97	1.01	0.97	1.04					
Ezrin	1	4398	5083	4279	4291	4444	4398	4935	3929	4896	4481	1.00	1.12	0.89	1.11	1.02
(69 kDa)	2	11274	13690	11230	11477	11583	11274	12785	10389	11186	10604	1.00	1.13	0.92	0.99	0.94
	3	18334	30291	17861	5699	24125	18334	29197	17609	5521	23423	1.00	1.59	0.96	0.30	1.28
	Mean						11335	15639	10642	7201	12836	1.00	1.28	0.93	0.80	1.08
	SD						6968	12380	6844	3465	9666		0.27	0.03	0.44	0.18
							Lane normalization ratio: actin intensity parental 231 cells (control)/actin intensity of POI lane)									
actin	1	3565	3672	3883	3125	3536	1.00	1.03	1.09	0.88	0.99					
	2	8191	8771	8854	8404	8947	1.00	1.07	1.08	1.03	1.09					
	3	28127	29181	28531	29032	28969	1.00	1.04	1.01	1.03	1.03					

Table S22. Densitometry analysis of effects of dLGG (40 μM) on the expression levels of FABP-EET network proteins in LM6 cells. (Figure 7a). Densitometry software (ImageJ) was used to determine the band intensities for each protein of interest (POI). Band intensities of the corresponding housekeeping protein (actin) were used as loading control (LC) for each lane. Normalized protein intensity for each POI was calculated by multiplying the band intensity by the loading control normalization ratio (ratio of actin intensity in the vehicle-treated control lane to the actin intensity in the POI lane). Relative protein expression for each POI was calculated as fold change relative to the normalized protein intensity in the parental 231 cells. Mean relative protein expressions and standard deviations for three independent western blotting experiments were obtained and statistical analysis was performed using one-way ANOVA, *post hoc* Dunnett's test, with protein expression of vehicle-treated cells as control. Red color indicates $p < 0.05$.

**Protein/Experiment**		**Protein/band densitometry intensity from ImageJ**		**Normalized protein intensity (POI intensity*lane normalization ratio)**		**Relative protein expression (Fold change relative to expression in 231 cells)**	
		231	LM6-shLacZ	231	LM6-shLacZ	231	LM6-shLacZ
FABP4	1	10853	6705.57	10854	6973	1.00	0.64
(15 kDa)	2	3622	184.22	3628	153	1.00	0.04
	3	18046	12423.85	18047	10570	1.00	0.59
	Mean			10843	5899	1.00	0.42
	SD			7209	5291		0.33
				Lane normalization ratio: actin intensity vehicle-treated cells (control)/actin intensity (POI lane)			
actin	1	8602	8271.94	1.00	0.96		
	2	1032	1239.35	1.00	1.20		
	3	4628	5439.94	1.00	1.18		
FABP5	1	5460	3896.59	5460	3782	1.00	0.69
(15 kDa)	2	1989	1111.42	1990	1144	1.00	0.58
	3	3825	3489.87	3826	3440	1.00	0.90
	Mean			3759	2789	1.00	0.72
	SD			1736	1434		0.16
				Lane normalization ratio: actin intensity vehicle-treated cells (control)/actin intensity (POI lane)			
actin	1	9516	9804	1.00	1.03		
	2	3175	3084	1.00	0.97		
	3	15713	15941	1.00	1.01		
CYP2C19	1	8420	7846.76	8420	8160	1.00	0.97
(54 kDa)	2	1183	843.66	1184	703	1.00	0.59
	3	5905	6240.36	5906	5309	1.00	0.90
	Mean			5170	4724	1.00	0.82
	SD			3674	3763		0.20
				Lane normalization ratio: actin intensity vehicle-treated cells (control)/actin intensity (POI lane)			
actin	1	8602	8271.94	1.00	0.96		
	2	1032	1239.35	1.00	1.20		

	3	4628	5439.94	1.00	1.18		
PPAR-γ	1	6919	4125.41	6920	4004	1.00	0.58
(57 kDa)	2	239	203.6	240	210	1.00	0.87
	3	5250	5255.85	5250	5181	1.00	0.99
	Mean			4136	3132	1.00	0.81
	SD			3476	2598		
				Lane normalization ratio: actin intensity vehicle-treated cells (control)/actin intensity (POI lane)			
actin	1	9516	9804	1.00	1.03		
	2	3175	3084	1.00	0.97		
	3	15713	15941	1.00	1.01		
SREBP-2	1	10034	5863	10034	5628.48	1.00	1.06
(60 kDa)	2	3004	777	3004	932.4	1.00	0.92
	3	7068	3885	7068	4584.3	1.00	0.72
	Mean			6702	3715.06	1.00	0.90
	SD			10034	5628.48		0.17
				Lane normalization ratio: actin intensity vehicle-treated cells (control)/actin intensity (POI lane)			
actin	1	8602.06	8271.94	1.00	0.96		
	2	1032.07	1239.35	1.00	1.20		
	3	4628.16	5439.94	1.00	1.18		

Table S23. Statistical analysis performed for the comparison of the protein expressions of FABP4, FABP5, CYP2C19 and related metastasis-associated markers in parental MDA-MB-231 (231) and MDA-MB-231 with luciferase and fluorescence reporter genes (231) and lung-seeking metastatic subclones of iR2L (LM1 to LM6) (Figure 2b). Mean protein expressions (as fold change relative to the parental 231 cells) for the three experiments were compared using one-way ANOVA, *post hoc* Dunnett's test, with protein expression of the parental 231 cells as control; $P < 0.05$.

**Multiple Comparisons (one-way ANOVA)**							
**Dunnett t (2-sided)**							
**Dependent Variable**	**(I)**	**(J)**	**Mean Difference (I-J)**	**Std. Error**	**Sig.**	**95% Confidence Interval**	
						Lower Bound	Lower Bound
CYP2C19	2	1	0.3651	0.2275	0.464	-0.3001	1.0303
	3	1	-0.27537	0.2275	0.723	-0.9405	0.3898
	4	1	-0.13003	0.2275	0.989	-0.7952	0.5351
	5	1	0.08352	0.2275	0.999	-0.5816	0.7487
	6	1	.83616*	0.2275	0.011	0.171	1.5013
	7	1	.75738*	0.2275	0.022	0.0922	1.4225
	8	1	1.00687*	0.2275	0.002	0.3417	1.672
FABP4	2	1	-0.23927	0.13573	0.373	-0.6361	0.1576
	3	1	-0.09817	0.13573	0.962	-0.495	0.2987
	4	1	-0.32927	0.13573	0.127	-0.7261	0.0676
	5	1	-0.19643	0.13573	0.565	-0.5933	0.2004
	6	1	0.08649	0.13573	0.98	-0.3103	0.4833
	7	1	-0.33707	0.13573	0.114	-0.7339	0.0598
	8	1	0.04291	0.13573	1	-0.3539	0.4397
FABP5	2	1	0.29337	0.20865	0.592	-0.3167	0.9034
	3	1	-0.08889	0.20865	0.998	-0.6989	0.5212
	4	1	0.56374	0.20865	0.076	-0.0463	1.1738
	5	1	.80771*	0.20865	0.008	0.1977	1.4178
	6	1	.76255*	0.20865	0.012	0.1525	1.3726
	7	1	.90975*	0.20865	0.003	0.2997	1.5198
	8	1	1.18705*	0.20865	0	0.577	1.7971
Vimentin	2	1	-0.08649	0.20496	0.998	-0.6858	0.5128
	3	1	-0.0904	0.20496	0.997	-0.6897	0.5089
	4	1	-0.03239	0.20496	1	-0.6316	0.5669
	5	1	-0.13368	0.20496	0.977	-0.7329	0.4656
	6	1	-0.01029	0.20496	1	-0.6096	0.589
	7	1	-0.09459	0.20496	0.997	-0.6939	0.5047
	8	1	-0.04444	0.20496	1	-0.6437	0.5548
RhoA	2	1	0.05348	0.21848	1	-0.5853	0.6923
	3	1	-0.05825	0.21848	1	-0.6971	0.5806
	4	1	-0.01152	0.21848	1	-0.6503	0.6273
	5	1	0.03182	0.21848	1	-0.607	0.6706
	6	1	.95888*	0.21848	0.003	0.3201	1.5977
	7	1	0.59468	0.21848	0.074	-0.0441	1.2335

	8	1	1.03909*	0.21848	0.001	0.4003	1.6779
p-Src (419)	2	1	-0.05012	0.20312	1	-0.644	0.5438
	3	1	-0.12454	0.20312	0.983	-0.7184	0.4693
	4	1	0.04938	0.20312	1	-0.5445	0.6433
	5	1	-0.02434	0.20312	1	-0.6182	0.5695
	6	1	-0.08539	0.20312	0.998	-0.6793	0.5085
	7	1	-0.02557	0.20312	1	-0.6195	0.5683
	8	1	-0.30544	0.20312	0.528	-0.8993	0.2884
p-Src (527)	2	1	-0.11923	0.28154	0.998	-0.9424	0.7039
	3	1	0.02395	0.28154	1	-0.7992	0.8471
	4	1	0.08732	0.28154	1	-0.7359	0.9105
	5	1	-0.0595	0.28154	1	-0.8827	0.7637
	6	1	0.09421	0.28154	1	-0.729	0.9174
	7	1	.84210*	0.28154	0.044	0.0189	1.6653
	8	1	1.10419*	0.28154	0.007	0.281	1.9274
Src	2	1	0.35866	0.26576	0.63	-0.4184	1.1357
	3	1	0.15713	0.26576	0.986	-0.6199	0.9342
	4	1	0.44819	0.26576	0.416	-0.3288	1.2252
	5	1	0.28671	0.26576	0.806	-0.4903	1.0637
	6	1	0.51819	0.26576	0.282	-0.2588	1.2952
	7	1	0.6847	0.26576	0.096	-0.0923	1.4617
	8	1	0.75748	0.26576	0.058	-0.0196	1.5345
p-FAK	2	1	0.0575	0.26713	1	-0.7235	0.8385
	3	1	-0.06293	0.26713	1	-0.844	0.7181
	4	1	-0.13391	0.26713	0.995	-0.9149	0.6471
	5	1	-0.03906	0.26713	1	-0.8201	0.742
	6	1	0.67112	0.26713	0.108	-0.1099	1.4522
	7	1	0.49391	0.26713	0.329	-0.2871	1.2749
	8	1	0.52269	0.26713	0.279	-0.2584	1.3037
FAK	2	1	0.06174	0.14189	0.998	-0.3531	0.4766
	3	1	0.06576	0.14189	0.997	-0.3491	0.4806
	4	1	-0.05039	0.14189	0.999	-0.4652	0.3645
	5	1	0.23727	0.14189	0.424	-0.1776	0.6521
	6	1	-0.02843	0.14189	1	-0.4433	0.3864
	7	1	0.07987	0.14189	0.99	-0.335	0.4947
	8	1	0.194	0.14189	0.618	-0.2209	0.6088
MMP9	2	1	0.50994	0.22082	0.156	-0.1357	1.1556
	3	1	0.12493	0.22082	0.989	-0.5207	0.7706
	4	1	0.43062	0.22082	0.282	-0.215	1.0763
	5	1	0.21176	0.22082	0.873	-0.4339	0.8574
	6	1	.73731*	0.22082	0.022	0.0917	1.3829
	7	1	0.30948	0.22082	0.595	-0.3362	0.9551
	8	1	0.51393	0.22082	0.151	-0.1317	1.1596
Ezrin	2	1	0.20301	0.41881	0.996	-1.0215	1.4275
	3	1	0.11656	0.41881	1	-1.108	1.3411

	4	1	1.16819	0.41881	0.065	-0.0563	2.3927
	5	1	1.04031	0.41881	0.114	-0.1842	2.2648
	6	1	1.32808*	0.41881	0.031	0.1035	2.5526
	7	1	1.49593*	0.41881	0.014	0.2714	2.7205
	8	1	1.17294	0.41881	0.063	-0.0516	2.3975
	2	1	0.3651	0.2275	0.464	-0.3001	1.0303
	3	1	-0.27537	0.2275	0.723	-0.9405	0.3898
	4	1	-0.13003	0.2275	0.989	-0.7952	0.5351
CD44	5	1	0.08352	0.2275	0.999	-0.5816	0.7487
	6	1	.83616*	0.2275	0.011	0.171	1.5013
	7	1	.75738*	0.2275	0.022	0.0922	1.4225
	8	1	1.00687*	0.2275	0.002	0.3417	1.672

*. The mean difference is significant at the 0.05 level.

Table S24. Statistical analysis performed for the comparison the changes in protein expression of metastasis-associated protein markers after shRNA-mediated knockdown of FABP4, FABP5, CYP2C19 in highly lung-seeking TNBC subclone (LM6) (Figure 2c). Mean protein expressions (as fold change relative to the parental 231 cells) for the three experiments were compared using one-way ANOVA, *post hoc* Dunnett's test, with protein expression of the parental 231 cells as control; $p < 0.05$.

Multiple Comparisons (one-way ANOVA)							
Dunnett t (2-sided)							
Dependent Variable	(I)	(J)	Mean Difference (I-J)	Std. Error	Sig.	95% Confidence Interval	
						Lower Bound	Upper Bound
CYP2C19	2	1	*-0.07123*	*0.26317*	*1*	*-0.8407*	*0.6982*
	3	1	0.27614	0.26317	0.824	-0.4933	1.0456
	4	1	-0.61127	0.26317	0.152	-1.3807	0.1582
	5	1	-0.52198	0.26317	0.268	-1.2914	0.2475
	6	1	-0.21297	0.26317	0.936	-0.9824	0.5565
	7	1	-0.15836	0.26317	0.985	-0.9278	0.6111
	8	1	*-0.18975*	*0.26317*	*0.963*	*-0.9592*	*0.5797*
FABP4	2	1	0.13067	0.06804	0.295	-0.0683	0.3296
	3	1	-0.09571	0.06804	0.592	-0.2946	0.1032
	4	1	-.53381*	0.06804	0	-0.7327	-0.3349
	5	1	-.63325*	0.06804	0	-0.8322	-0.4343
	6	1	-.32718*	0.06804	0.001	-0.5261	-0.1283
	7	1	*-.26362**	*0.06804*	*0.007*	*-0.4625*	*-0.0647*
	8	1	-.20081*	0.06804	0.047	-0.3997	-0.0019
FABP5	2	1	0.36531	0.2215	0.437	-0.2823	1.0129
	3	1	0.19097	0.2215	0.917	-0.4567	0.8386
	4	1	0.13115	0.2215	0.986	-0.5165	0.7788
	5	1	0.30934	0.2215	0.598	-0.3383	0.957
	6	1	*-0.02809*	*0.2215*	*1*	*-0.6757*	*0.6195*
	7	1	0.10978	0.2215	0.995	-0.5378	0.7574
	8	1	0.08183	0.2215	0.999	-0.5658	0.7294
Vimentin	2	1	-.36306*	0.09915	0.012	-0.653	-0.0732
	3	1	-.29210*	0.09915	0.048	-0.582	-0.0022
	4	1	-.74409*	0.09915	0	-1.034	-0.4542
	5	1	-.70372*	0.09915	0	-0.9936	-0.4138
	6	1	*-.69236**	*0.09915*	*0*	*-0.9823*	*-0.4025*
	7	1	-.52143*	0.09915	0	-0.8113	-0.2315
	8	1	-.42214*	0.09915	0.003	-0.7121	-0.1322
RhoA	2	1	0.49005	0.27343	0.358	-0.3094	1.2895
	3	1	0.51446	0.27343	0.313	-0.285	1.3139
	4	1	-0.25089	0.27343	0.893	-1.0503	0.5486
	5	1	*0.41333*	*0.27343*	*0.523*	*-0.3861*	*1.2128*
	6	1	*0.03203*	*0.27343*	*1*	*-0.7674*	*0.8315*
	7	1	-0.3405	0.27343	0.7	-1.14	0.459

	8	1	0.1321	0.27343	0.996	-0.6674	0.9315
p-Src (419)	2	1	-0.21911	0.09875	0.182	-0.5078	0.0696
	3	1	0.19345	0.09875	0.278	-0.0953	0.4822
	4	1	*-0.19634*	*0.09875*	*0.266*	*-0.4851*	*0.0924*
	5	1	.29006*	0.09875	0.049	0.0013	0.5788
	6	1	-.42732*	0.09875	0.003	-0.716	-0.1386
	7	1	0.1268	0.09875	0.674	-0.1619	0.4155
	8	1	-0.22685	0.09875	0.159	-0.5156	0.0619
p-Src (527)	2	1	0.11471	0.12168	0.881	-0.2411	0.4705
	3	1	*-0.28572*	*0.12168*	*0.145*	*-0.6415*	*0.07*
	4	1	-0.20775	0.12168	0.404	-0.5635	0.148
	5	1	-0.09284	0.12168	0.951	-0.4486	0.2629
	6	1	0.04331	0.12168	0.999	-0.3125	0.3991
	7	1	-0.27549	0.12168	0.168	-0.6313	0.0803
	8	1	0.28789	0.12168	0.141	-0.0679	0.6437
p-Src	2	1	0.32063	0.23042	0.602	-0.3531	0.9943
	3	1	0.17167	0.23042	0.956	-0.502	0.8454
	4	1	0.52479	0.23042	0.164	-0.1489	1.1985
	5	1	0.34985	0.23042	0.518	-0.3238	1.0235
	6	1	0.05128	0.23042	1	-0.6224	0.725
	7	1	-0.4799	0.23042	0.228	-1.1536	0.1938
	8	1	-0.09216	0.23042	0.999	-0.7659	0.5815
Src	2	1	0.11646	0.19519	0.986	-0.4543	0.6872
	3	1	-0.09455	0.19519	0.996	-0.6653	0.4762
	4	1	-0.26165	0.19519	0.636	-0.8324	0.3091
	5	1	-0.06725	0.19519	0.999	-0.638	0.5035
	6	1	-0.26683	0.19519	0.618	-0.8375	0.3039
	7	1	-0.31329	0.19519	0.464	-0.884	0.2574
	8	1	-0.02715	0.19519	1	-0.5979	0.5436
p-FAK	2	1	0.18472	0.18656	0.857	-0.3608	0.7302
	3	1	0.24499	0.18656	0.655	-0.3005	0.7905
	4	1	-0.21828	0.18656	0.75	-0.7638	0.3272
	5	1	0.2505	0.18656	0.635	-0.295	0.796
	6	1	0.22513	0.18656	0.726	-0.3203	0.7706
	7	1	0.34748	0.18656	0.323	-0.198	0.893
	8	1	0.42287	0.18656	0.168	-0.1226	0.9683
FAK	2	1	-0.05537	0.07458	0.957	-0.2734	0.1627
	3	1	-0.13554	0.07458	0.345	-0.3536	0.0825
	4	1	-.49049*	0.07458	0	-0.7085	-0.2724
	5	1	-.31207*	0.07458	0.004	-0.5301	-0.094
	6	1	-.37010*	0.07458	0.001	-0.5882	-0.152
	7	1	-0.14858	0.07458	0.264	-0.3666	0.0695
	8	1	.22610*	0.07458	0.041	0.008	0.4442
Ezrin	2	1	0.12496	0.1428	0.911	-0.2926	0.5425
	3	1	0.10586	0.1428	0.957	-0.3117	0.5234

	4	1	0.03841	0.1428	1	-0.3791	0.4559
	5	1	-.70314*	0.1428	0.001	-1.1207	-0.2856
	6	1	-0.05085	0.1428	0.999	-0.4684	0.3667
	7	1	.44030*	0.1428	0.037	0.0228	0.8578
	8	1	.63571*	0.1428	0.002	0.2182	1.0532
CD44	2	1	*-0.07123*	*0.26317*	*1*	*-0.8407*	*0.6982*
	3	1	0.27614	0.26317	0.824	-0.4933	1.0456
	4	1	-0.61127	0.26317	0.152	-1.3807	0.1582
	5	1	-0.52198	0.26317	0.268	-1.2914	0.2475
	6	1	-0.21297	0.26317	0.936	-0.9824	0.5565
	7	1	-0.15836	0.26317	0.985	-0.9278	0.6111
	8	1	*-0.18975*	*0.26317*	*0.963*	*-0.9592*	*0.5797*

*. The mean difference is significant at the 0.05 level.

Table S25. Statistical analysis performed for the comparison of the cellular localization of FABP4 and FABP5, and of lipogenic transcription factors PPAR-γ, SREBP-2, RAR and RXR-α in vehicle-treated or EET-supplemented LM6 cells (**Figure 3e)**. Mean protein expressions (as fold change relative to the corresponding cellular fraction of vehicle-treated LM6 cells) for the three experiments were compared using student's t-test, with protein expression of corresponding vehicle-treated cells as control; $p < 0.05$.

t-test for Independent Samples										
	**Levene's Test for Equality of Variances**					***t*-test for Equality of Means**				
			**Sig.**	**t**	**df**	**Sig. (2-tailed)**	**Mean Difference**	**Std. Error Difference**	**95% Confidence Interval of the Difference**	
									Lower	Upper
CYP2C19			Equal variances assumed					0.11791	-0.5593	0.095448
	Equal variances not assumed			-1.96695	3.536459	0.129825	0.129825	-0.23192	-2.03888	-0.11446
FABP4	Equal variances assumed	9.123486	0.039144	0.001455	0.001455	-1.78002	-0.56	0.138564	-0.94472	-0.17528
	Equal variances not assumed			-7.80312	2.418163	0.009048	0.009048	-1.78002	-1.15619	0.036193
FABP5	Equal variances assumed	2.259536	0.207221	0.000248	0.000248	-2.06883	-0.01667	0.012019	-0.05004	0.016702
	Equal variances not assumed			2.176752	0.004696	0.004696	-2.06883	0.012019	-0.06838	0.035045
PPAR-γ	Equal variances assumed	4.017449	0.11554	0.001281	0.001281	-1.42973	-0.20667	0.534592	-1.69093	1.277599
	Equal variances not assumed			-8.06995	2.452467	0.007986	0.007986	-1.42973	-1.96481	1.551481
SREBP-2	Equal variances assumed	2.113049	0.219717	0.758507	0.758507	1.047679	-0.01	0.005774	-0.02603	0.00603
	Equal variances not assumed			0.329219	2.779336	0.765221	0.765221	1.047679	-0.03484	0.014841
RXRα	Equal variances assumed	6.372601	0.065051	0.43715	0.43715	0.323857	-0.33333	0.333333	-1.25882	0.592148
	Equal variances not assumed			0.862304	2.924994	0.453415	0.453415	0.323857	-1.76755	1.100884
RAR	Equal variances assumed	0.166157	0.704423	0.826873	0.826873	-0.00867	-0.64333	0.084525	-0.87801	-0.40865
	Equal variances not assumed			-0.23345	2.352839	0.83419	0.83419	-0.00867	-1.00701	-0.27965

Table S26. Statistical analysis performed for the comparison of the expression of downstream direct targets of PPAR-γ and SREBP-2 in LM6-LacZ, LM6-shFABP4 and LM6-shFABP5 cells grown with or without EET supplementation (Figure 3f). Mean protein expressions (as fold change relative to the LM6-shLacZ cells) for the three experiments were compared using student's t-test, with protein expression of corresponding vehicle-treated cells as control; $p < 0.05$.

	***t*-test for Independent Samples**									
	**Levene's Test for Equality of Variances**				***t*-test for Equality of Means**					
			**Sig.**	**t**	**df**	**Sig. (2-tailed)**	**Mean Difference**	**Std. Error Difference**	**95% Confidence Interval of the Difference**	
									Lower	Upper
CD36			Equal variances assumed					0.314322	-1.30663	0.438771
	Equal variances not assumed			-1.38052	2	0.301469	-0.43393	0.079512	-0.36053	0.093868
c-myc	Equal variances assumed	4.295618	0.106916	0.083604	-0.28451	0.523383	-0.03333	0.047726	-0.16584	0.099175
	Equal variances not assumed			-2.29274	2	0.148888	-0.28451	0.047726	-0.1768	0.110136
Ezrin	Equal variances assumed	6.203438	0.067436	0.220826	0.079337	0.25113	-0.16333	0.121838	-0.50161	0.174943
	Equal variances not assumed			2	0.284265	0.079337	-0.16333	0.121838	-0.51032	0.183655
CD44	Equal variances assumed	15.95889	0.016199	0.027579	-0.51703	0.255582	0.076667	0.057831	-0.0839	0.237232
	Equal variances not assumed			-3.38806	2	0.077167	-0.51703	0.057831	-0.09129	0.244619
Src	Equal variances assumed	13.48199	0.021358	0.161695	0.077107	0.408917	-0.04333	0.047022	-0.17389	0.087222
	Equal variances not assumed			1.713929	2	0.228676	0.077107	0.047022	-0.17427	0.087599
FAK	Equal variances assumed	4.27642	0.107479	0.073261	-0.48097	0.168233	0.556667	0.331327	-0.36325	1.476579
	Equal variances not assumed			-2.4137	2	0.137191	-0.48097	0.331327	-0.86644	1.979769
PPAR-γ	Equal variances assumed	7.956	0.047797	0.147083	-0.30484	0.446812	-0.08333	0.098883	-0.35788	0.191209
	Equal variances not assumed			-1.79505	2	0.214493	-0.30484	0.098883	-0.35882	0.192152
SREBP-2	Equal variances assumed	6.844095	0.059039	0.799381	0.037823	0.288715	-0.09	0.073636	-0.29445	0.114446
	Equal variances not assumed			0.271586	2	0.811406	0.037823	0.073636	-0.29544	0.115437

Table S27. Statistical analysis performed for the comparison of the protein expression levels of adipocyte markers and intrinsic expression levels of FABP4, FABP5 and CYP2C19 in fibroblasts, adipocytes and monocytes grown in monoculture, with or without addition of 2 μM rosiglitazone (**Figure 4a)**. Mean protein expressions (as fold change relative to vehicle-treated cells) for the three experiments were compared using student's t-test, with protein expression of corresponding vehicle-treated cells as control; $p < 0.05$.

***t*-test for Independent Samples**										
	**Levene's Test for Equality of Variances**		***t*-test for Equality of Means**							
			**Sig.**	**t**	**df**	**Sig. (2-tailed)**	**Mean Difference**	**Std. Error Difference**	**95% Confidence Interval of the Difference**	
									Lower	Upper
C/EBPα			Equal variances assumed					0.37365	-1.61165	0.463188
	Equal variances not assumed			-1.53681	2	0.264151	-0.57423	0.013333	-0.04404	0.070702
PPAR-γ	Equal variances assumed	12.01318	0.025677	0.176422	-0.43716	0.016648	-0.66667	0.168259	-1.13383	-0.1995
	Equal variances not assumed			-1.63964	2	0.242756	-0.43716	0.168259	-1.39063	0.057294
FABP4	Equal variances assumed	11.32805	0.028155	0.029537	-0.91359	2.07E-07	-1.27	0.017321	-1.31809	-1.22191
	Equal variances not assumed			2	0.080236	-0.91359	-1.27	0.017321	-1.34452	-1.19548
FABP5	Equal variances assumed	14.77911	0.018391	0.000688	-1.91509	0.000567	-2.11333	0.211844	-2.70151	-1.52516
	Equal variances not assumed			-9.49128	2	0.010919	-1.91509	0.211844	-3.02482	-1.20184
CYP2C19	Equal variances assumed	4.570886	0.099315	0.420198	-0.35985	0.002175	-0.39333	0.056075	-0.54902	-0.23764
	Equal variances not assumed			-0.89748	2	0.464176	-0.35985	0.056075	-0.63461	-0.15206

Table S28. Statistical analysis performed for the comparison of the expression of FABP4, FABP4 and CYP2C19 in parental 231, LM6-shLacZ, LM6-shFABP4, LM6-shFABP5 and LM6-CYP2C19-depleted cells (Figure 4b; cells grown in monoculture). Mean protein expressions (as fold change relative to the parental 231 cells) for the three experiments were compared using one-way ANOVA, *post hoc* Dunnett's test, with protein expression of parental 231 cells as control; $p < 0.05$.

Multiple Comparisons (one-way ANOVA)							
Dunnett t (2-sided)							
**Dependent Variable**	**(I)**	**(J)**	**Mean Difference (I-J)**	**Std. Error**	**Sig.**	**95% Confidence Interval**	
						Upper Bound	Lower Bound
CYP2C19	2	1	0.28739	0.21646	0.512	-0.3383	0.9131
	3	1	-0.03278	0.21646	1	-0.6585	0.5929
	4	1	-0.39659	0.21646	0.263	-1.0223	0.2291
	5	1	-0.46245	0.21646	0.167	-1.0881	0.1632
FABP5	2	1	0.14918	0.20424	0.874	-0.4412	0.7395
	3	1	-0.37799	0.20424	0.256	-0.9684	0.2124
	4	1	-.59076*	0.20424	0.05	-1.1811	-0.0004
	5	1	-0.51728	0.20424	0.089	-1.1076	0.0731
FABP4	2	1	0.12568	0.17836	0.886	-0.3899	0.6412
	3	1	-0.44061	0.17836	0.099	-0.9562	0.0749
	4	1	-0.27501	0.17836	0.393	-0.7906	0.2405
	5	1	-0.49997	0.17836	0.058	-1.0155	0.0156

*. The mean difference is significant at the 0.05 level.

Table S29. Statistical analysis performed for the comparison of the expression of FABP4, FABP4 and CYP2C19 in parental 231, LM6-shLacZ, LM6-shFABP4, LM6-shFABP5 and LM6-CYP2C19-depleted cells (Figure 4b; cells grown in co-culture with adipocytes). Mean protein expressions (as fold change relative to the parental 231 cells) for the three experiments were compared using one-way ANOVA, *post hoc* Dunnett's test, with protein expression of parental 231 cells as control; $p < 0.05$.

Multiple Comparisons (one-way ANOVA)						
Dunnett t (>control)						
**Dependent Variable**	**(I)**	**(J)**	**Mean Difference (I-J)**	**Std. Error**	Sig.	95% Confidence Interval
						Lower Bound
CYP2C19	2	1	0.39744	0.1549	0.085	-0.0503
	3	1	0.09667	0.1549	0.921	-0.3511
	4	1	-0.12	0.1549	0.851	-0.5677
	5	1	-0.22667	0.1549	0.434	-0.6744
FABP5	2	1	0.53848	0.32676	0.341	-0.406
	3	1	0.15	0.32676	0.971	-0.7945
	4	1	-0.38333	0.32676	0.608	-1.3278
	5	1	-0.17667	0.32676	0.95	-1.1212
FABP4	2	1	0.29413	0.20859	0.464	-0.3088
	3	1	-0.52667	0.20859	0.091	-1.1296
	4	1	-0.21667	0.20859	0.694	-0.8196
	5	1	-0.44667	0.20859	0.166	-1.0496
*. The mean difference is significant at the 0.05 level.						

Table S30. Statistical analysis performed for the comparison of the expression of FABP4, FABP4 and CYP2C19 in parental 231, LM6-shLacZ, LM6-shFABP4, LM6-shFABP5 and LM6-CYP2C19-depleted cells (Figure 4b; cells grown in co-culture with fibroblasts). Mean protein expressions (as fold change relative to the parental 231 cells) for the three experiments were compared using one-way ANOVA, *post hoc* Dunnett's test, with protein expression of parental 231 cells as control; $p < 0.05$.

Multiple Comparisons (one-way ANOVA)						
Dunnett t (>control)						
**Dependent Variable**	**(I)**	**(J)**	**Mean Difference (I-J)**	**Std. Error**	Sig.	95% Confidence Interval
						Upper Bound
CYP2C19	2	1	0.13	0.322463	0.647733	-0.66506
	3	1	-0.12	0.322463	0.896782	-0.91506
	4	1	-0.13333	0.322463	0.904809	-0.92839
	5	1	-0.13333	0.322463	0.904809	-0.92839
FABP5	2	1	0.136667	0.186667	0.503222	-0.32358
	3	1	-0.49333	0.186667	0.999486	-0.95358
	4	1	-0.65333	0.186667	0.999921	-1.11358
	5	1	-0.65333	0.186667	0.999921	-1.11358
FABP4	2	1	-0.11333	0.313191	0.894708	-0.88553
	3	1	-0.37	0.313191	0.982625	-1.1422
	4	1	-0.22667	0.313191	0.950193	-0.99887
	5	1	-0.27	0.313191	0.963411	-1.0422

*. The mean difference is significant at the 0.05 level.

Table S31. Statistical analysis performed for the comparison of the expression of FABP4, FABP4 and CYP2C19 in parental 231, LM6-shLacZ, LM6-shFABP4, LM6-shFABP5 and LM6-CYP2C19-depleted cells (Figure 4b; cells grown in co-culture with THP-1 monocytes). Mean protein expressions (as fold change relative to the parental 231 cells) for the three experiments were compared using one-way ANOVA, *post hoc* Dunnett's test, with protein expression of parental 231 cells as control; $p < 0.05$.

Multiple Comparisons (one-way ANOVA)						
Dunnett t (>control)						
**Dependent Variable**	**(I)**	**(J)**	**Mean Difference (I-J)**	**Std. Error**	**Sig.**	**95% Confidence Interval**
						Upper Bound
CYP2C19	2	1	0.160	0.149	0.358	-0.21
	3	1	0.16667	0.14894	0.341	-0.20
	4	1	-0.02667	0.14894	0.852	-0.39
	5	1	-0.020	0.149	0.84	-0.39
FABP5	2	1	-0.087	0.253	0.89	-0.71
	3	1	-0.280	0.253	0.98	-0.90
	4	1	-0.707	0.253	1.00	-1.33
	5	1	-0.34	0.25295	0.99	-0.96
FABP4	2	1	0.10333	0.22173	0.62	-0.44
	3	1	-0.367	0.222	0.99	-0.91
	4	1	0.017	0.222	0.78	-0.53
	5	1	0.223	0.222	0.39	-0.32

*. The mean difference is significant at the 0.05 level.

Table S32. Statistical analysis performed for the comparison of the expression of FABP-EET network markers in MDA-MB-231, LM6 and FABP4/FABP5CYP2C19-depleted cells (Figure 4f; cells grown in monoculture). Mean protein expressions (as fold change relative to the parental 231 cells) for the three experiments were compared using one-way ANOVA, *post hoc* Dunnett's test, with protein expression of parental 231 cells as control; $p < 0.05$.

Multiple Comparisons							
Dunnett t (2-sided)							
**Dependent Variable**	**(I)**	**(J)**	**Mean Difference (I-J)**	**Std. Error**	**Sig.**	**95% Confidence Interval**	
						Lower Bound	Upper Bound
CD36	2	1	-0.31902	0.1153	0.061	-0.6523	0.0143
	3	1	-.57758*	0.1153	0.002	-0.9109	-0.2443
	4	1	-.42221*	0.1153	0.014	-0.71799	-0.7555
	5	1	-.48332*	0.1153	0.006	-0.8166	-0.15
c-myc	2	1	-0.32397	0.13787	0.12	-0.7225	0.0746
	3	1	-0.26734	0.13787	0.225	-0.6659	0.1312
	4	1	-0.32445	0.13787	0.119	-0.723	0.0741
	5	1	-0.22399	0.13787	0.352	-0.6225	0.1745
Sox-2	2	1	-0.09497	0.1265	0.864	-0.4606	0.2707
	3	1	-0.35615	0.1265	0.057	-0.7218	0.0095
	4	1	-.50158*	0.1265	0.009	-0.8672	-0.1359
	5	1	-0.32617	0.1265	0.083	-0.6918	0.0395
Vimentin	2	1	-.18979*	0.06448	0.046	-0.3762	-0.0034
	3	1	-.38037*	0.06448	0.001	-0.5667	-0.194
	4	1	-.35958*	0.06448	0.001	-0.546	-0.1732
	5	1	-.50359*	0.06448	0	-0.69	-0.3172
p-Src (419)	2	1	0.06947	0.21186	0.991	-0.5429	0.6818
	3	1	-0.23862	0.21186	0.638	-0.851	0.3737
	4	1	-0.25417	0.21186	0.591	-0.8665	0.3582
	5	1	-0.33712	0.21186	0.368	-0.9495	0.2753
p-Src (527)	2	1	-0.12903	0.19813	0.91	-0.7017	0.4437
	3	1	-.59082*	0.19813	0.043	-1.1635	-0.0181
	4	1	-.80433*	0.19813	0.008	-1.377	-0.2316
	5	1	-0.32287	0.19813	0.35	-0.8956	0.2498
Src	2	1	-0.00484	0.11571	1	-0.3393	0.3296
	3	1	-0.07563	0.11571	0.909	-0.4101	0.2588
	4	1	-0.12667	0.11571	0.658	-0.4611	0.2078
	5	1	-.38907*	0.11571	0.023	-0.7235	-0.0546
p-FAK	2	1	-.24885*	0.06255	0.009	-0.4296	-0.0681
	3	1	-0.12302	0.06255	0.216	-0.3038	0.0578
	4	1	-.30870*	0.06255	0.002	-0.4895	-0.1279
	5	1	-0.12865	0.06255	0.189	-0.3094	0.0521
FAK	2	1	-0.066	0.08557	0.853	-0.3133	0.1813
	3	1	-0.05823	0.08557	0.897	-0.3056	0.1891
	4	1	-0.1509	0.08557	0.29	-0.3982	0.0964
	5	1	-.27509*	0.08557	0.029	-0.5224	-0.0278

MMP9	2	1	-0.24386	0.16405	0.422	-0.718	0.2303
	3	1	-0.08773	0.16405	0.952	-0.5619	0.3864
	4	1	-0.40627	0.16405	0.098	-0.8804	0.0679
	5	1	-0.19879	0.16405	0.584	-0.673	0.2754
Ezrin	2	1	-0.23276	0.32883	0.885	-1.1832	0.7177
	3	1	-0.21253	0.32883	0.912	-1.163	0.7379
	4	1	0.05821	0.32883	0.999	-0.8923	1.0087
	5	1	0.1325	0.32883	0.982	-0.818	1.083
CD44	2	1	-0.31902	0.1153	0.061	-0.6523	0.0143
	3	1	-.57758*	0.1153	0.002	-0.9109	-0.2443
	4	1	-.42221*	0.1153	0.014	-0.7555	-0.0889
	5	1	-.48332*	0.1153	0.006	-0.8166	-0.15

*. The mean difference is significant at the 0.05 level.

Table S33. Statistical analysis performed for the comparison of the expression of FABP-EET network markers in MDA-MB-231, LM6 and FABP4/FABP5CYP2C19-depleted cells (Figure 4f; cells co-cultured with 3T3 fibroblasts). Mean protein expressions (as fold change relative to the parental 231 cells) for the three experiments were compared using one-way ANOVA, post hoc Dunnett's test, with protein expression of parental 231 cells as control; $p < 0.05$.

Multiple Comparisons (one-way ANOVA)							
Dunnett t (2-sided)							
**Dependent Variable**	**(I)**	**(J)**	**Mean Difference (I-J)**	**Std. Error**	**Sig.**	95% Confidence Interval	
						Lower Bound	Upper Bound
CD36	2	1	-0.33027	0.18314	0.274	-0.8596	0.1991
	3	1	-0.44907	0.18314	0.102	-0.9784	0.0803
	4	1	-.70869*	0.18314	0.01	-1.238	-0.1793
	5	1	-.55936*	0.18314	0.038	-1.0887	-0.03
c-myc	2	1	-0.35218	0.2067	0.316	-0.9496	0.2453
	3	1	-0.49646	0.2067	0.11	-1.0939	0.101
	4	1	-0.51108	0.2067	0.098	-1.1085	0.0864
	5	1	-0.42839	0.2067	0.184	-1.0258	0.1691
Sox-2	2	1	-0.32151	0.19061	0.323	-0.8725	0.2295
	3	1	-0.46968	0.19061	0.1	-1.0206	0.0813
	4	1	-0.34484	0.19061	0.272	-0.8958	0.2061
	5	1	-0.43078	0.19061	0.138	-0.9817	0.1202
Vimentin	2	1	-.37091*	0.10796	0.021	-0.683	-0.0589
	3	1	-0.21627	0.10796	0.205	-0.5283	0.0958
	4	1	-.50980*	0.10796	0.003	-0.8219	-0.1977
	5	1	-.45141*	0.10796	0.006	-0.7635	-0.1393
p-Src (419)	2	1	-0.17293	0.13995	0.569	-0.5774	0.2316
	3	1	-.51950*	0.13995	0.013	-0.924	-0.115
	4	1	-0.26289	0.13995	0.246	-0.6674	0.1416
	5	1	-0.36552	0.13995	0.079	-0.77	0.039
p-Src (527)	2	1	-0.18619	0.23723	0.846	-0.8719	0.4995
	3	1	-0.41023	0.23723	0.305	-1.0959	0.2755
	4	1	-0.34724	0.23723	0.434	-1.0329	0.3385
	5	1	-0.39066	0.23723	0.341	-1.0764	0.295
Src	2	1	-0.33709	0.16245	0.184	-0.8066	0.1325
	3	1	-0.38184	0.16245	0.12	-0.8514	0.0877
	4	1	-0.30524	0.16245	0.246	-0.7748	0.1643
	5	1	-0.19557	0.16245	0.589	-0.6651	0.274
p-FAK	2	1	-0.23041	0.1534	0.414	-0.6738	0.213
	3	1	-.49032*	0.1534	0.03	-0.9337	-0.0469
	4	1	-.72341*	0.1534	0.003	-1.1668	-0.28
	5	1	-.50984*	0.1534	0.025	-0.9532	-0.0664
FAK	2	1	-0.15976	0.16679	0.745	-0.6418	0.3223
	3	1	-0.2862	0.16679	0.31	-0.7683	0.1959
	4	1	-0.37677	0.16679	0.138	-0.8589	0.1053

	5	1	-0.07737	0.16679	0.97	-0.5595	0.4047
MMP9	2	1	0.05131	0.29145	0.999	-0.7911	0.8937
	3	1	-0.42429	0.29145	0.439	-1.2667	0.4181
	4	1	-0.44527	0.29145	0.4	-1.2877	0.3971
	5	1	-0.22435	0.29145	0.854	-1.0668	0.6181
Ezrin	2	1	-0.12165	0.26347	0.971	-0.8832	0.6399
	3	1	-0.47539	0.26347	0.274	-1.237	0.2862
	4	1	-0.37346	0.26347	0.46	-1.135	0.3881
	5	1	-0.54867	0.26347	0.181	-1.3102	0.2129
CD44	2	1	-0.33027	0.18314	0.274	-0.8596	0.1991
	3	1	-0.44907	0.18314	0.102	-0.9784	0.0803
	4	1	-.70869*	0.18314	0.01	-1.238	-0.1793
	5	1	-.55936*	0.18314	0.038	-1.0887	-0.03

*. The mean difference is significant at the 0.05 level.

Table S34. Statistical analysis performed for the comparison of the expression of FABP-EET network markers in MDA-MB-231, LM6 and FABP4/FABP5CYP2C19-depleted cells (Figure 4f; cells co-cultured with 3T3 differentiated adipocytes). Mean protein expressions (as fold change relative to the parental 231 cells) for the three experiments were compared using one-way ANOVA, *post hoc* Dunnett's test, with protein expression of parental 231 cells as control; $p < 0.05$.

Multiple Comparisons (one-way ANOVA)							
Dunnett t (2-sided)							
	**(I)**	**(J)**	**Mean Difference (I-J)**	**Std. Error**	**Sig.**	**95% Confidence Interval**	
						Lower Bound	Upper Bound
CD36	2	1	-0.15762	0.1141	0.481	-0.4874	0.1722
	3	1	0.20846	0.1141	0.265	-0.1214	0.5383
	4	1	-0.16171	0.1141	0.46	-0.4915	0.1681
	5	1	-0.13216	0.1141	0.618	-0.462	0.1976
c-myc	2	1	-0.30146	0.14593	0.186	-0.7233	0.1203
	3	1	-0.17378	0.14593	0.597	-0.5956	0.248
	4	1	-0.3613	0.14593	0.098	-0.7831	0.0605
	5	1	-0.27212	0.14593	0.251	-0.6939	0.1497
Sox-2	2	1	-0.40308	0.15497	0.08	-0.851	0.0449
	3	1	-.54276*	0.15497	0.018	-0.9907	-0.0948
	4	1	-.53779*	0.15497	0.019	-0.9857	-0.0898
	5	1	-0.40672	0.15497	0.077	-0.8547	0.0412
Vimentin	2	1	-0.23476	0.14078	0.332	-0.6417	0.1722
	3	1	-0.2385	0.14078	0.32	-0.6454	0.1684
	4	1	-0.3726	0.14078	0.074	-0.7795	0.0343
	5	1	-0.20325	0.14078	0.445	-0.6102	0.2037
p-Src (419)	2	1	-0.30366	0.16867	0.276	-0.7912	0.1839
	3	1	0.01927	0.16867	1	-0.4683	0.5068
	4	1	-0.2822	0.16867	0.329	-0.7697	0.2053
	5	1	-0.28818	0.16867	0.314	-0.7757	0.1994
p-Src (527)	2	1	-0.1045	0.32832	0.992	-1.0535	0.8445
	3	1	-0.32949	0.32832	0.716	-1.2785	0.6195
	4	1	-0.35796	0.32832	0.661	-1.307	0.591
	5	1	-0.32466	0.32832	0.726	-1.2737	0.6243
Src	2	1	-0.38802	0.13586	0.053	-0.7807	0.0047
	3	1	-.50584*	0.13586	0.013	-0.8985	-0.1132
	4	1	-0.3662	0.13586	0.069	-0.7589	0.0265
	5	1	-.65111*	0.13586	0.002	-1.0438	-0.2584
p-FAK	2	1	0.07401	0.33977	0.998	-0.9081	1.0561
	3	1	0.30562	0.33977	0.781	-0.6765	1.2877
	4	1	-0.15889	0.33977	0.969	-1.141	0.8232
	5	1	-0.38782	0.33977	0.628	-1.3699	0.5943
FAK	2	1	-0.11134	0.20195	0.947	-0.6951	0.4724
	3	1	-0.14193	0.20195	0.887	-0.7257	0.4418
	4	1	-0.31299	0.20195	0.389	-0.8967	0.2707

	5	1	-0.45818	0.20195	0.136	-1.0419	0.1256
MMP9	2	1	-0.19969	0.18771	0.678	-0.7423	0.3429
	3	1	0.08703	0.18771	0.97	-0.4555	0.6296
	4	1	-0.38001	0.18771	0.198	-0.9226	0.1626
	5	1	-0.21107	0.18771	0.639	-0.7536	0.3315
Ezrin	2	1	-0.26698	0.17823	0.416	-0.7821	0.2482
	3	1	0.25547	0.17823	0.451	-0.2597	0.7706
	4	1	-0.05844	0.17823	0.991	-0.5736	0.4567
	5	1	-0.09131	0.17823	0.958	-0.6065	0.4239
CD44	2	1	-0.15762	0.1141	0.481	-0.4874	0.1722
	3	1	0.20846	0.1141	0.265	-0.1214	0.5383
	4	1	-0.16171	0.1141	0.46	-0.4915	0.1681
	5	1	-0.13216	0.1141	0.618	-0.462	0.1976

*. The mean difference is significant at the 0.05 level.

Table S35. Statistical analysis performed for the comparison of the expression of FABP-EET network markers in MDA-MB-231, LM6 and FABP4/FABP5CYP2C19-depleted cells (Figure 4f; cells co-cultured with THP-1 monocytes). Mean protein expressions (as fold change relative to the parental 231 cells) for the three experiments were compared using one-way ANOVA, *post hoc* Dunnett's test, with protein expression of parental 231 cells as control; $p < 0.05$.

Multiple Comparisons (one-way ANOVA)							
Dunnett t (2-sided)							
**Dependent Variable**	**(I)**	**(J)**	**Mean Difference (I-J)**	**Std. Error**	**Sig.**	**95% Confidence Interval**	
						Lower Bound	Upper Bound
CD36	2	1	-0.44845	0.35886	0.56	-1.4857	0.5888
	3	1	0.17973	0.35886	0.961	-0.8576	1.217
	4	1	-0.40744	0.35886	0.632	-1.4447	0.6298
	5	1	-0.31115	0.35886	0.8	-1.3484	0.7261
c-myc	2	1	-0.1313	0.12809	0.703	-0.5015	0.2389
	3	1	-0.33146	0.12809	0.082	-0.7017	0.0388
	4	1	.39147*	0.12809	0.038	0.0212	0.7617
	5	1	-0.23373	0.12809	0.266	-0.604	0.1365
Sox-2	2	1	-0.031	0.32297	1	-0.9645	0.9025
	3	1	0.07467	0.32297	0.998	-0.8589	1.0082
	4	1	0.64413	0.32297	0.207	-0.2894	1.5777
	5	1	-0.11996	0.32297	0.986	-1.0535	0.8136
Vimentin	2	1	0.31169	0.27796	0.641	-0.4917	1.1151
	3	1	0.75971	0.27796	0.065	-0.0437	1.5631
	4	1	0.13409	0.27796	0.966	-0.6693	0.9375
	5	1	0.60657	0.27796	0.156	-0.1969	1.41
p-Src (419)	2	1	-0.08656	0.15685	0.947	-0.5399	0.3668
	3	1	-0.22353	0.15685	0.456	-0.6769	0.2298
	4	1	0.40947	0.15685	0.079	-0.0439	0.8628
	5	1	-0.07248	0.15685	0.971	-0.5258	0.3809
p-Src (527)	2	1	-0.11607	0.29698	0.984	-0.9745	0.7423
	3	1	0.17285	0.29698	0.937	-0.6856	1.0313
	4	1	-0.1798	0.29698	0.928	-1.0382	0.6786
	5	1	0.24851	0.29698	0.817	-0.6099	1.1069
Src	2	1	0.01819	0.28257	1	-0.7986	0.835
	3	1	-0.3129	0.28257	0.65	-1.1297	0.5039
	4	1	0.01237	0.28257	1	-0.8044	0.8291
	5	1	-0.19134	0.28257	0.899	-1.0081	0.6254
p-FAK	2	1	0.02957	0.10523	0.995	-0.2746	0.3337
	3	1	0.13685	0.10523	0.529	-0.1673	0.441
	4	1	0.14588	0.10523	0.478	-0.1583	0.4501
	5	1	0.00454	0.10523	1	-0.2996	0.3087
FAK	2	1	-0.30374	0.20887	0.439	-0.9075	0.3
	3	1	-0.11955	0.20887	0.94	-0.7233	0.4842
	4	1	-0.25212	0.20887	0.587	-0.8558	0.3516
	5	1	-0.41387	0.20887	0.211	-1.0176	0.1898
MMP9	2	1	-0.2643	0.162	0.349	-0.7326	0.2039

	3	1	-0.22585	0.162	0.473	-0.6941	0.2424
	4	1	-0.39066	0.162	0.109	-0.8589	0.0776
	5	1	-0.157	0.162	0.738	-0.6252	0.3113
Ezrin	2	1	-0.2845	0.2189	0.529	-0.9172	0.3482
	3	1	0.2575	0.2189	0.606	-0.3752	0.8902
	4	1	-0.17878	0.2189	0.829	-0.8115	0.4539
	5	1	-0.1024	0.2189	0.969	-0.7351	0.5303
CD44	2	1	-0.44845	0.35886	0.56	-1.4857	0.5888
	3	1	0.17973	0.35886	0.961	-0.8576	1.217
	4	1	-0.40744	0.35886	0.632	-1.4447	0.6298
	5	1	-0.31115	0.35886	0.8	-1.3484	0.7261

*. The mean difference is significant at the 0.05 level.

Table S36. Statistical analysis performed for the effects of dLGG (40 μM) on the expression level of a FABP-EET network proteins in LM6 cells. (Figure 7a). Mean protein expressions (as fold change relative to vehicle-treated cells) for the three experiments were compared using one-way ANOVA, *post hoc* Dunnett's test, with protein expression of vehicle-treated cells as control; $p < 0.05$.

t-test for independent samples									
**Levene's Test for Equality of Variances**			**t-test for Equality of Means**						
		**Sig.**	**t**	**Df**	**Sig. (2-tailed)**	**Mean Difference**	**Std. Error Difference**	95% Confidence Interval of the Difference	
								Lower	Upper
**FABP4**	Equal variances assumed						0.1913	0.04538	1.10766
	Equal variances not assumed		3.014	4	0.039	0.57652	0.013333	-0.04404	0.070702
**FABP5**	Equal variances assumed	0.02	0.095	0.57652	0.016648	-0.66667	0.168259	-1.13383	-0.1995
	Equal variances not assumed		3.442	4	0.026	0.33887	0.168259	-1.39063	0.057294
**CYP2C19**	Equal variances assumed	0.02	0.075	0.33887	2.07E-07	-1.27	0.017321	-1.31809	-1.22191
	Equal variances not assumed		4	0.194	0.17945	-1.27	0.017321	-1.34452	-1.19548
**PPAR-γ**	Equal variances assumed	0.02	0.26	0.17945	0.000567	-2.11333	0.211844	-2.70151	-1.52516
	Equal variances not assumed		4.05	4	0.015	0.27357	0.211844	-3.02482	-1.20184
**SREBP-2**	Equal variances assumed	0.06	0.056	0.27357	0.002175	-0.39333	0.056075	-0.54902	-0.23764
	Equal variances not assumed		4.965	4	0.008	0.56976	0.056075	-0.63461	-0.15206

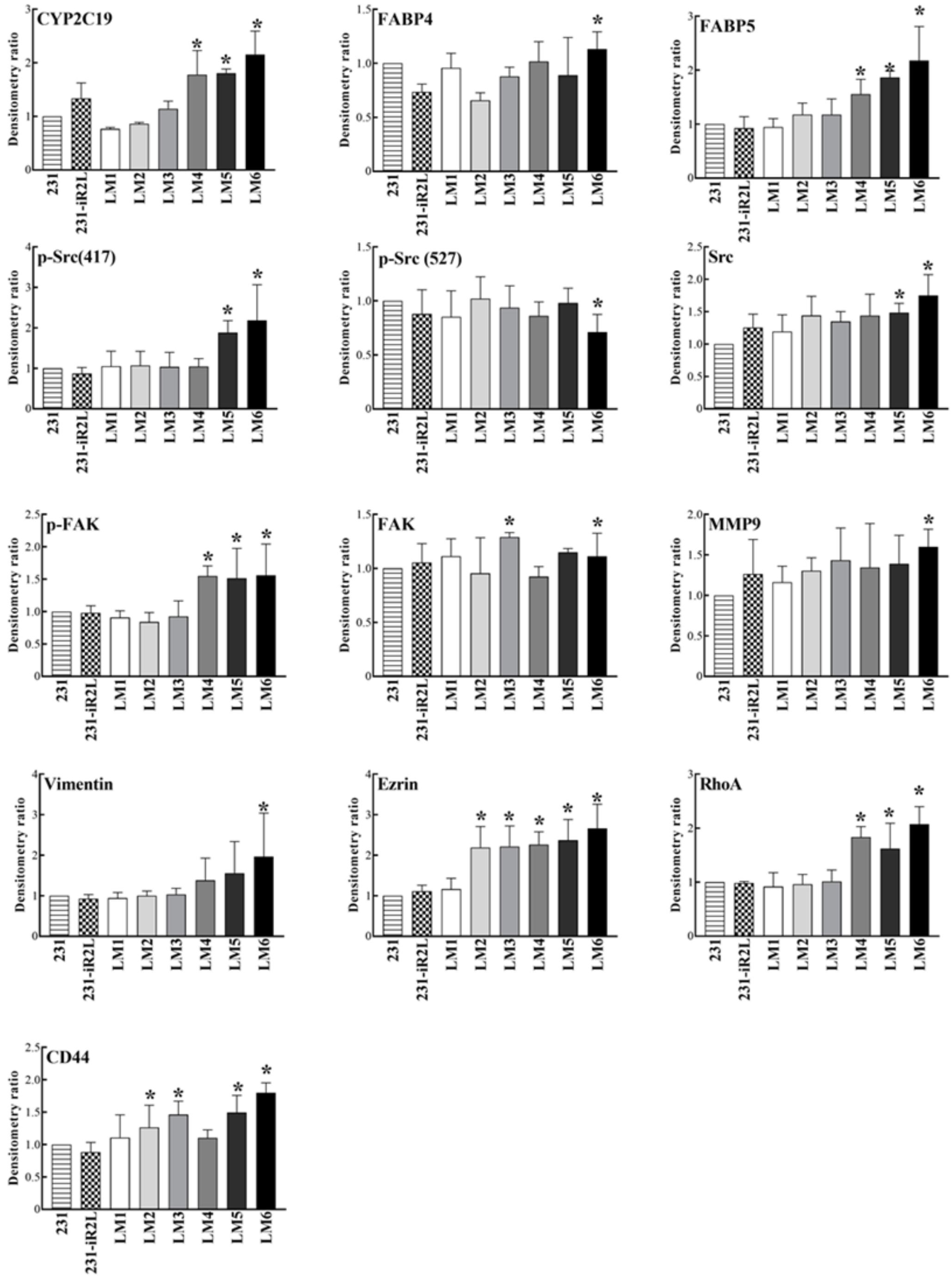

Figure S1. Relative protein expression levels calculated from three independent western blotting experiments for representative data shown in Figure 2b. Densitometry analysis was performed using ImageJ. Data are presented as mean ± SD protein expression relative to parental 231 cells. Statistical analysis was performed using one-way ANOVA, *post hoc* Dunnett's test, with protein expression of parental 231cells as control. Asterisks indicate protein expressions significantly higher compared with parental 231 control, $p < 0.05$.

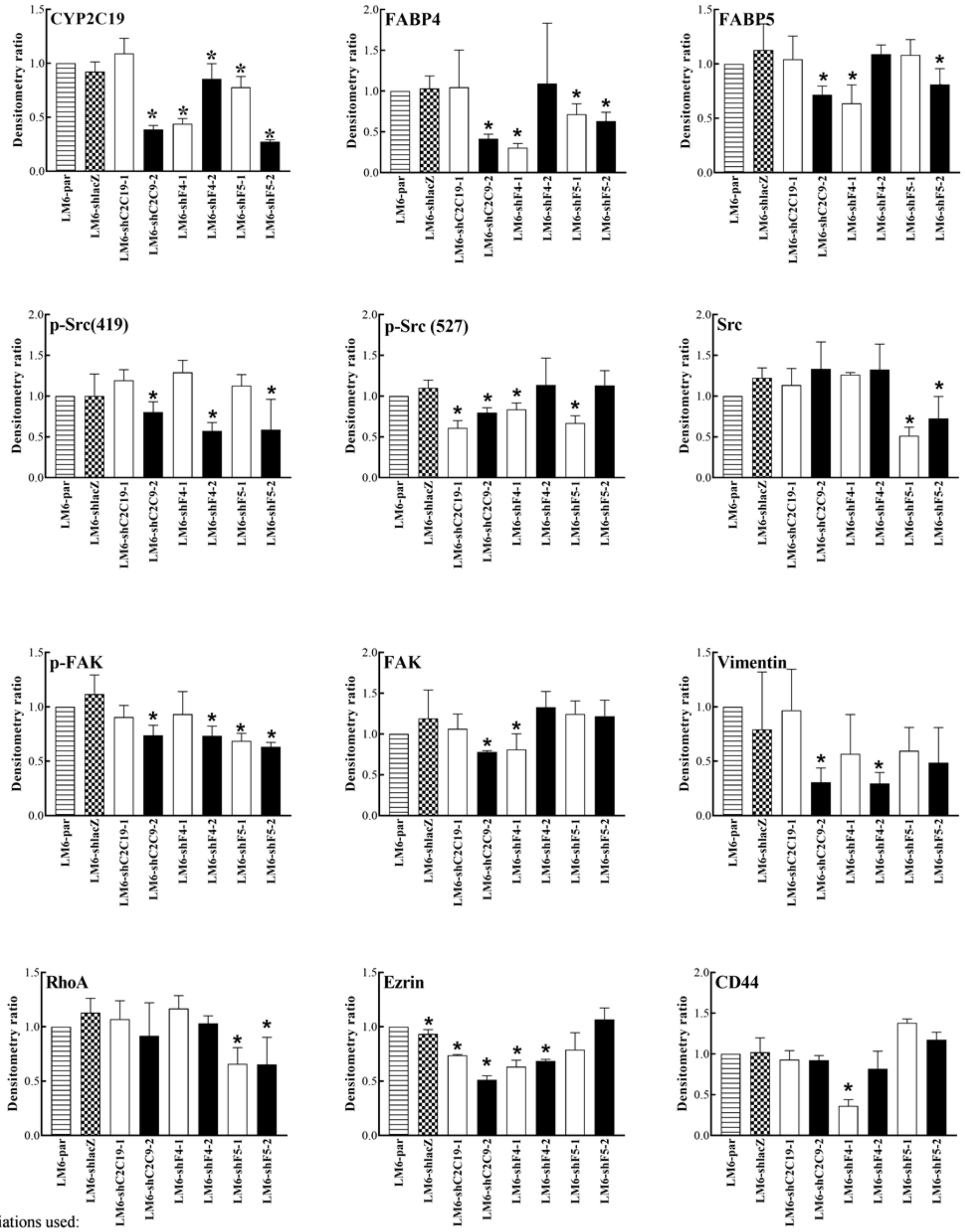

Abbreviations used:
LM6-shC2C19: LM6-shCYP2C19
LM6-shF4: LM6-shFABP4
LM6-shF5: LM6-shFABP5

Figure S2. Relative protein expression levels calculated from three independent western blotting experiments for representative data shown in Figure 2c. Densitometry analysis was performed using ImageJ. Data are presented as mean ± SD relative to LM6 cells. Statistical analysis was performed using one-way ANOVA, *post hoc* Dunnett's test, with protein expression of parental LM6 cells as control. Asterisks indicate protein expressions significantly lower compared with the parental LM6 control, $p < 0.05$.

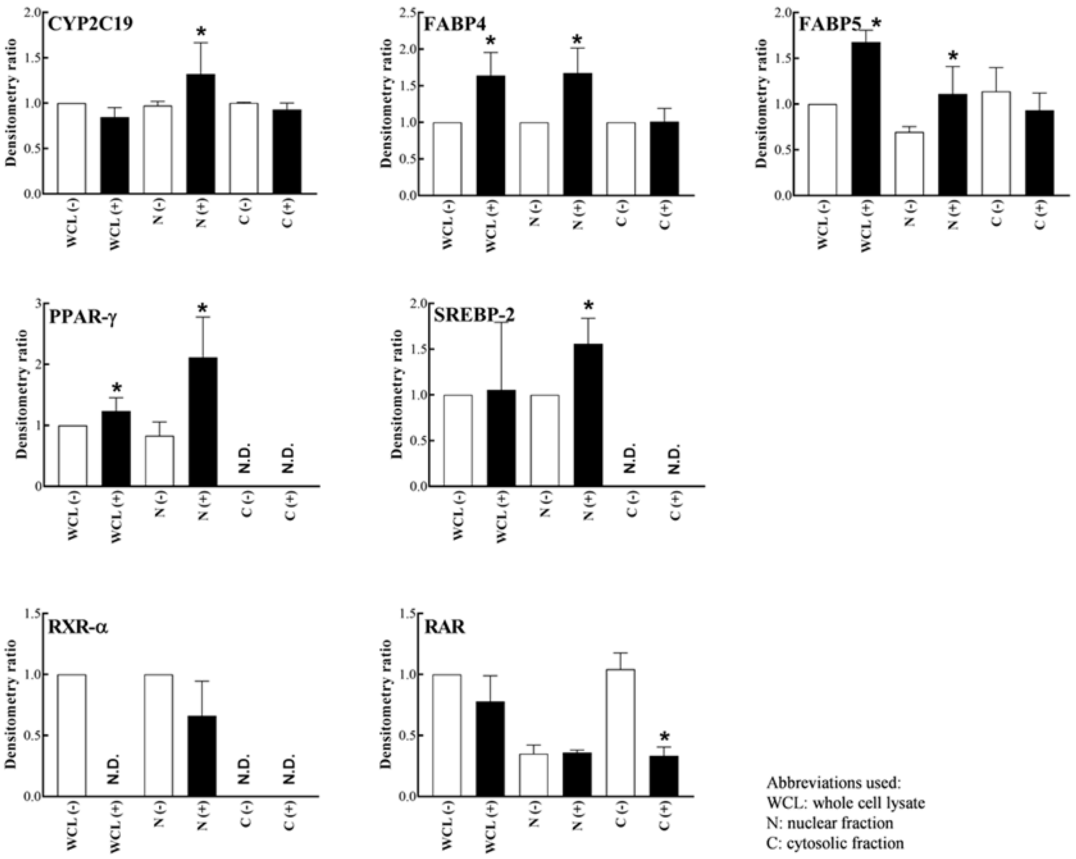

Figure S3. Relative protein expression levels calculated from three independent western blotting experiments for representative data shown in Figure 3e. Densitometry analysis was performed using ImageJ. Data are presented as mean ± SD relative to vehicle-treated cells. Statistical analysis was performed using student's t-test, with protein expression in corresponding vehicle-treated cells as control. Asterisks indicate statistical significance, $p < 0.05$. (N.D.: protein expression not detected by western blotting).

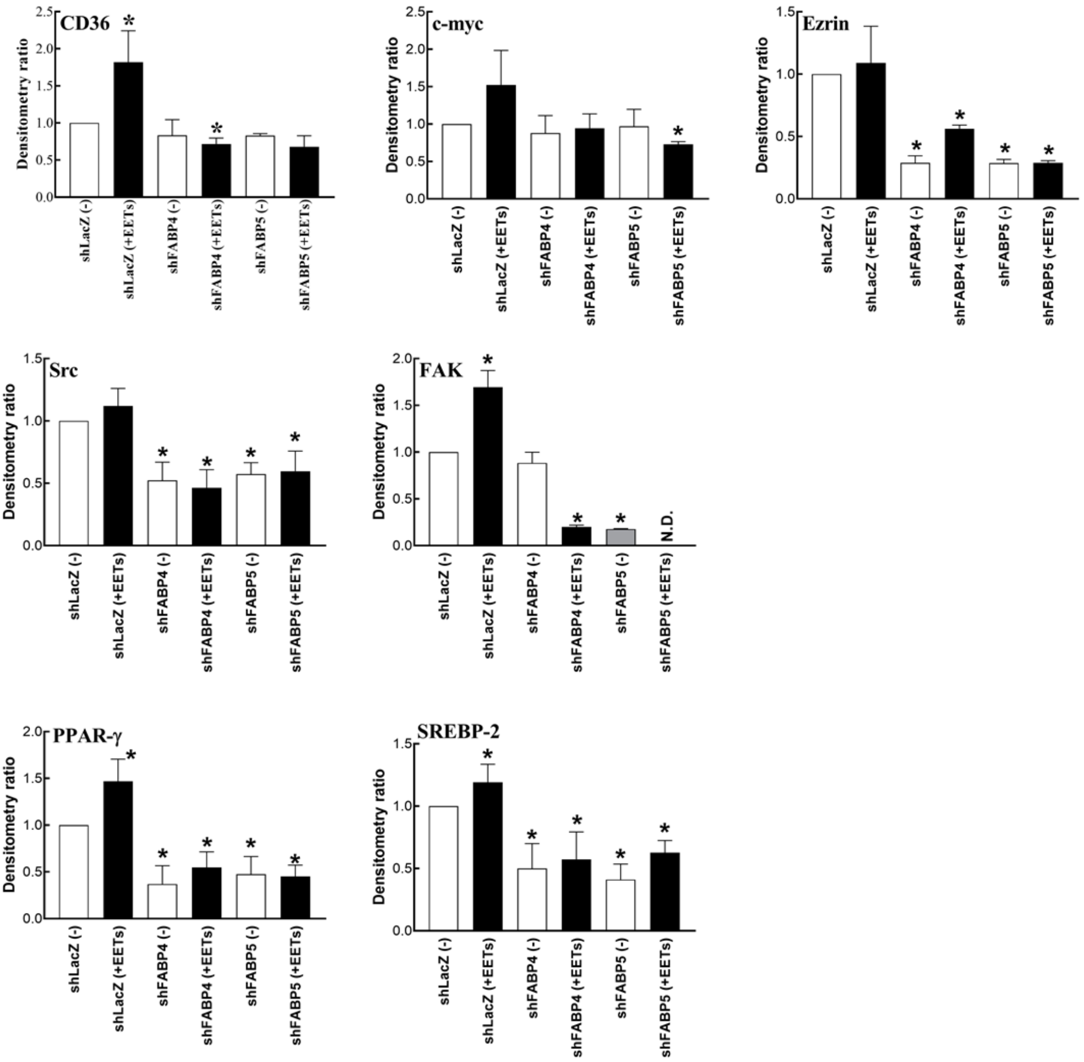

Figure S4. Relative protein expression levels calculated from three independent western blotting experiments for representative data shown in Figure 3f. Densitometry analysis was performed using ImageJ. Data are presented as mean ± SD protein expression relative to the vehicle-treated cells. Statistical analysis was performed using student's t-test, with protein expression of corresponding vehicle-treated samples as control. Asterisks indicate statistical significance, $p < 0.05$. (N.D.: protein expression not detected by western blotting).

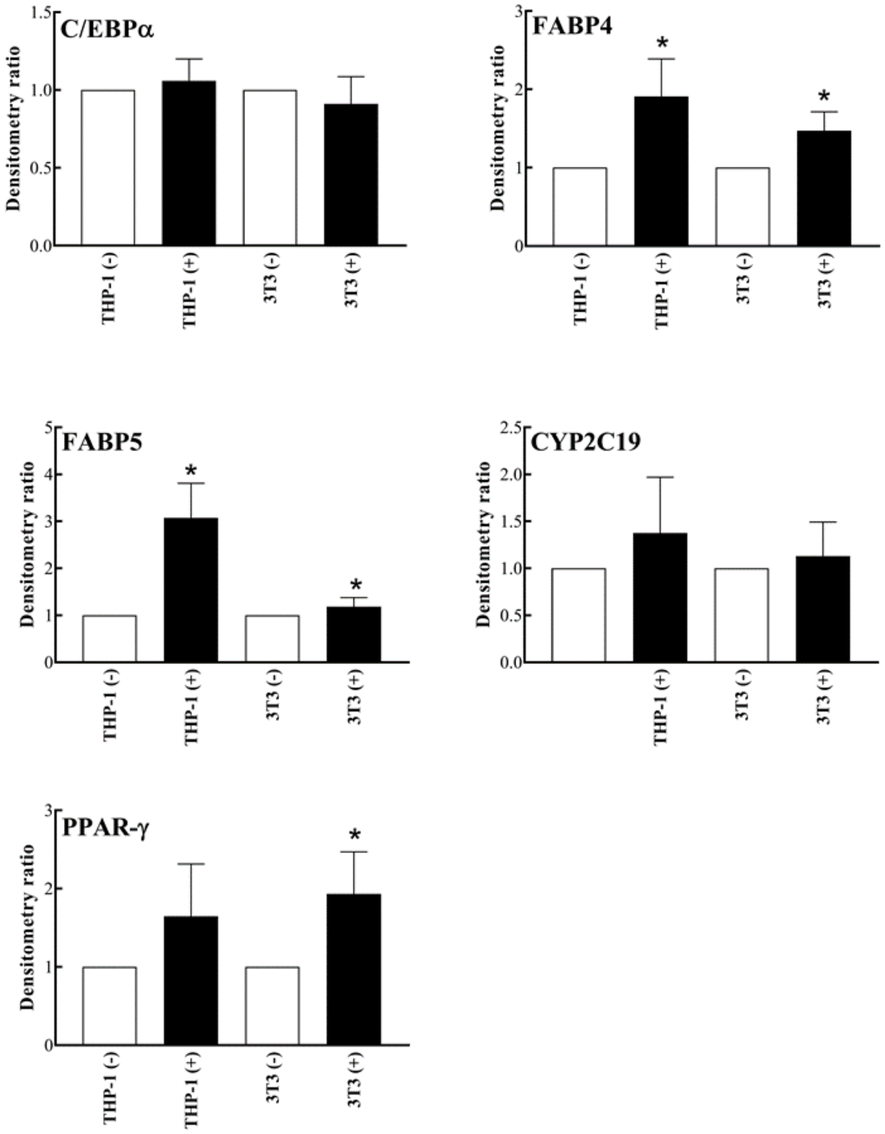

Figure S5. Relative protein expression levels calculated from three independent western blotting experiments for representative data shown in Figure 4a. Densitometry analysis was performed using ImageJ. Data are presented as mean ± SD protein expression relative to vehicle-treated cells. Statistical analysis was performed using student's t-test, with protein expression of corresponding vehicle-treated cells as control. Asterisks indicate statistical significance, $p < 0.05$.

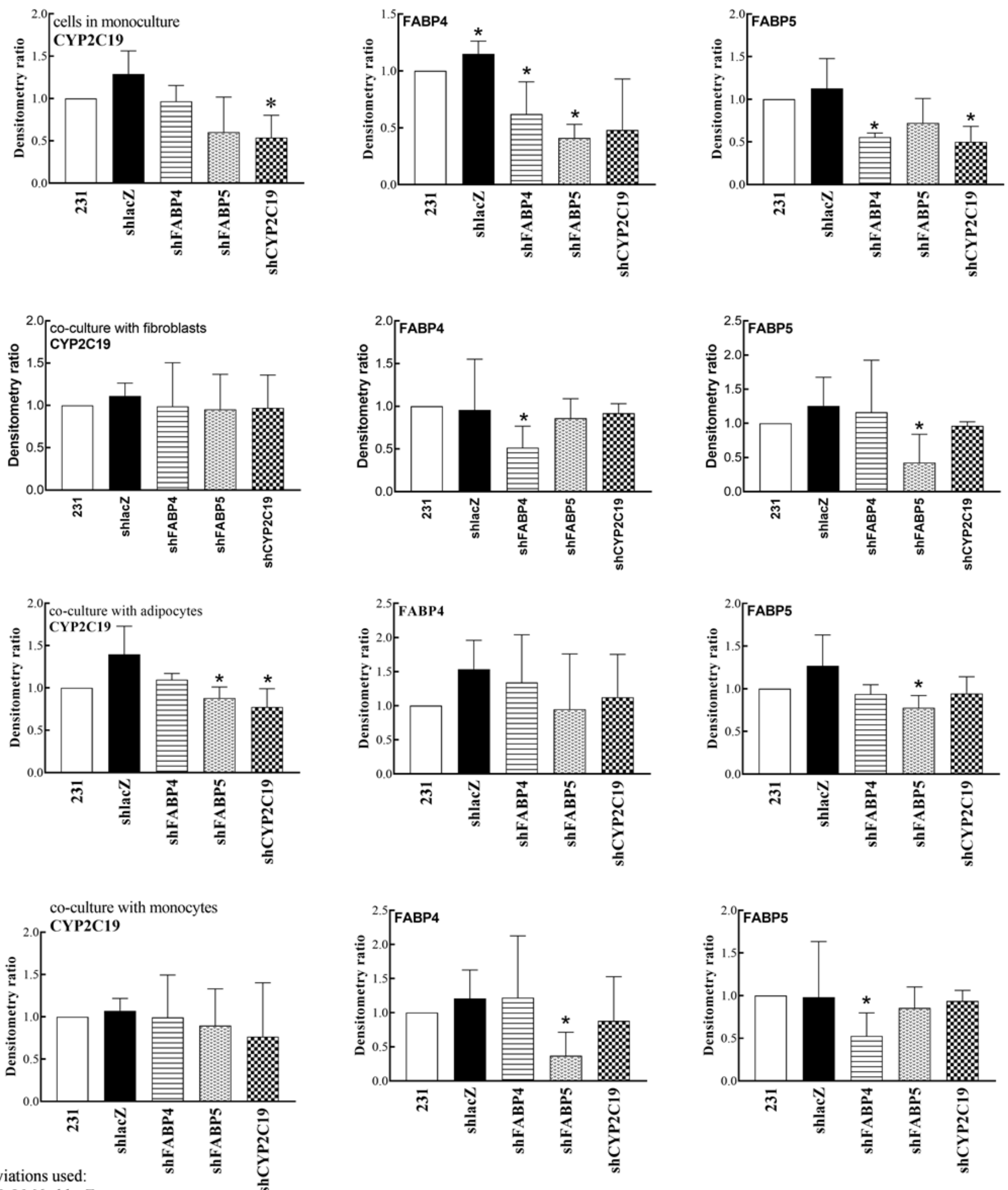

Abbreviations used:
shLacZ: LM6-shlacZ
shCYP2C19: LM6-shCYP2C19
shFABP4: LM6-shFABP4
shFABP5: LM6-shFABP5

Figure S6. Relative protein expression levels calculated from three independent western blotting experiments for representative data shown in Figure 4b-cells grown in monoculture or co-cultured with stromal cells (fibroblasts, adipocytes, monocytes). Densitometry analysis was performed using ImageJ, data are presented as mean ± SD protein expression relative to parental 231 cells. Statistical analysis was performed using one-way ANOVA, *post hoc* Dunnett's test, with protein expression of 231 cells as control. Asterisks indicate statistical significance, $p < 0.05$.

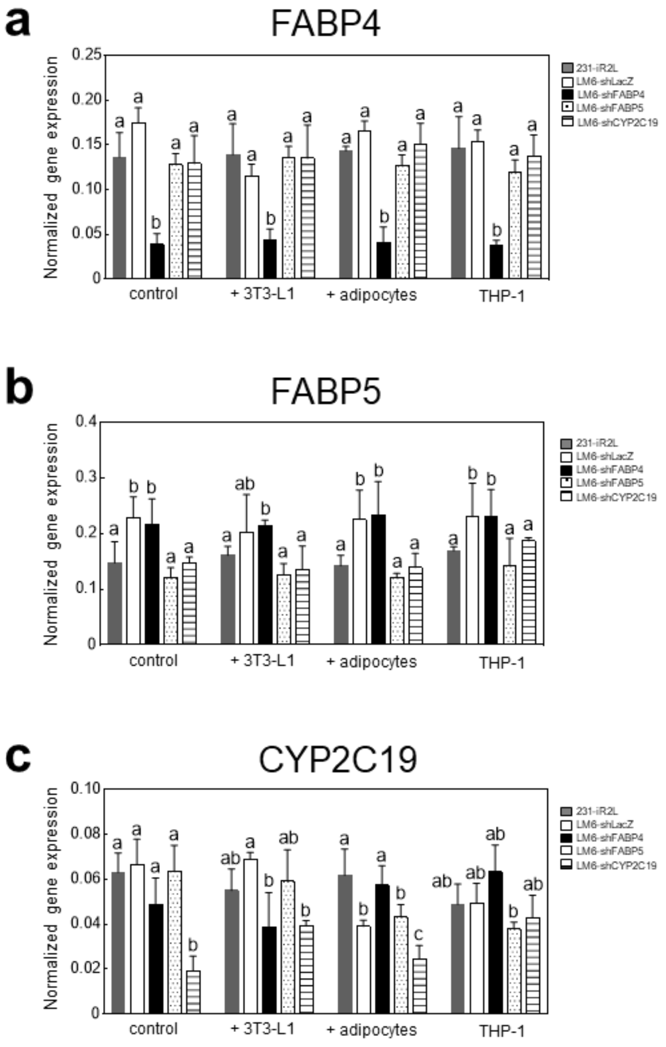

Figure S7. Gene expression levels (**a** FABP4, **b** FABP5 and **c** CYP2C19) in the TNBC cell lines co-cultured with fibroblasts, adipocytes and monocyte measured against GADPH as control.

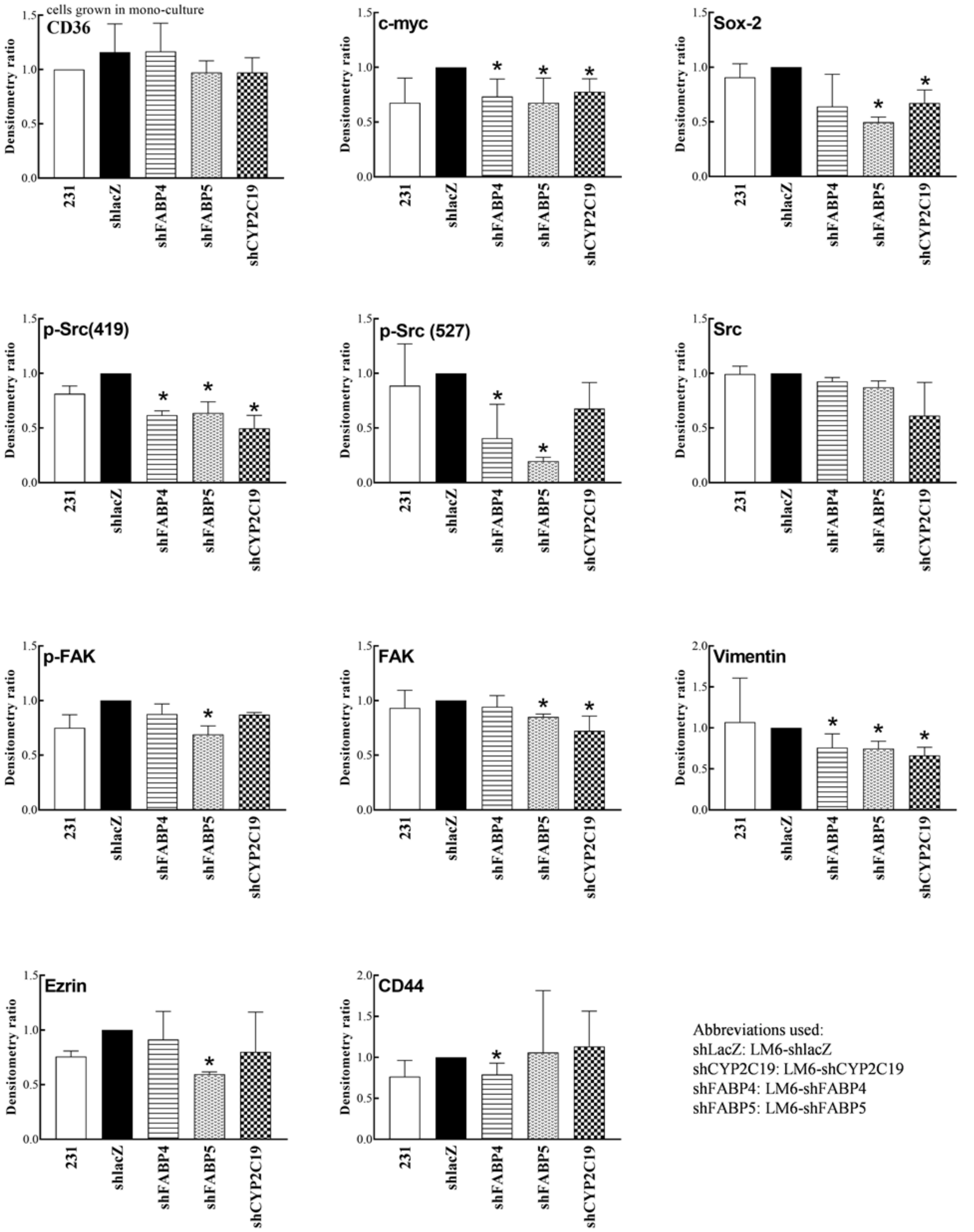

Figure 8. Relative protein expression levels calculated from three independent western blotting experiments for representative data shown in Figure 5f-cells grown in monoculture. Densitometry quantification was performed using ImageJ, data are presented as mean ± SD protein expression relative to LM6-shLacZ cells. Statistical analysis was performed using one-way ANOVA, *post hoc* Dunnett's test, with protein expression of LM6-shLacZ cells as control. Asterisks indicate statistical significance, $p < 0.05$.

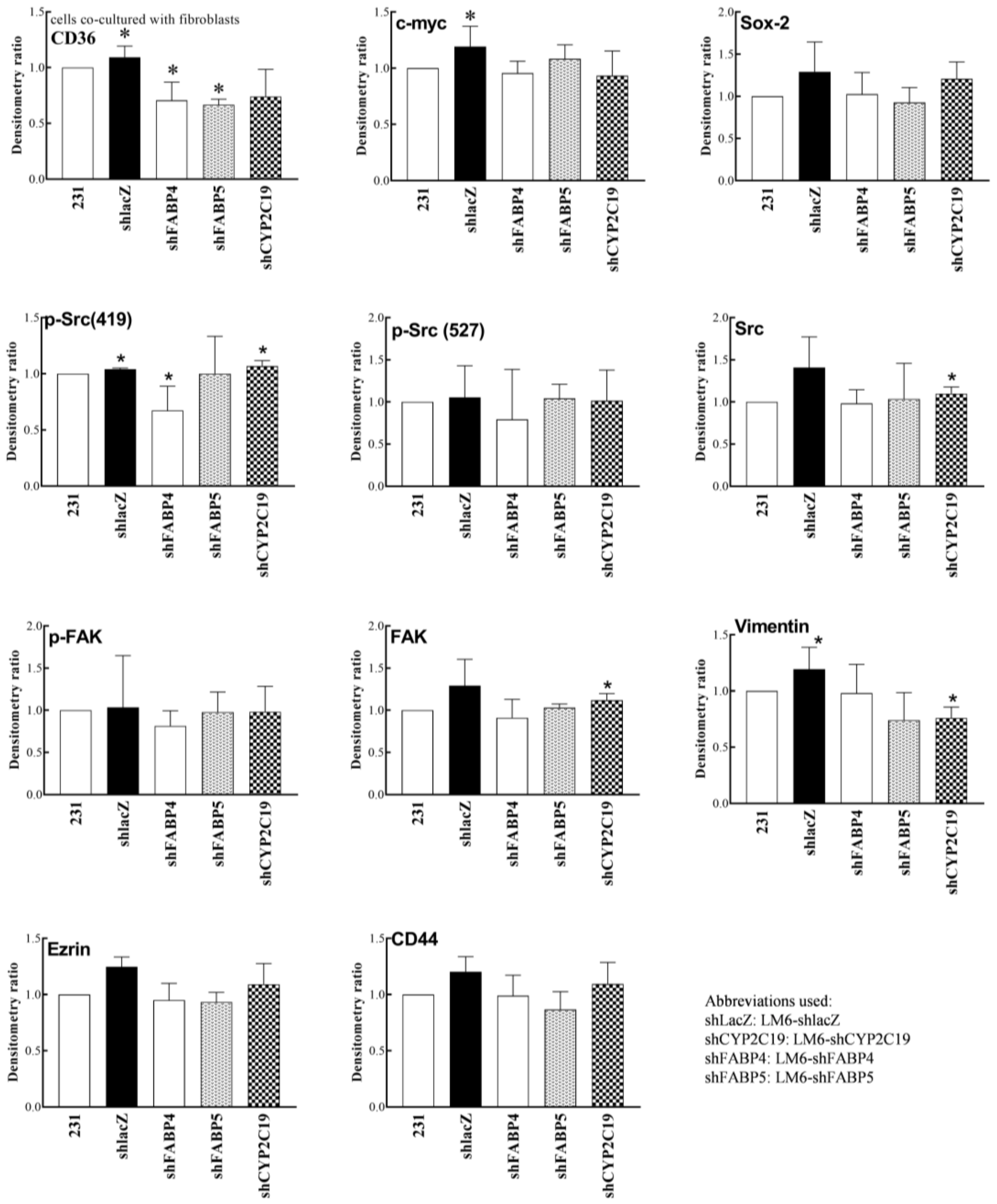

Figure S9. Relative protein expression levels calculated from three independent western blotting experiments for representative data shown in Figure 4f-cells co-cultured with fibroblasts. Densitometry quantification was performed using ImageJ. Data are presented as mean ± SD protein expression relative to parental 231 cells. Statistical analysis was performed using one-way ANOVA, *post hoc* Dunnett's test, with protein expression of parental 231 cells as control. Asterisks indicate statistical significance, $p < 0.05$.

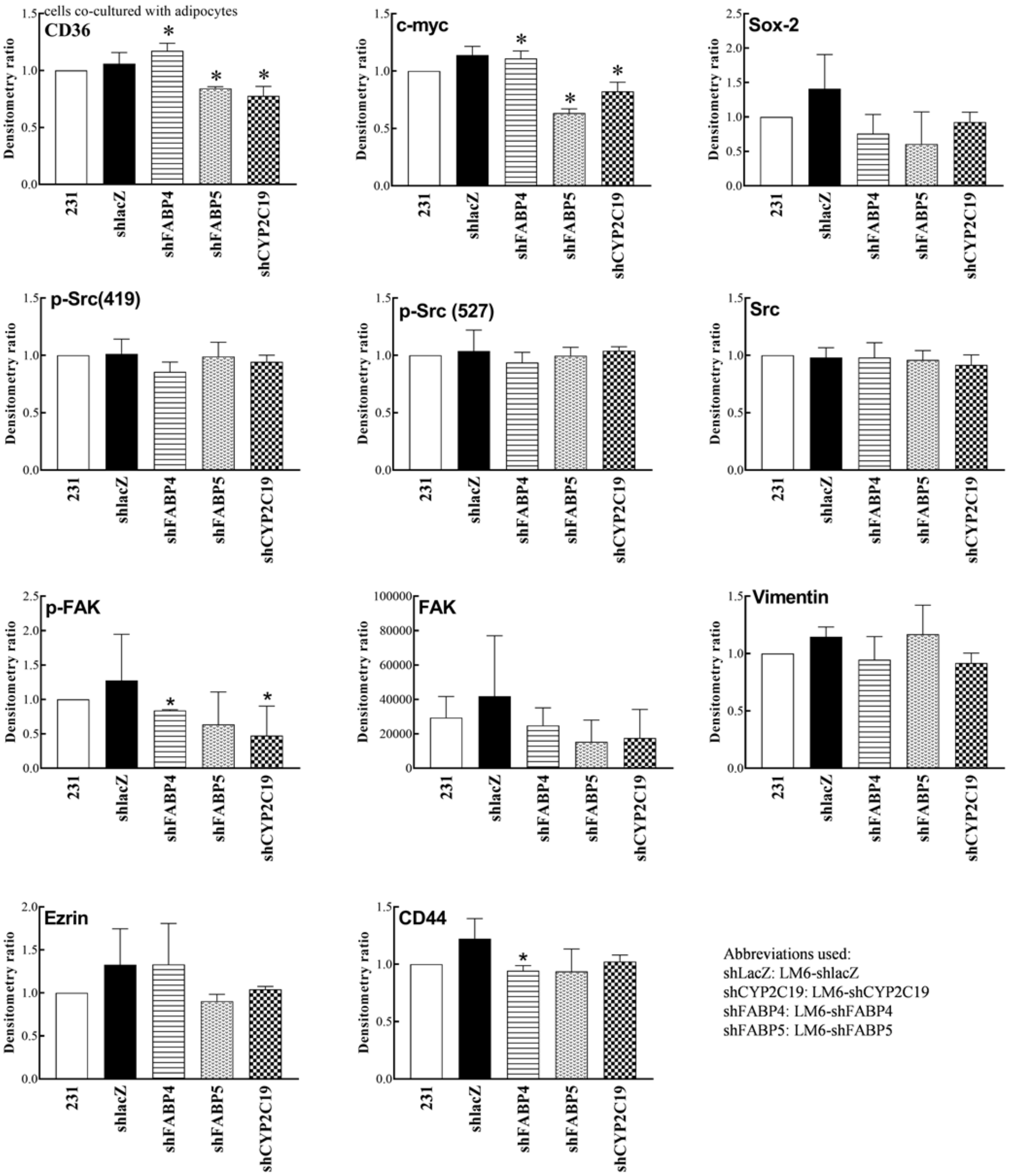

Figure S10. Relative protein expression levels calculated from three independent western blotting experiments for representative data shown in Figure 4f- cells co-cultured with adipocytes. Densitometry analysis was performed using ImageJ, data are presented as mean ± SD protein expression relative to parental 231 cells. Statistical analysis was performed using one-way ANOVA, *post hoc* Dunnett's test, with protein expression of parental 231 cells as control. Asterisks indicate statistical significance, $p < 0.05$.

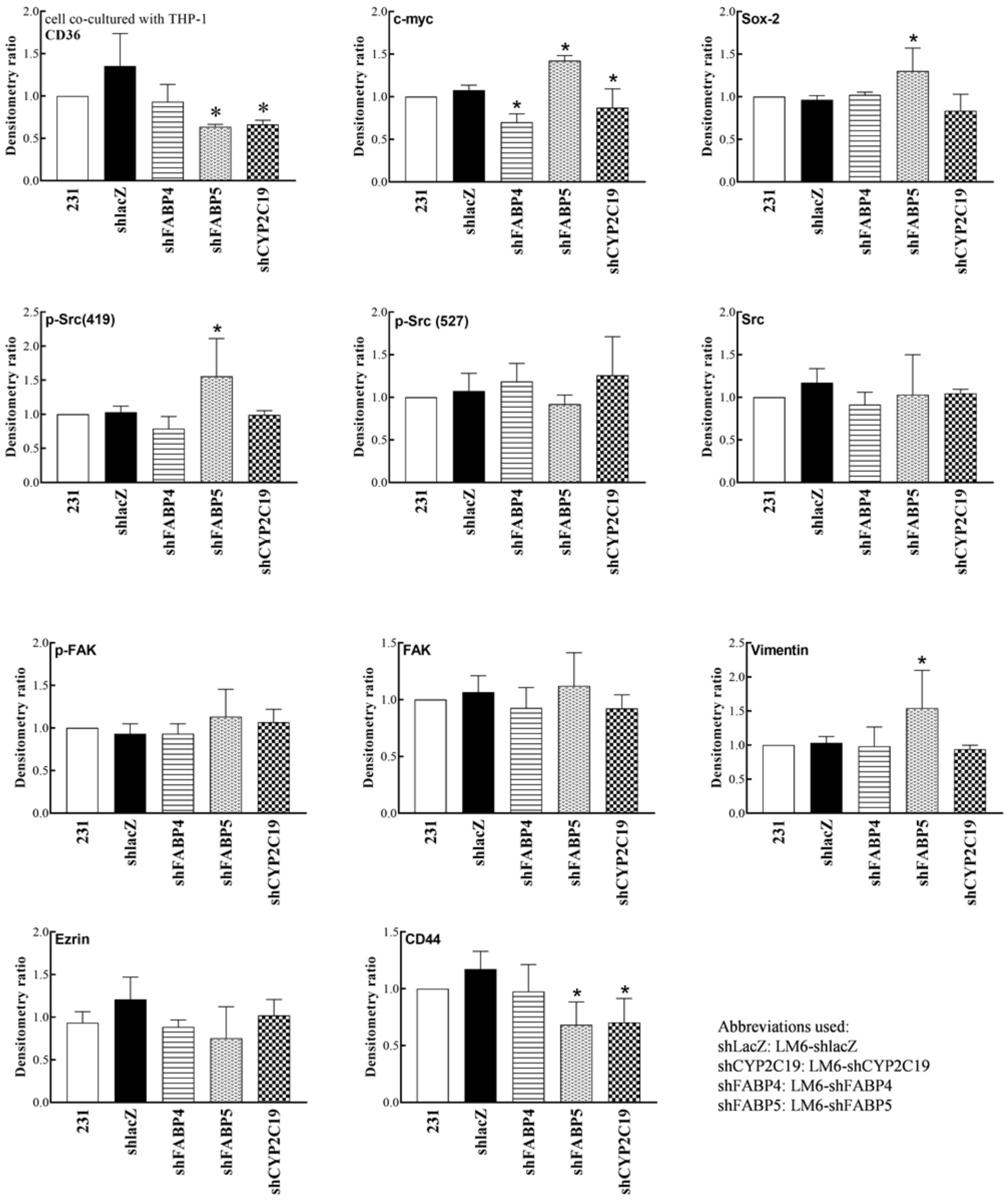

Figure S11. Relative protein expression levels calculated from three independent western blotting experiments for representative data shown in Figure 4f-cells co-cultured with monocytes. Densitometry analysis was performed using ImageJ. Data are presented as mean ± SD protein expression relative to 231 cells. Statistical analysis was performed using one-way ANOVA, *post hoc* Dunnett's test, with protein expression of parental 231 cells as control. Asterisks indicate statistical significance, $p < 0.05$.

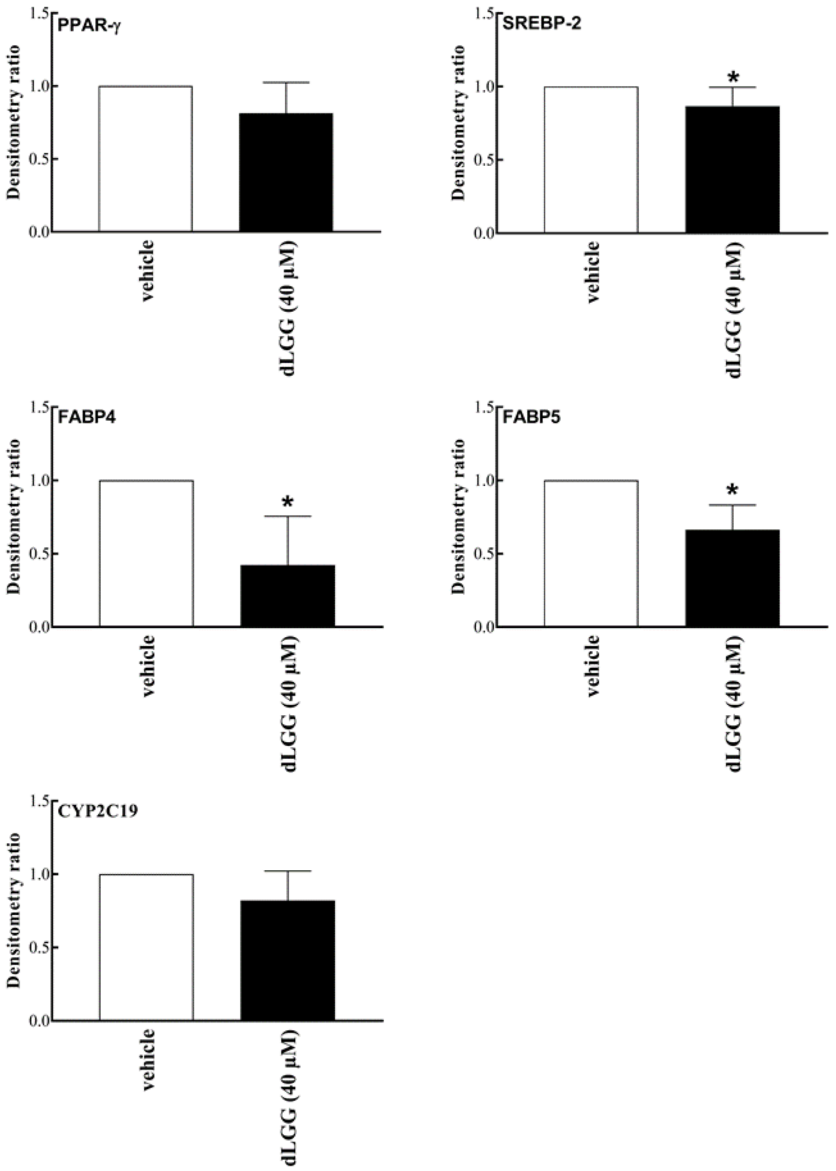

Figure S12. Relative protein expression levels calculated from three independent western blotting experiments for representative data shown in Figure 7a. Densitometry quantification was performed using ImageJ. Data are presented as mean ± SD protein expression relative to vehicle-treated cells. Statistical analysis was performed using student's t-test, with protein expression of vehicle-treated cells as control. Asterisks indicate statistical significance, $p < 0.05$.

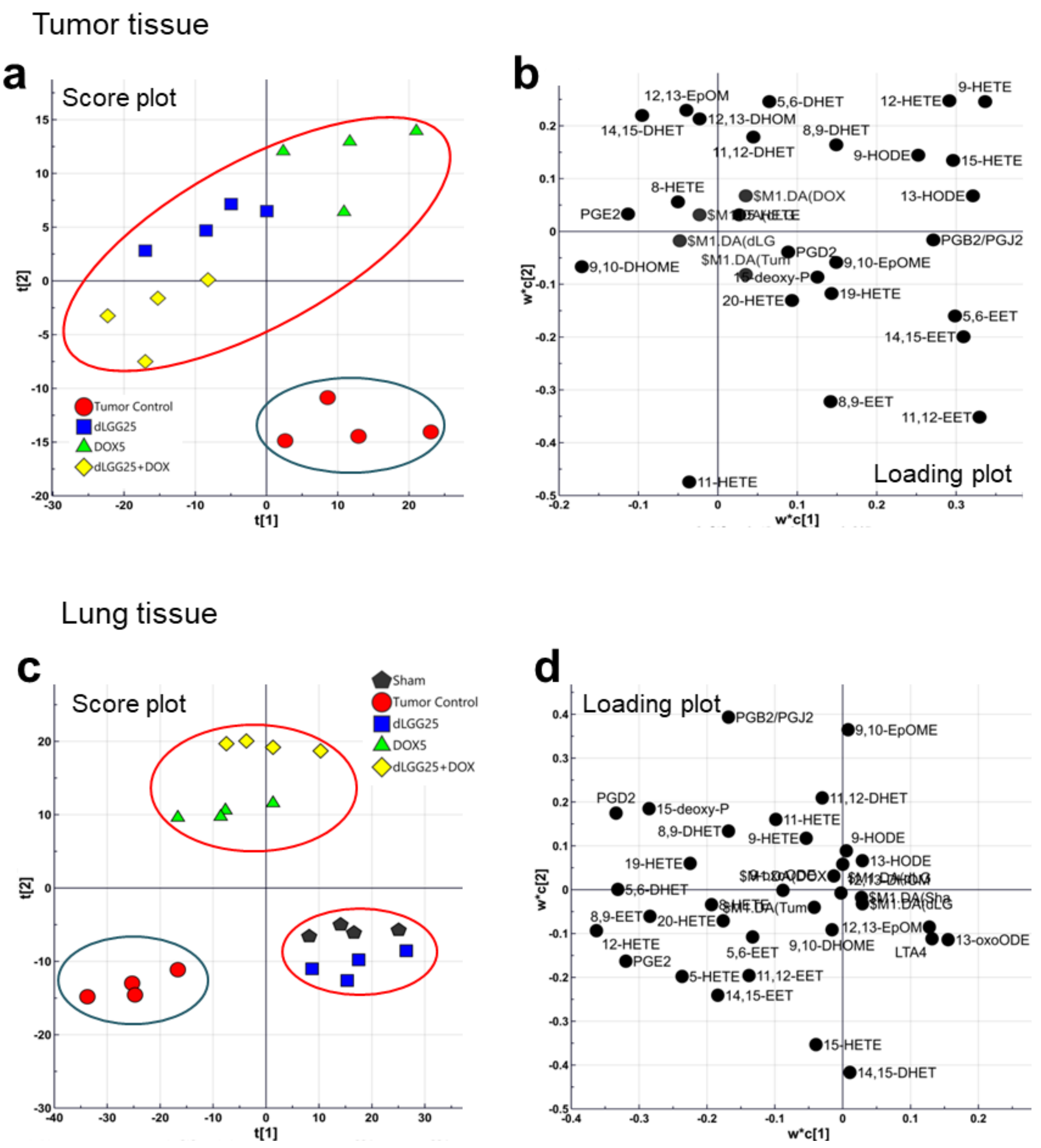

Figure S13. Oxylipin profile and cross-validated PLS-DA score and loading plots of (**a** and **b**) tumor and (**c** and **d**) lung tissues derived from LM6-bearing mice treated with dLGG25, DOX5, dLGG25+DOX5 in comparison with the tumor control (LM6) and sham groups. Each biological replicate (N = 4) is represented by a single point. All analyses include 4 technical replicates per biological sample.

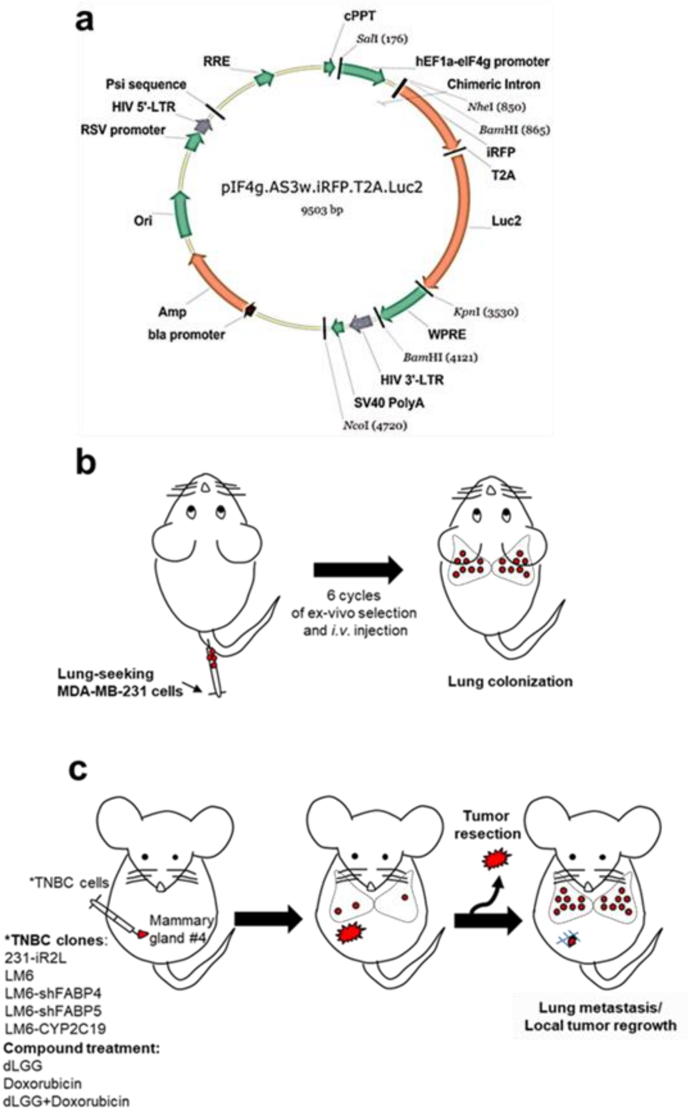

Figure S14. a MDA-MB-231 cells were stably transfected with red florescent protein (iRFP) and firefly luciferase (luc2) of pGL4 (Promega) driven by a hybrid EF1α/eIF4g promoter (InvivoGen) through lentiviral infection. All cell lines were routinely cultured in DMEM, 10% antibiotics and 10% FBS. Parental iRFP and Luc2 expressing MDA-MD-231 cells (231-iR2L) and the lung seeking (LM6) derivative were authenticated by comparing with short tandem repeat DNA profiles in the ATCC database within 6 months of the last experiment. **b** Schematic diagram showing how the highly malignant lung seeking TNBC cells (LM6) were derived. 2×10^5 suspension of iRFP/luciferase-expressing MDA-MB-231 cells was injected into SCID mice through the tail vein. Mice developed lung nodules after 6 weeks and iRFP/Luc expressing cells were isolate by flow cytometry. This procedure was repeated six times to obtain LM6. **c** Schematic diagram showing the orthotopic injection of TNBC cells (231-iR2L, LM6 and LM6 derivatives) in NOD/SCID mice, followed by tumor resection at approximately 500 mm^3. Effect of protein depletion or compound treatment on primary tumor growth, relapse and lung metastasis monitoring was measured by IVIS.

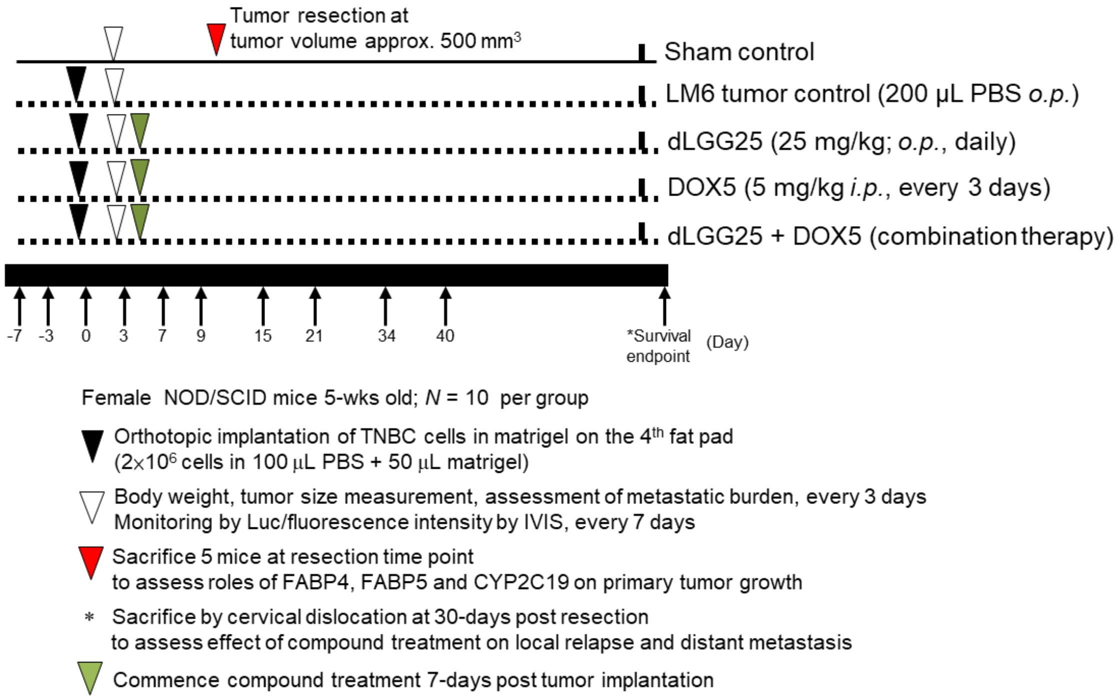

Figure S15. Primary tumor growth, relapse after tumor resection and metastasis rates were compared in animal groups inoculated with $2{\times}10^{6}$ 231-iR2L, lung-seeking MDA-MB-231 (LM6), and LM6 with depleted FABP4 (LM6-shFABP4), FABP5 (LM6-FABP5) or CYPC19 (LM6-shCYP2C19).

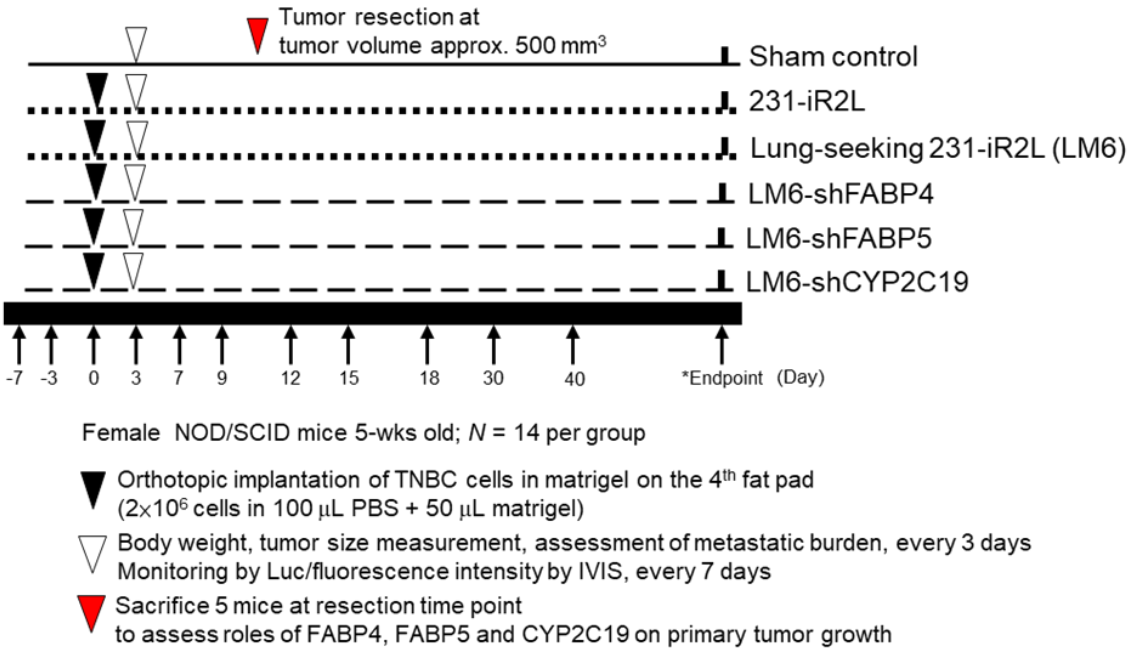

Figure S16. Effects of dLGG (dLGG25; 25 mg/kg *o.p.*, daily), doxorubicin (DOX5; 5 mg/kg *i.p.*, every 3 days) and combination treatment of dLGG and doxorubicin (dLGG25 + DOX5) on primary tumor growth, local relapse and lung metastasis rates of LM6 TNBC tumors were compared. Survival analysis was performed up to 100 days post-tumor inoculation.

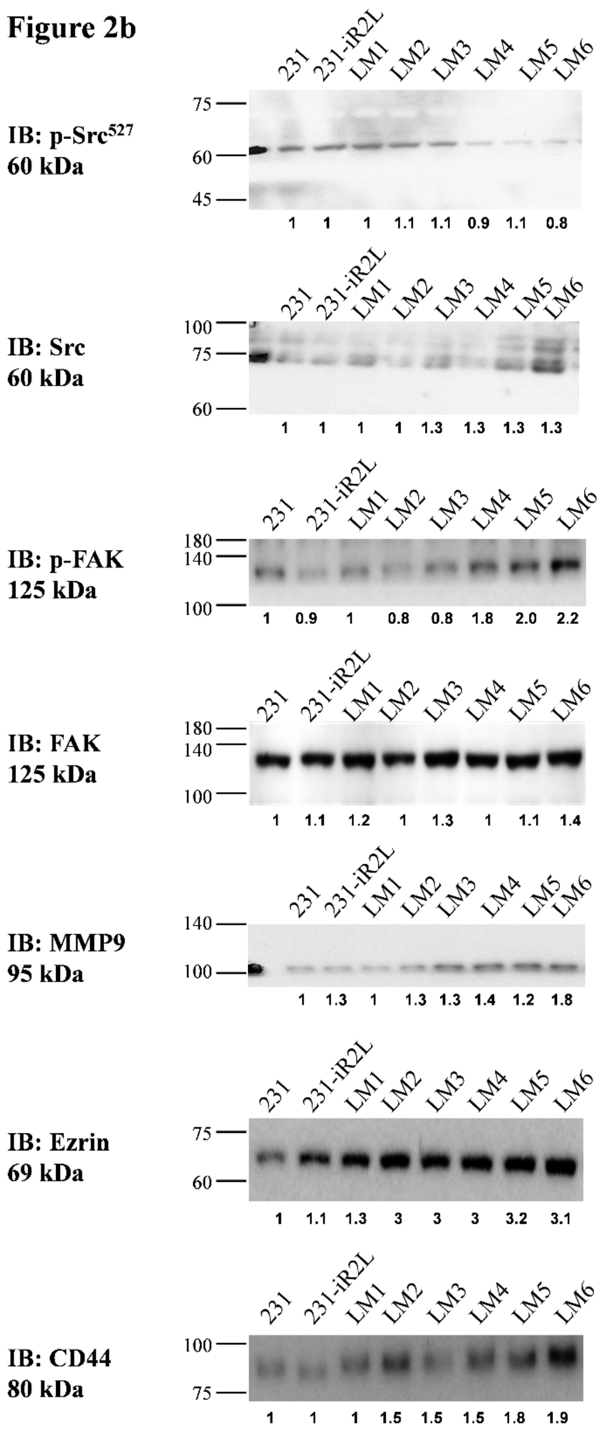

Figure S17. Representative Western blot image corresponding to Figure 2b showing all protein bands (p-Src^{527}, Src, p-FAK, FAK, MMP9, ezrin and CD44) and molecular weight markers. Densitometry quantification of three independent experiments was performed using ImageJ.

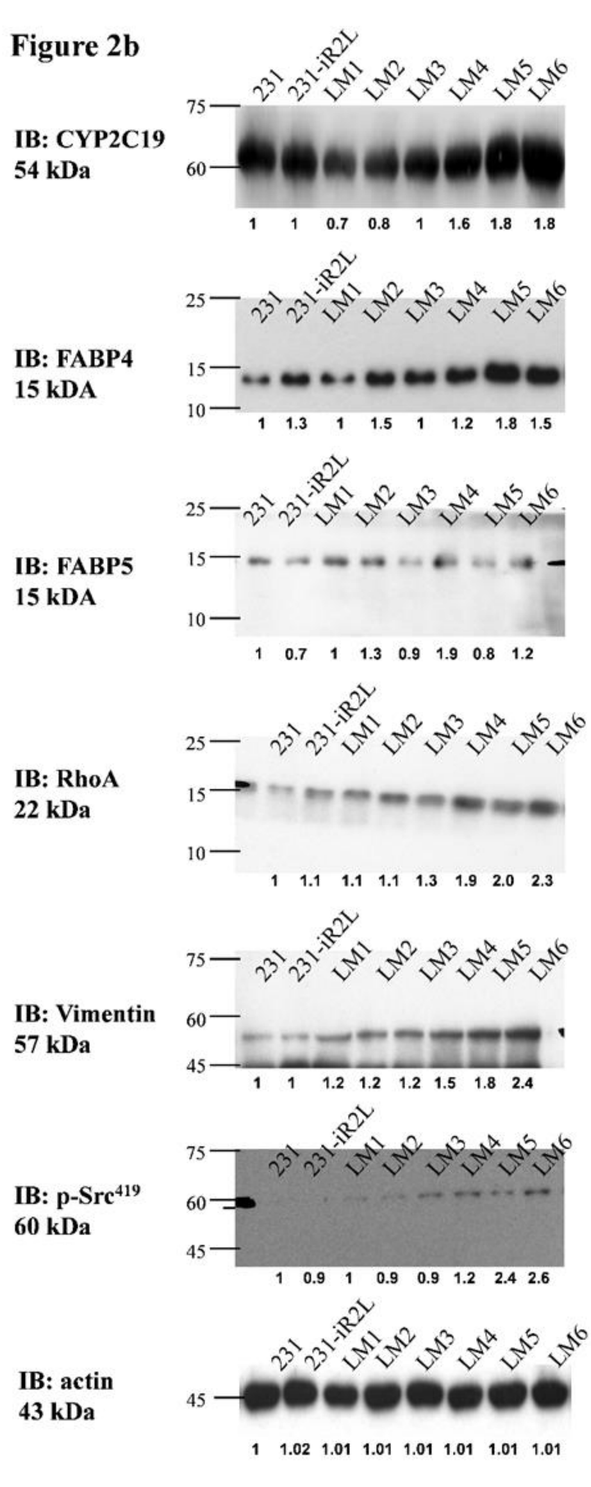

Figure S18. Representative Western blot image corresponding to Figure 2b showing all protein bands (CYP2C19, FABP4, FABP5, RhoA, vimentin, p-Src^{419} and actin) and molecular weight markers. Densitometry quantification of three independent experiments was performed using ImageJ.

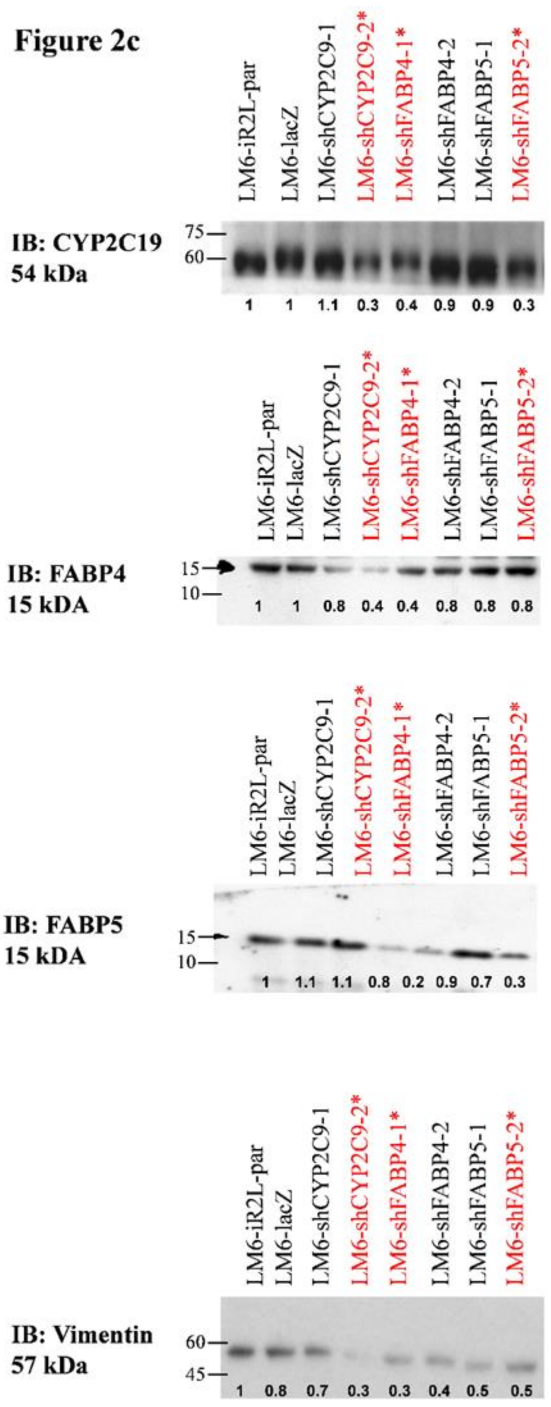

Figure S19. Representative Western blot image corresponding to Figure 2c showing all protein bands (CYP2C19, FABP4, FABP5 and vimentin) and molecular weight markers. Densitometry quantification of three independent experiments was performed using ImageJ.

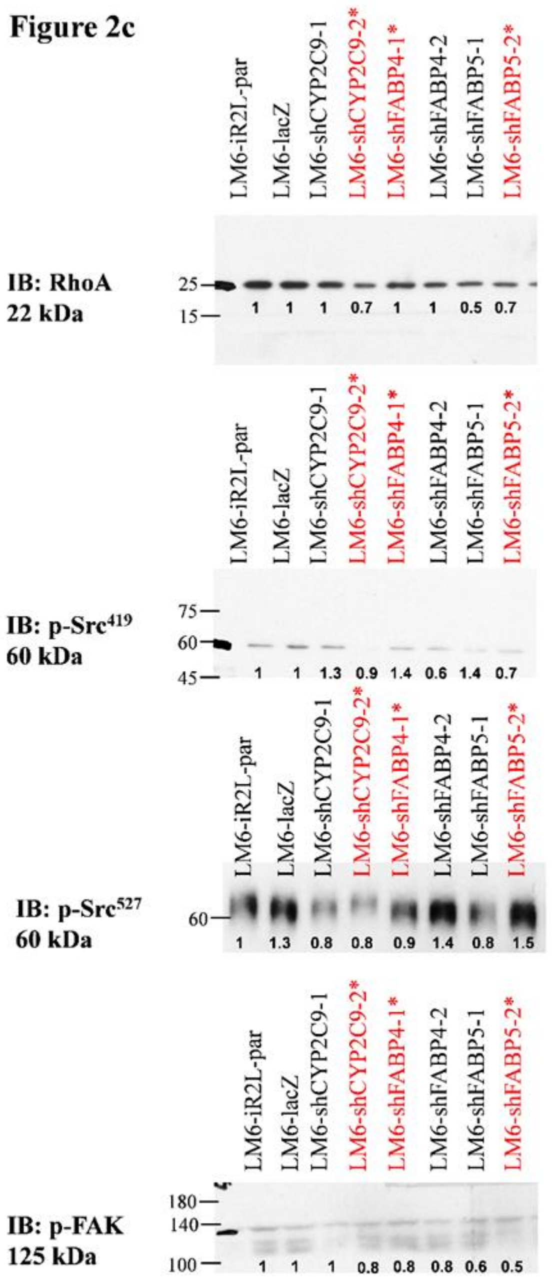

Figure S20. Representative Western blot image corresponding to Figure 2c showing all protein bands (RhoA, p-Src^{419}, p-Src^{527} and p-FAK) and molecular weight markers. Densitometry quantification of three independent experiments was performed using ImageJ.

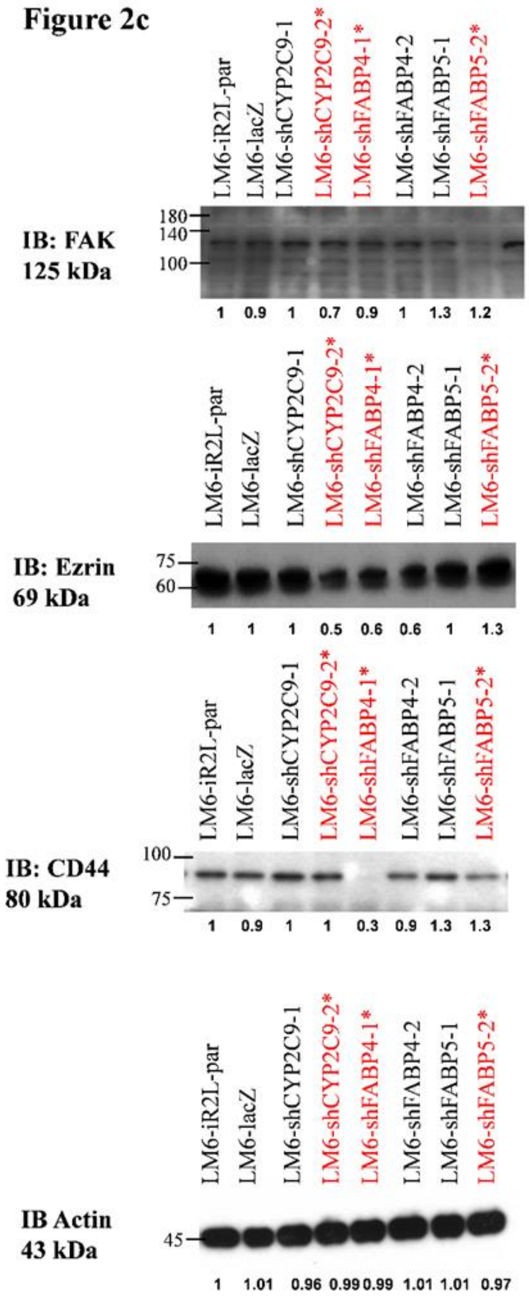

Figure S21. Representative Western blot image corresponding to Figure 2c showing all protein bands (FAK, ezrin, CD44 and actin) and molecular weight markers. Densitometry quantification of three independent experiments was performed using ImageJ.

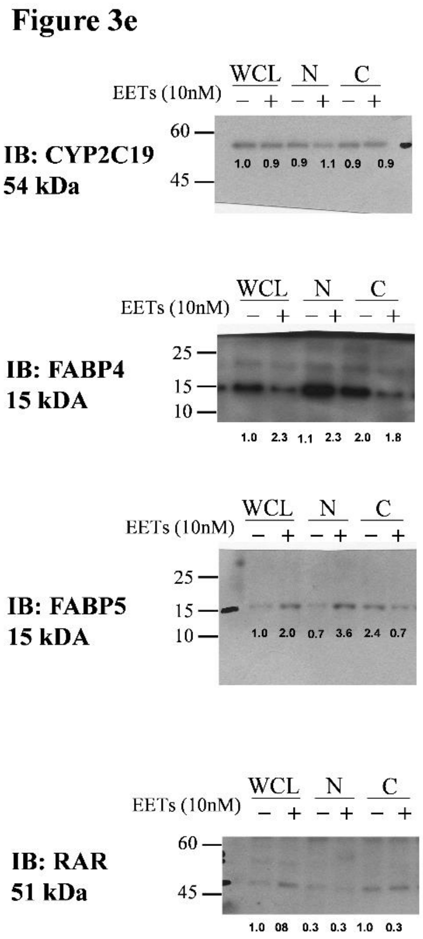

Figure S22. Representative Western blot image corresponding to Figure showing all protein bands 3e (CYP2C19, FABP4, FABP5 and RAR) and molecular weight markers. Densitometry quantification of three independent experiments was performed using ImageJ.

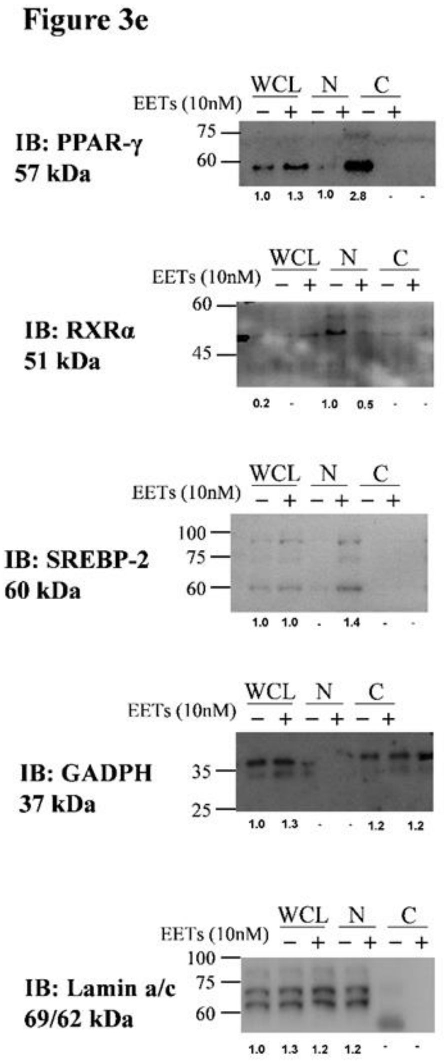

Figure S23. Representative Western blot image corresponding to Figure 3e showing all protein bands (PPAR-γ, RXRα, SREBP-2, GADPH and lamin ac/c) and molecular weight markers. Densitometry quantification of three independent experiments was performed using ImageJ.

Figure 3f

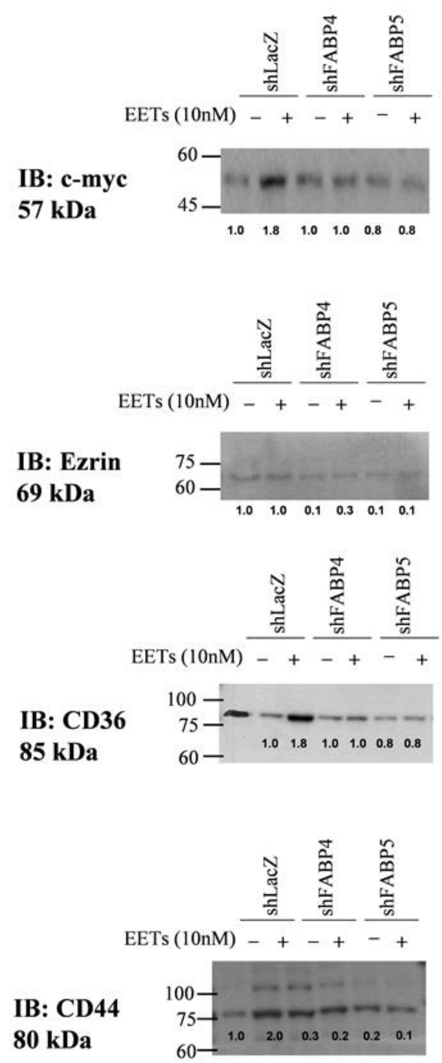

Figure S24. Representative Western blot image corresponding to Figure 3f showing all protein bands (c-myc, ezrin, CD36 and CD44) and molecular weight markers. Densitometry quantification of three independent experiments was performed using ImageJ.

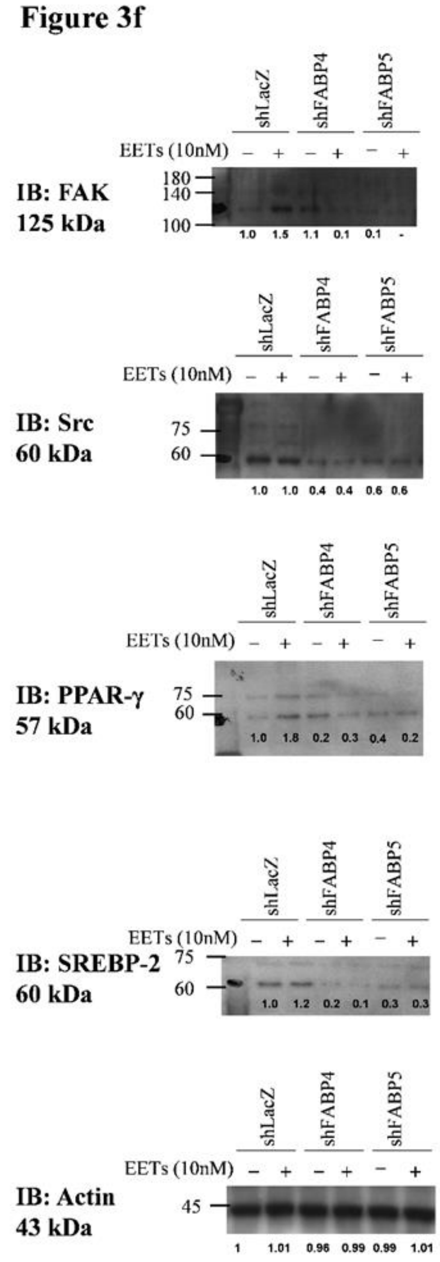

Figure S25. Representative Western blot image corresponding to Figure showing all protein bands 3f (FAK, Src, PPAR-γ, SREBP-2 and actin) and molecular weight markers. Densitometry quantification of three independent experiments was performed using ImageJ.

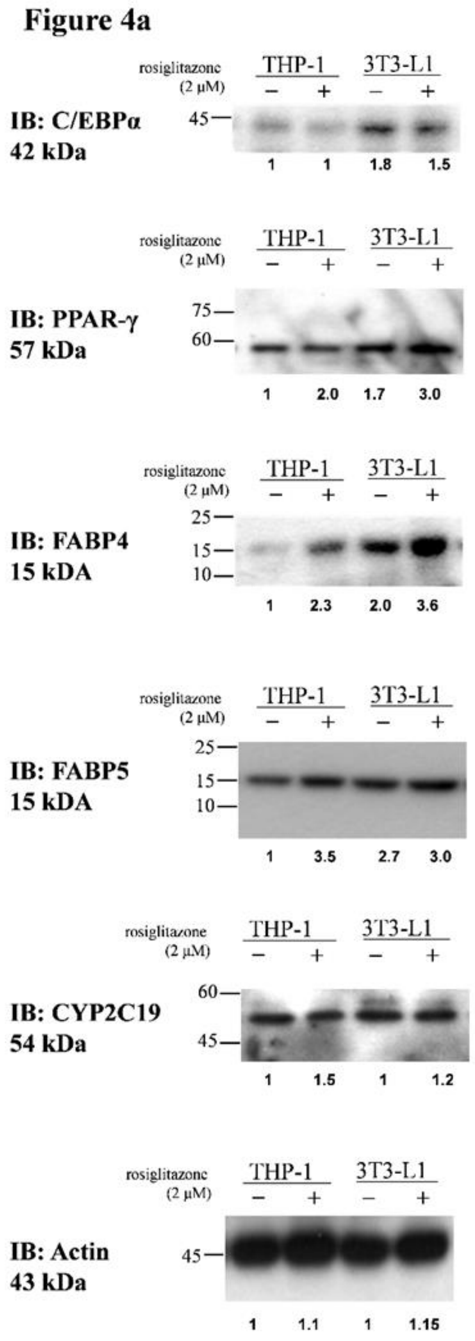

Figure S26. Representative Western blot image corresponding to Figure 4a showing all protein bands (C/EBPα, PPAR-γ, FABP4, FABP5, CYP2C19 and actin) and molecular weight markers. Densitometry quantification of three independent experiments was performed using ImageJ.

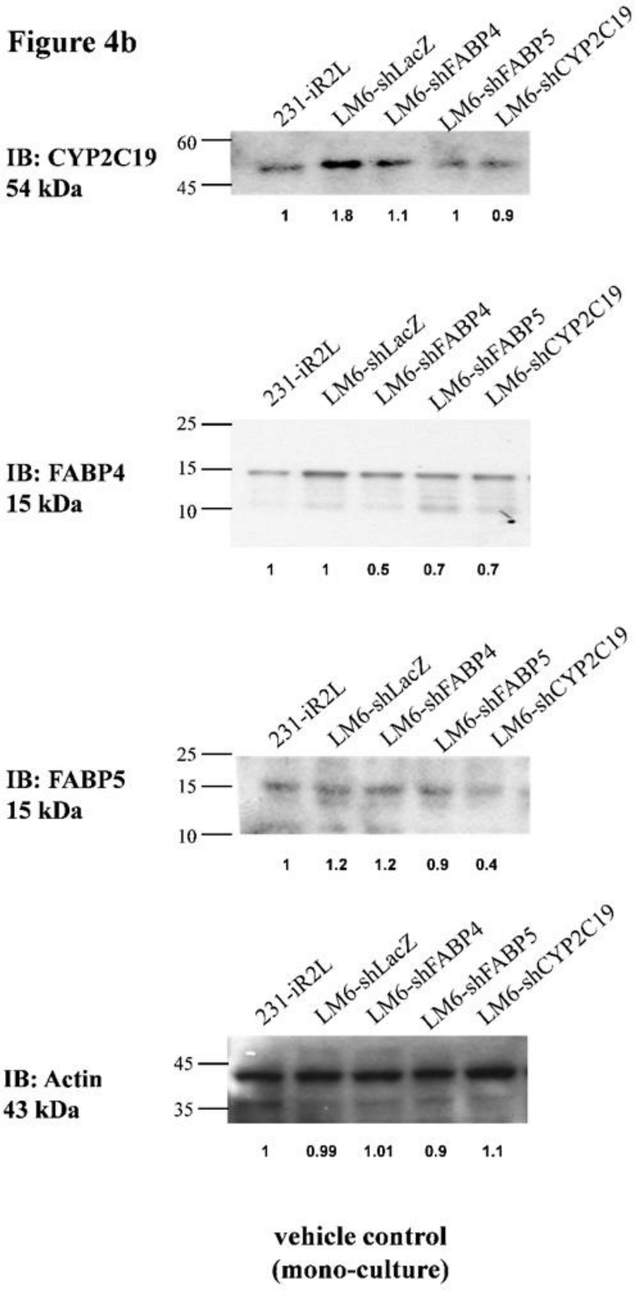

Figure S27. Representative Western blot image corresponding to Figure 4b (mono-culture) showing all protein bands (CYP2C19, FABP4, FABP5 and actin) and molecular weight markers. Densitometry quantification of three independent experiments was performed using ImageJ.

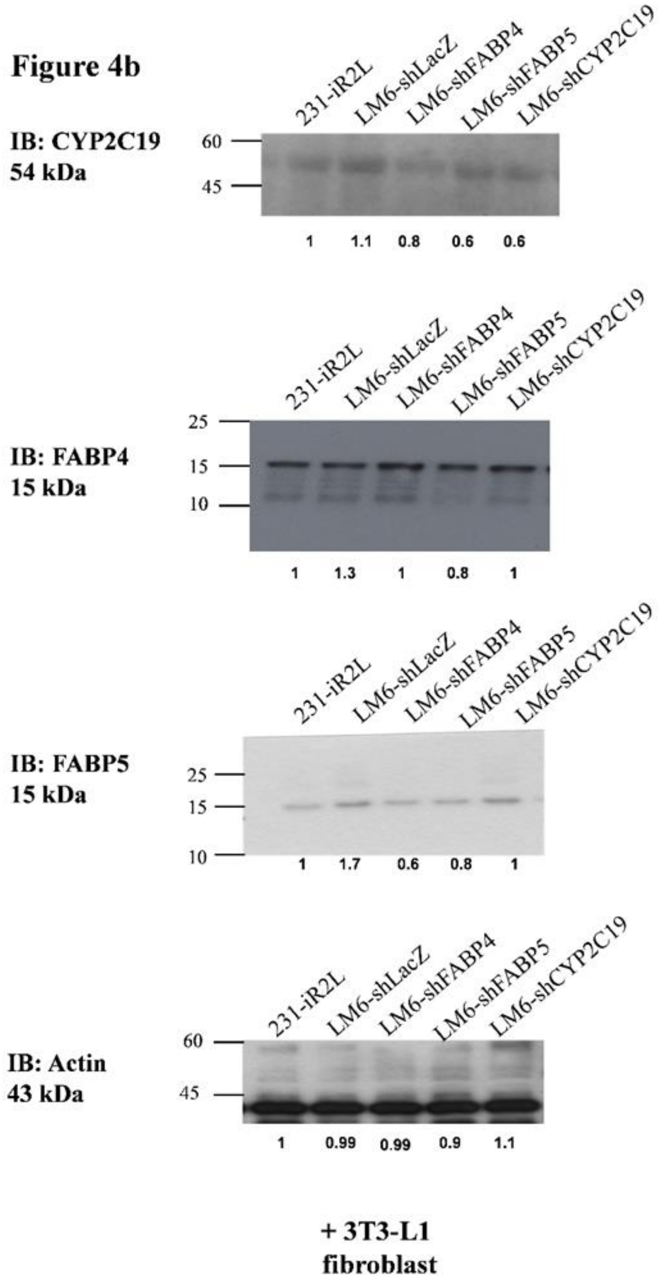

Figure S28. Representative Western blot image corresponding to Figure 4b (co-cultured with fibroblasts) showing all protein bands (CYP2C19, FABP4, FABP5 and actin) and molecular weight markers. Densitometry quantification of three independent experiments was performed using ImageJ.

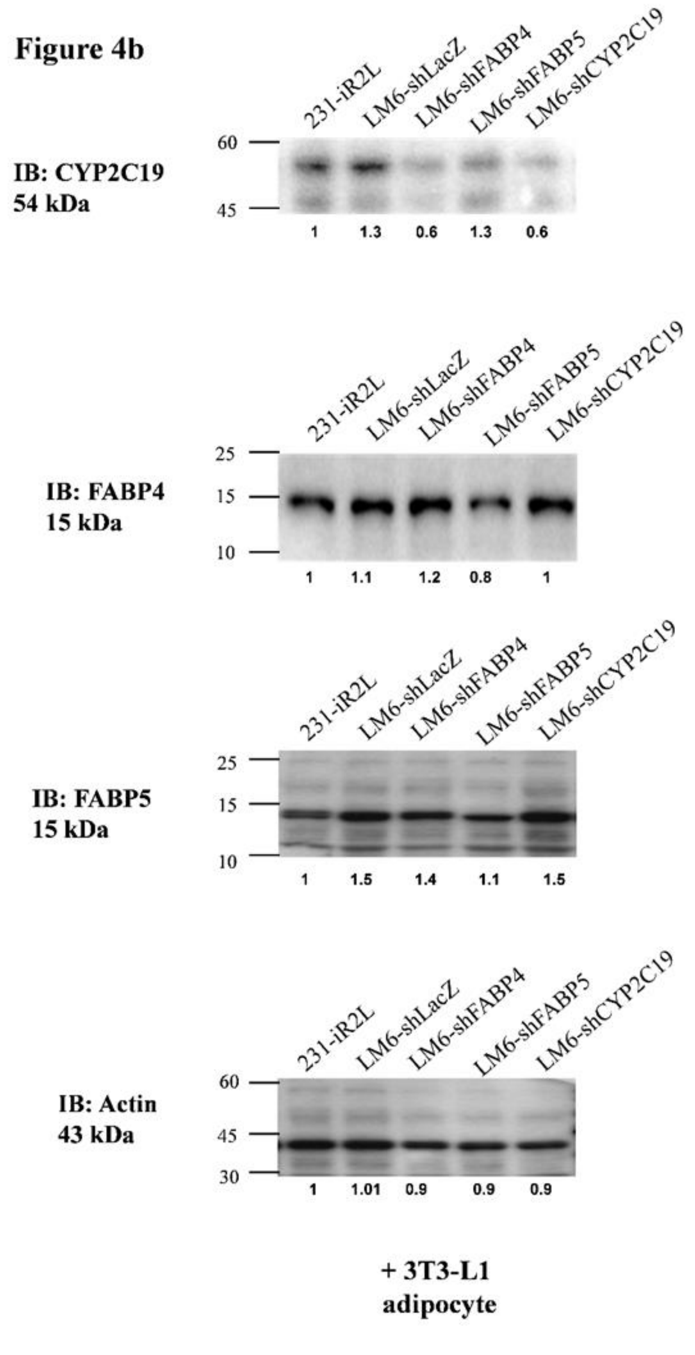

Figure S29. Representative Western blot image corresponding to Figure 4b (co-cultured with adipocytes) showing all protein bands (CYP2C19, FABP4, FABP5 and actin) and molecular weight markers. Densitometry quantification of three independent experiments was performed using ImageJ.

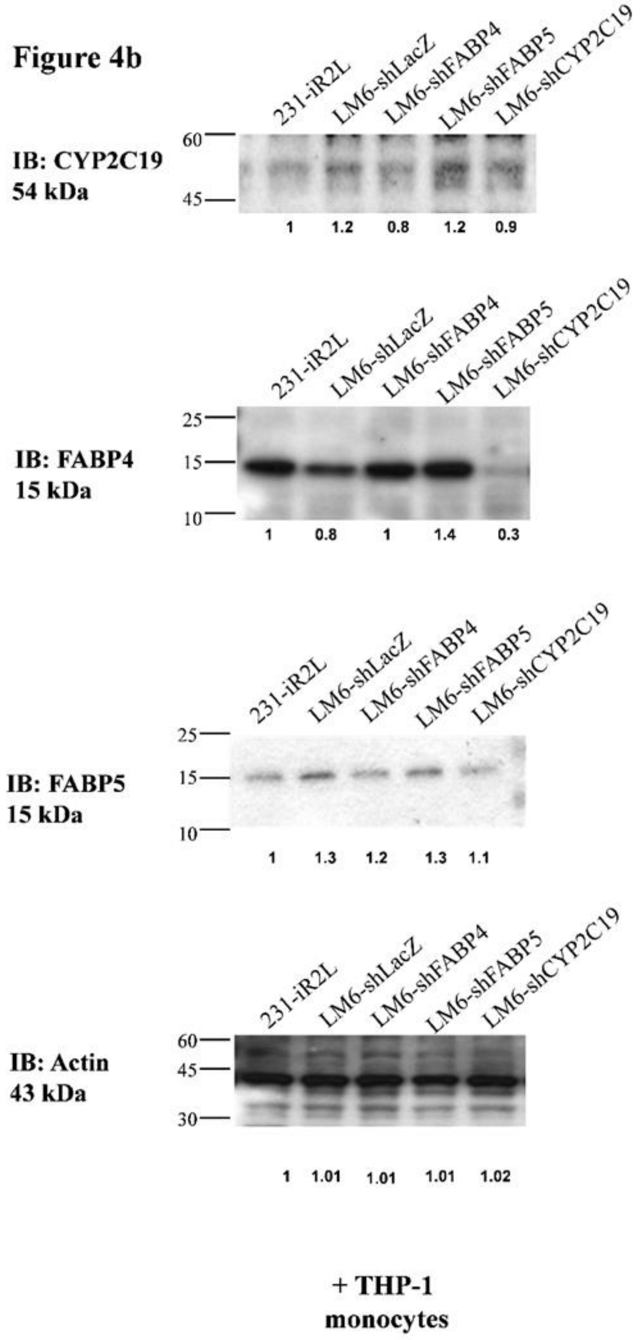

Figure S30. Representative Western blot image corresponding to Figure 4b (co-cultured with monocytes) showing all protein bands (CYP2C19, FABP4, FABP5 and actin) and molecular weight markers. Densitometry quantification of three independent experiments was performed using ImageJ.

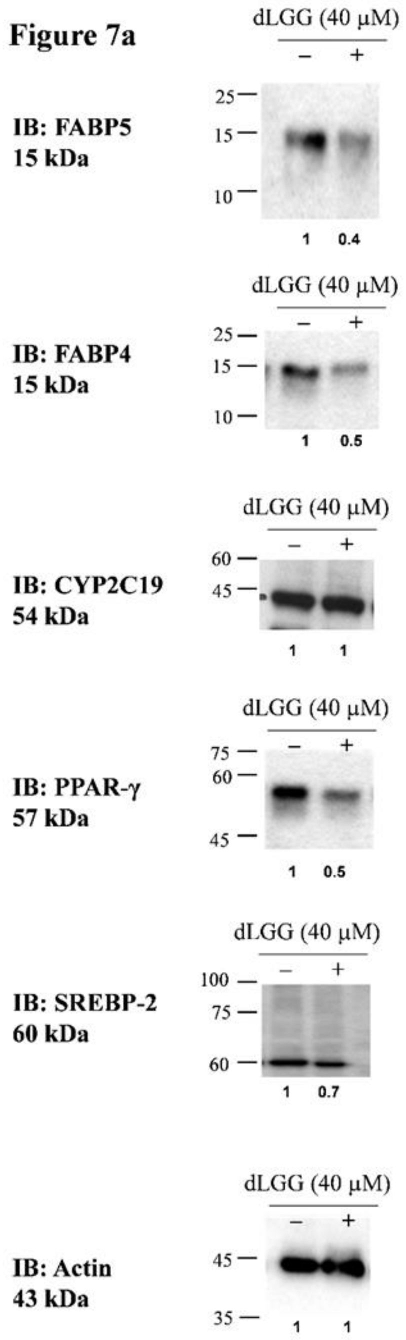

Figure S31. Representative Western blot image corresponding to Figure 7a showing all protein bands (FABP5, FABP5, CYP2C19, PPAR-γ, SREBP-2 and actin) and molecular weight markers. Densitometry quantification of three independent experiments was performed using ImageJ.

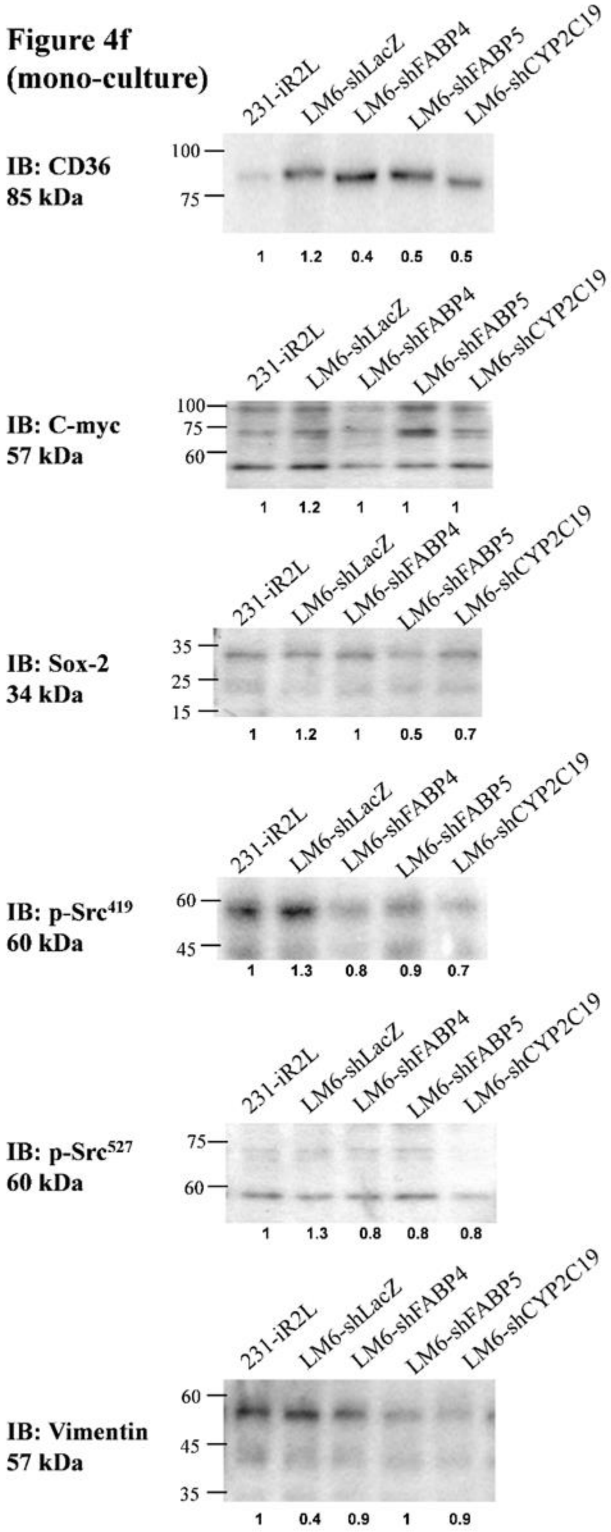

Figure S32. Representative Western blot image corresponding to Figure 4f (cells grown in mono-culture) showing all protein bands (CD36, c-myc, Sox2, p-Src^{419}, p-Src^{527} and vimentin) and molecular weight markers. Densitometry quantification of three independent experiments was performed using ImageJ.

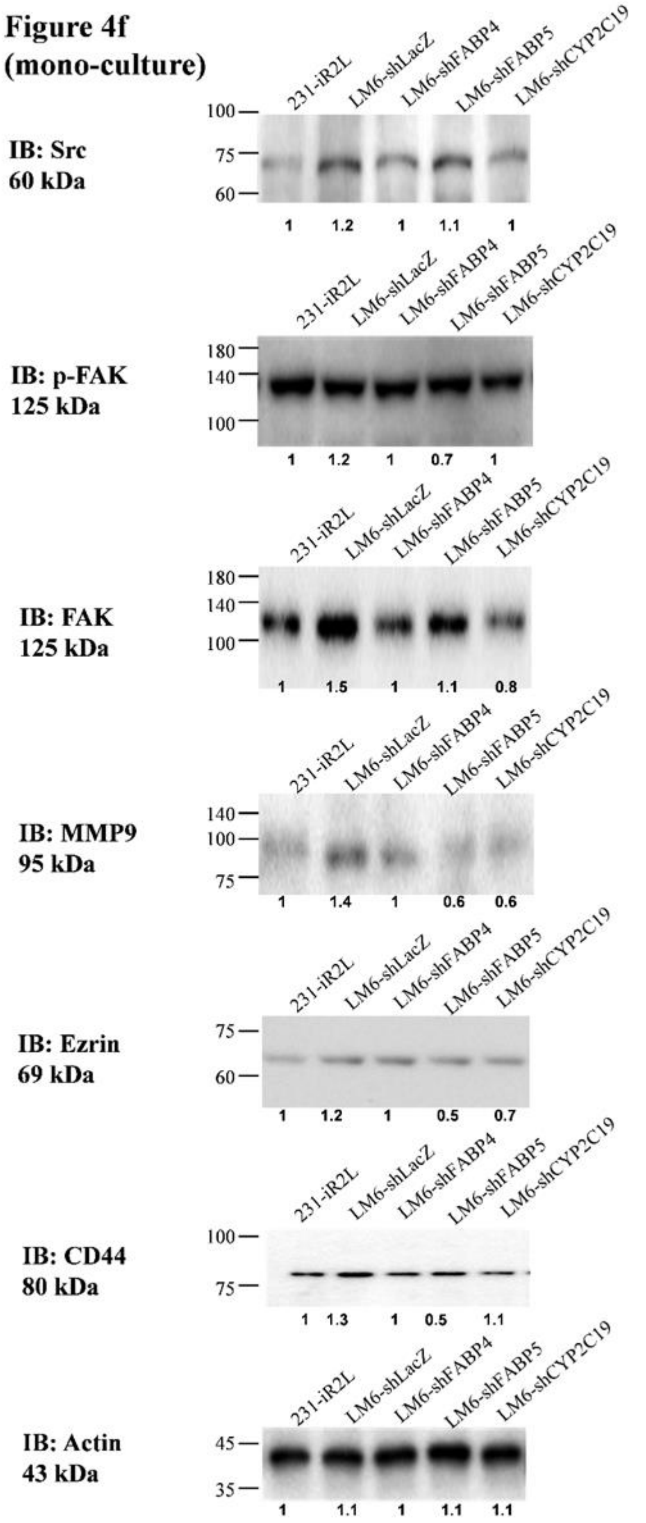

Figure S33. Representative Western blot image corresponding to Figure 4f (cells grown in mono-culture) showing all protein bands (Src, p-FAK, FAK, CD44, ezrin, MMP9 and actin) and molecular weight markers. Densitometry quantification of three independent experiments was performed using ImageJ.

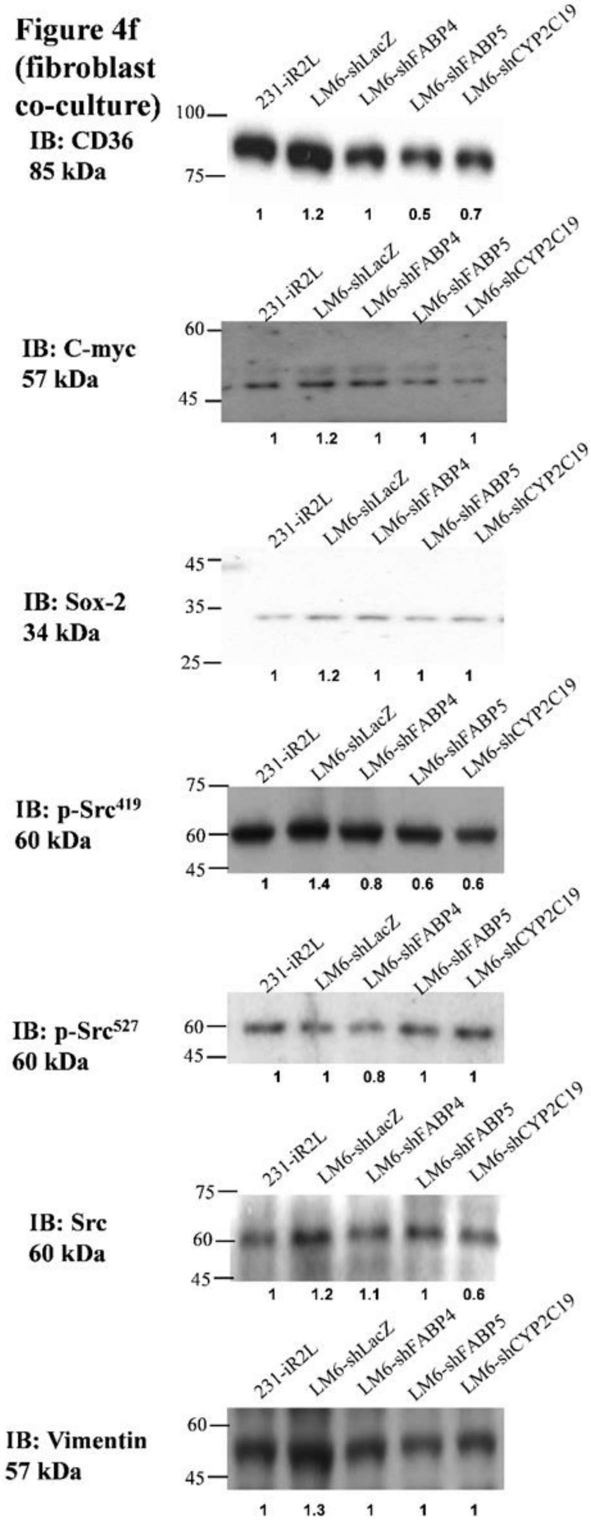

Figure S34. Representative Western blot image corresponding to Figure 4f (cells co-cultured with fibroblasts) showing all protein bands (CD36, c-myc, Sox2, p-Src^{419}, p-Src^{527}, Src and vimentin) and molecular weight markers. Densitometry quantification of three independent experiments was performed using ImageJ.

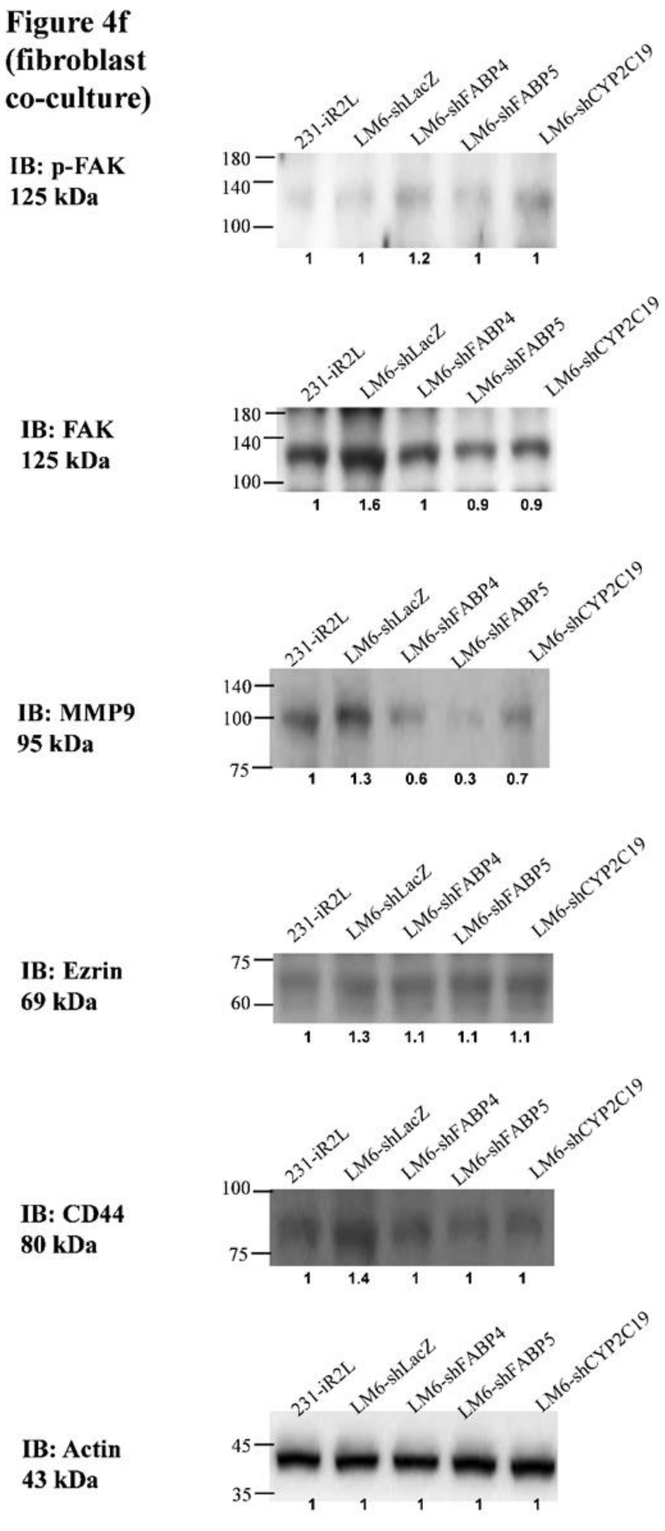

Figure S35. Representative Western blot image corresponding to Figure 4f (cells co-cultured with fibroblasts) showing all protein bands (p-FAK, FAK, CD44, ezrin, MMP9 and actin) and molecular weight markers. Densitometry quantification of three independent experiments was performed using ImageJ.

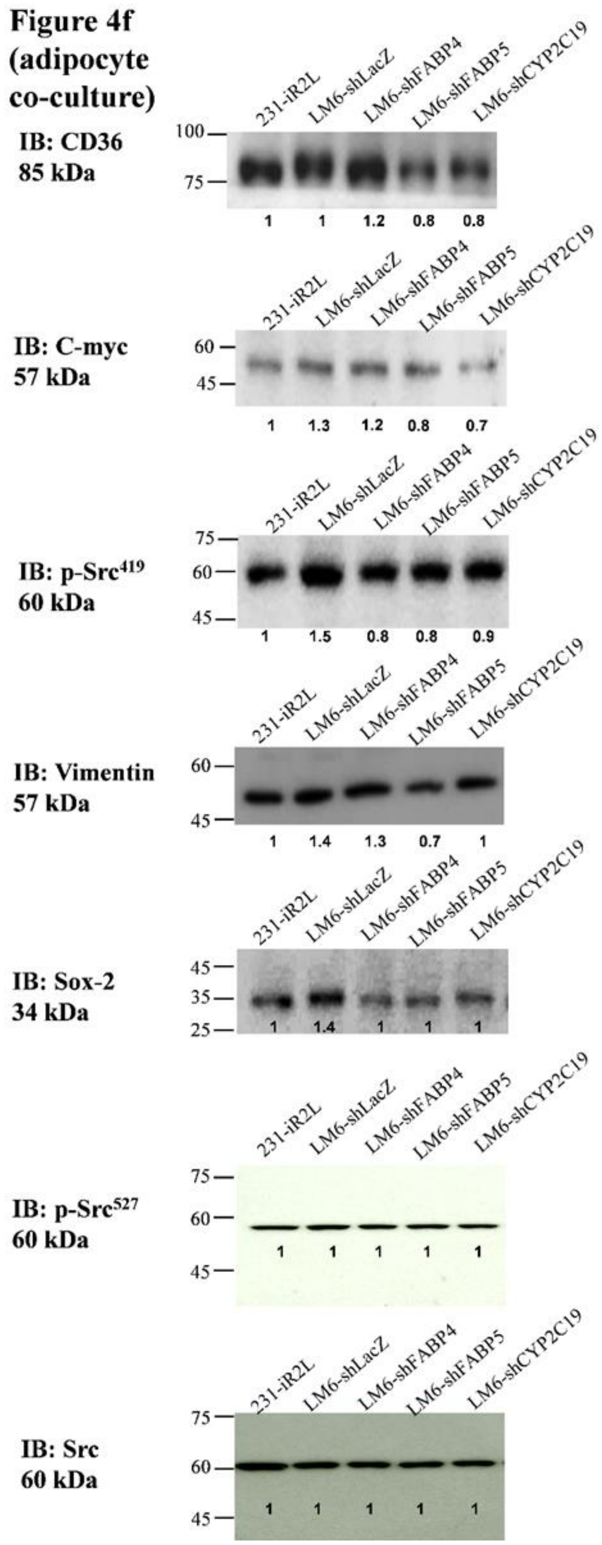

Figure S36. Representative Western blot image corresponding to Figure 4f (cells co-cultured with adipocytes) showing all protein bands (CD36, c-myc, Sox2, p-Src^{419}, p-Src^{527}, vimentin and Src) and molecular weight markers. Densitometry quantification of three independent experiments was performed using ImageJ.

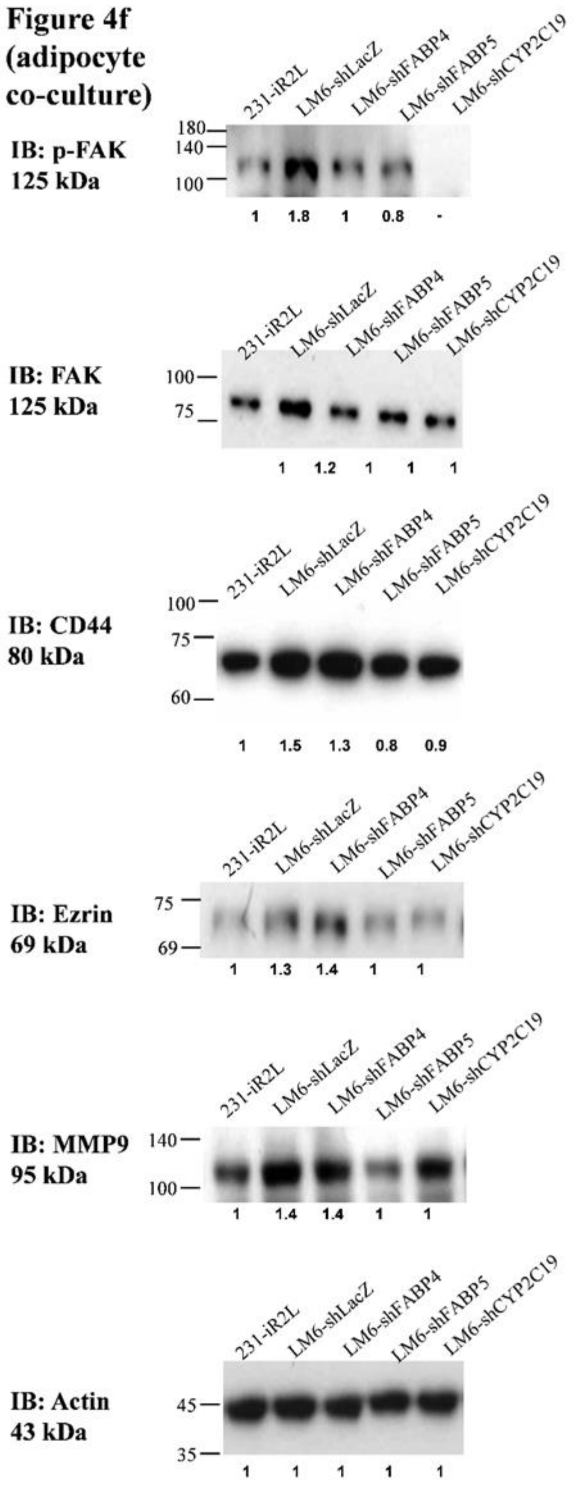

Figure S37. Representative Western blot image corresponding to Figure 4f (cells co-cultured with adipocytes) showing all protein bands (p-FAK, FAK, CD44, ezrin, MMP9 and actin) and molecular weight markers. Densitometry quantification of three independent experiments was performed using ImageJ.

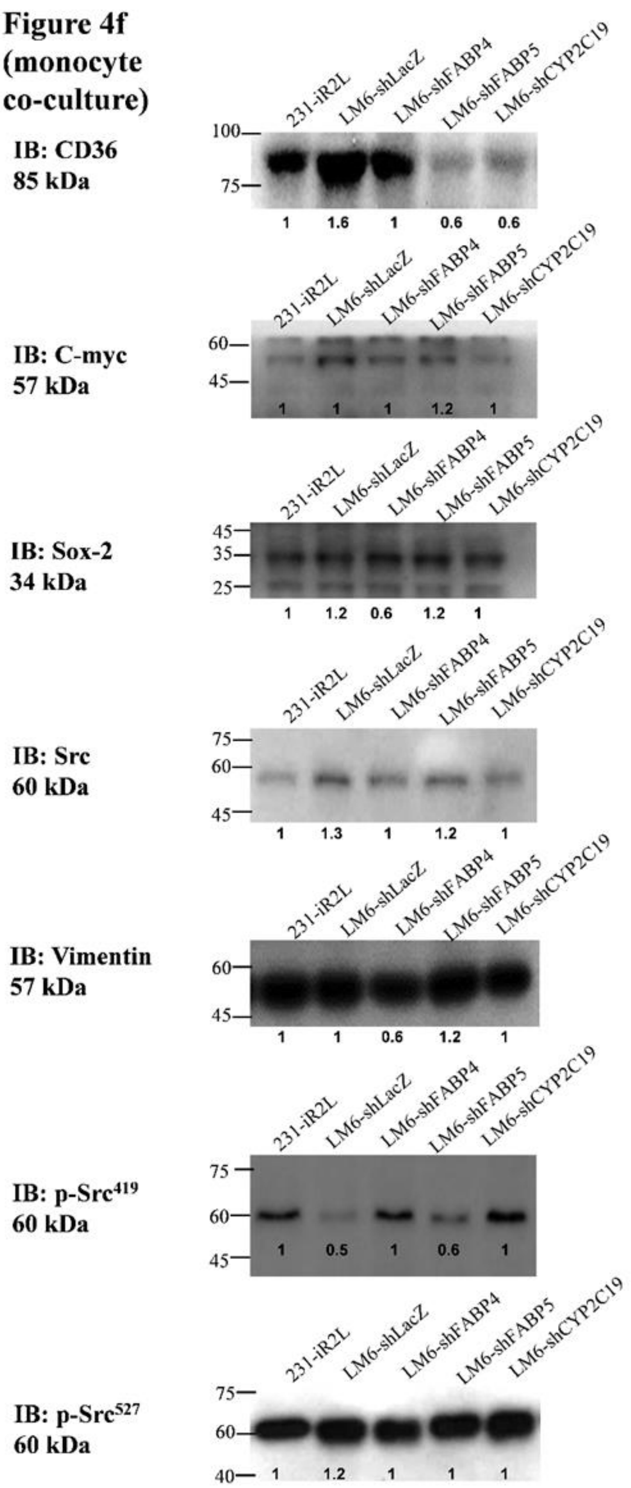

Figure S38. Representative Western blot image corresponding to Figure 4f (cells co-cultured with monocytes) showing all protein bands (CD36, c-myc, Sox2, Src, p-Src^{419}, p-Src^{527} and vimentin) and molecular weight markers. Densitometry quantification of three independent experiments was performed using ImageJ.

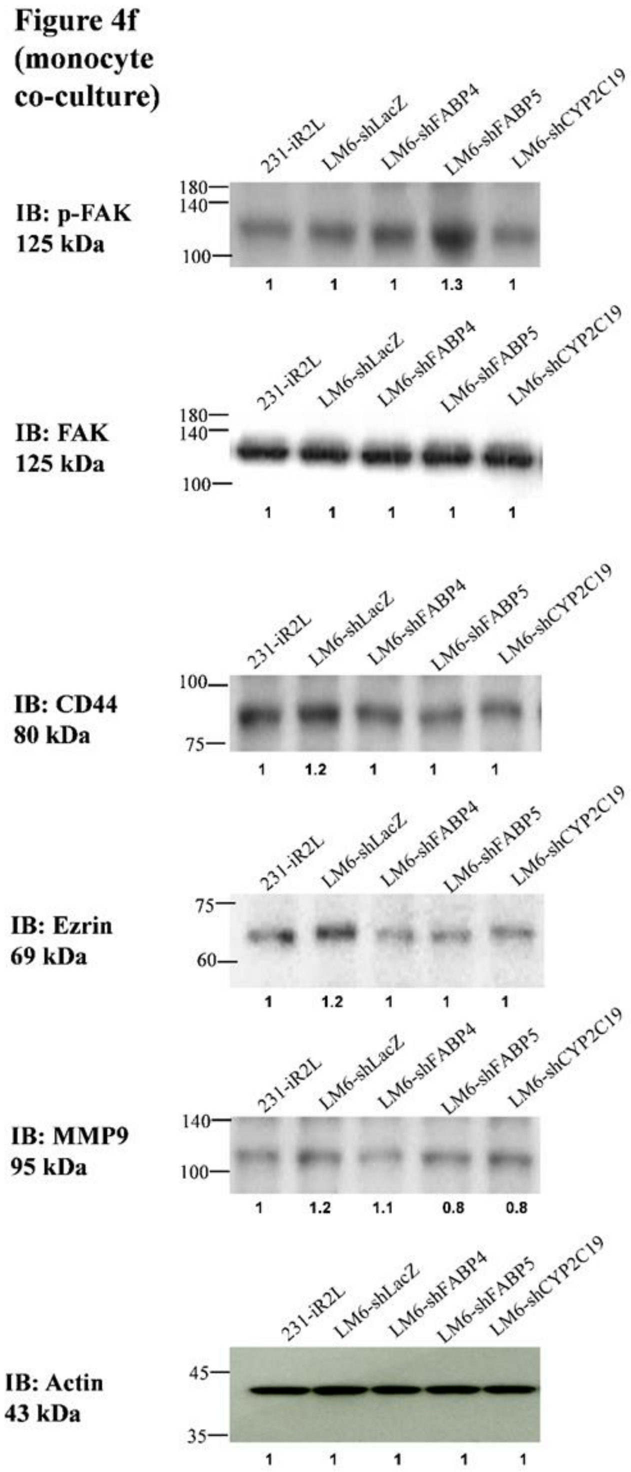

Figure S39. Representative Western blot image corresponding to Figure 4f (cells co-cultured with monocytes) showing all protein bands (p-FAK, FAK, CD44, ezrin, MMP9 and actin) and molecular weight markers. Densitometry quantification of three independent experiments was performed using ImageJ.

© 2020 by the authors. Licensee MDPI, Basel, Switzerland. This article is an open access article distributed under the terms and conditions of the Creative Commons Attribution (CC BY) license (http://creativecommons.org/licenses/by/4.0/).
